# Large-scale application of ClinGen-InSiGHT *APC*-specific ACMG/AMP variant classification criteria leads to substantial reduction in VUS

**DOI:** 10.1016/j.ajhg.2024.09.002

**Published:** 2024-10-01

**Authors:** Xiaoyu Yin, Marcy Richardson, Andreas Laner, Xuemei Shi, Elisabet Ognedal, Valeria Vasta, Thomas v.O. Hansen, Marta Pineda, Deborah Ritter, Johan de Dunnen, Emadeldin Hassanin, Wencong Lyman Lin, Ester Borras, Karl Krahn, Margareta Nordling, Alexandra Martins, Khalid Mahmood, Emily Nadeau, Victoria Beshay, Carli Tops, Maurizio Genuardi, Tina Pesaran, Ian M. Frayling, Gabriel Capellá, Andrew Latchford, Sean V. Tavtigian, Carlo Maj, Sharon E. Plon, Marc S. Greenblatt, Finlay A. Macrae, Isabel Spier, Stefan Aretz

**Affiliations:** 1 Department of Colorectal Medicine and Genetics, Royal Melbourne Hospital, Parkville, VIC, Australia; 2 Department of Medicine, University of Melbourne, Parkville, VIC, Australia; 3 Institute of Human Genetics, Medical Faculty, University of Bonn, Bonn, Germany; 4 Ambry Genetics, Aliso Viejo, CA, USA; 5 Medical Genetics Center Munich, MGZ Munich, Germany; 6 Greenwood Genetic Center, Greenwood, SC, USA; 7 Western Norway Familial Cancer Center, Haukeland University Hospital, Bergen, Norway; 8 Northwest Genomics Center, Department of Genome Sciences, University of Washington, Seattle, WA, USA; 9 Department of Clinical Genetics, Rigshospitalet, Copenhagen University Hospital, Copenhagen, Denmark; 10 Department of Clinical Medicine, Faculty of Health and Medical Sciences, University of Copenhagen, Copenhagen, Denmark; 11 European Reference Network on Genetic Tumour Risk Syndromes (ERN GENTURIS), Nijmegen, the Netherlands; 12 Hereditary Cancer Program, Catalan Institute of Oncology – ONCOBELL, IDIBELL, Barcelona, Spain; 13 Centro de Investigación Biomédica en Red de Cáncer (CIBERONC), Instituto Salud Carlos III, Madrid, Spain; 14 Baylor College of Medicine, Houston, TX, USA; 15 Texas Children’s Cancer Center, Texas Children’s Hospital, Houston, TX, USA; 16 Departments of Human Genetics & Clinical Genetics, Leiden University Medical Center, Leiden, the Netherlands; 17 Institute for Genomic Statistics and Bioinformatics, University Hospital Bonn, Bonn, Germany; 18 Luxembourg Centre for Systems Biomedicine, University of Luxembourg, Esch-sur-Alzette, Luxembourg; 19 St Vincents Hospital Melbourne, East Melbourne, VIC, Australia; 20 Invitae Corporation, San Francisco, CA, USA; 21 GeneDx, Gaithersburg, MD, USA; 22 Department of Biomedical and Clinical Sciences, Linköping University, Linköping, Sweden; 23 Department of Clinical Genetics, Linköping University Hospital, Linköping, Sweden; 24 Université de Rouen Normandie, Inserm U1245, 76000 Rouen, France; 25 Colorectal Oncogenomics Group, Department of Clinical Pathology, University of Melbourne, Melbourne, VIC, Australia; 26 Department of Medicine, Larner College of Medicine, University of Vermont, Burlington, VT, USA; 27 Peter MacCallum Cancer Centre, Melbourne, VIC, Australia; 28 Fondazione Policlinico Universitario A. Gemelli IRCCS, and Dipartimento di Scienze della Vita e Sanità Pubblica, Università Cattolica del Sacro Cuore, Rome, Italy; 29 Polyposis Registry, St Mark’s Hospital, London, UK; 30 Inherited Tumour Syndromes Research Group, Institute of Cancer & Genetics, Cardiff University, Cardiff, UK; 31 National Centre for Colorectal Disease, St Vincent’s University Hospital, Dublin, Ireland; 32 Department of Surgery and Cancer, Imperial College, London, UK; 33 Huntsman Cancer Institute, University of Utah, Salt Lake City, UT, USA; 34 Department of Oncological Sciences, School of Medicine, University of Utah, Salt Lake City, UT, USA; 35 Centre for Human Genetics, University of Marburg, Marburg, Germany; 36 National Center for Hereditary Tumor Syndromes, University Hospital Bonn, Bonn, Germany

**Keywords:** ACMG/AMP variant classification guidelines, *Adenomatous polyposis coli*, *APC*, ClinGen, familial adenomatous polyposis, FAP, InSiGHT

## Abstract

Pathogenic constitutional *APC* variants underlie familial adenomatous polyposis, the most common hereditary gastrointestinal polyposis syndrome. To improve variant classification and resolve the interpretative challenges of variants of uncertain significance (VUSs), APC-specific variant classification criteria were developed by the ClinGen-InSiGHT Hereditary Colorectal Cancer/Polyposis Variant Curation Expert Panel (VCEP) based on the criteria of the American College of Medical Genetics and Genomics and the Association for Molecular Pathology (ACMG/AMP). A streamlined algorithm using the *APC*-specific criteria was developed and applied to assess all *APC* variants in ClinVar and the International Society for Gastrointestinal Hereditary Tumours (InSiGHT) international reference *APC* Leiden Open Variation Database (LOVD) variant database, which included a total of 10,228 unique *APC* variants. Among the ClinVar and LOVD variants with an initial classification of (likely) benign or (likely) pathogenic, 94% and 96% remained in their original categories, respectively. In contrast, 41% ClinVar and 61% LOVD VUSs were reclassified into clinically meaningful classes, the vast majority as (likely) benign. The total number of VUSs was reduced by 37%. In 24 out of 37 (65%) promising *APC* variants that remained VUS despite evidence for pathogenicity, a data-mining-driven work-up allowed their reclassification as (likely) pathogenic. These results demonstrated that the application of *APC*-specific criteria substantially reduced the number of VUSs in ClinVar and LOVD. The study also demonstrated the feasibility of a systematic approach to variant classification in large datasets, which might serve as a generalizable model for other gene- or disease-specific variant interpretation initiatives. It also allowed for the prioritization of VUSs that will benefit from in-depth evidence collection. This subset of *APC* variants was approved by the VCEP and made publicly available through ClinVar and LOVD for widespread clinical use.

## Graphical abstract

**Figure:**
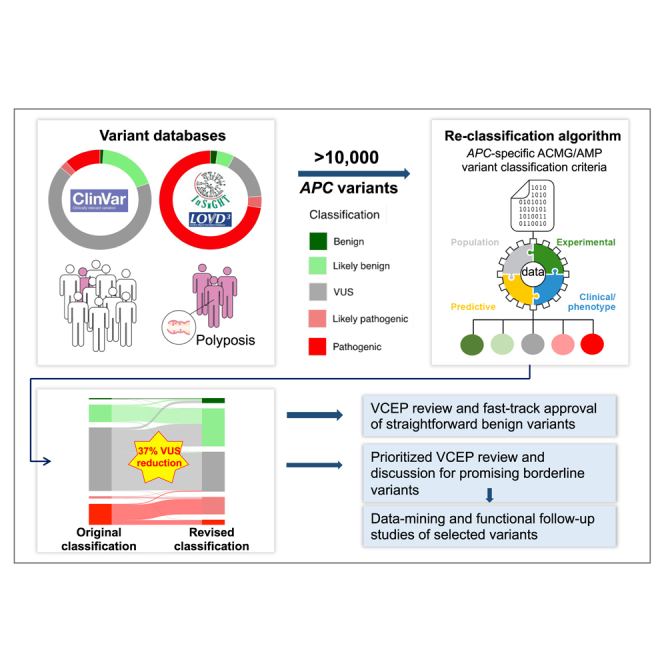

## Teaser

Application of the gene-specific ACMG/AMP variant classification criteria to >10,000 publicly available germline *APC* variants through a streamlined algorithm reduced the fraction of VUSs by 37%, demonstrating the feasibility of a systematic classification approach in large datasets. This study might serve as a model for other variant interpretation initiatives.

## Introduction

Familial adenomatous polyposis (FAP; MIM: 175100) is an autosomal-dominant precancerous condition and the most common monogenic gastrointestinal polyposis syndrome caused by constitutional (germline) pathogenic variants (PVs) in the tumor suppressor gene *APC* (MIM: 611731).^1^^,^^2^^,^^3^ The colorectal phenotype exhibits high inter- and intra-familial variability from the growth of less than 100 up to thousands of adenomatous polyps.^4^ Surveillance colonoscopy and/or prophylactic (procto)colectomy are warranted to prevent colorectal cancer or delay disease progression.^5^^,^^6^^,^^7^ The identification of an *APC* PV therefore has direct relevance for individuals and their relatives, defining *APC* as a highly clinically actionable gene.^8^ Depending on the colorectal phenotype and family history, causative *APC* variants can be identified in up to 85% of individuals with adenomatous polyposis,^4^^,^^9^^,^^10^^,^^11^^,^^12^^,^^13^ the vast majority of which are nonsense and frameshift variants leading to a truncated protein with abrogated function.^5^^,^^6^^,^^7^^,^^14^ During the last three decades, thousands of rare *APC* PVs have been identified in patients with FAP. Variants are distributed across the gene, the majority of which are private, observed in only one or very few families. Concurrently, the widespread use of large multi-gene panel testing and exome or genome sequencing have generated an additional plethora of *APC* variants in (healthy) individuals without a polyposis phenotype, many of which are missense alterations. In the absence of comprehensive data and consensus for the level of evidence required to corroborate variant interpretation, most of these variants remain variants of uncertain significance (VUSs) or variants with conflicting assertions, accounting for around 67% of *APC* variants in ClinVar. These VUSs confer diagnostic uncertainty and pose challenges in clinical practice.

Since its inception, the American College of Medical Genetics and Genomics and the Association for Molecular Pathology (ACMG/AMP) guidelines have evolved through further refinement to the various variant assessment methods and evidence codes^15^^,^^16^^,^^17^^,^^18^^,^^19^^,^^20^^,^^21^ and the development of gene- or disease-specific ACMG/AMP classification criteria by variant curation expert panels (VCEPs) under the governance of ClinGen (Clinical Genome Resource).^22^ The International Society for Gastrointestinal Hereditary Tumours (InSiGHT) houses and curates the world’s largest databases for variants of gastrointestinal-cancer-predisposing genes in the Leiden Open Variation Database (LOVD).^23^ Recently, a ClinGen-InSiGHT Hereditary Colorectal Cancer/Polyposis VCEP (HCCP VCEP) was established. The *APC* subcommittee (*APC* VCEP) developed and validated *APC*-specific ACMG/AMP classification criteria,^24^ readying the VCEP for variant submissions to ClinVar as an FDA-recognized expert panel. The most updated version of the VCEP specifications can be found in the online criteria specification registry.

In this study, we used the *APC*-specific criteria to perform a large-scale reclassification exercise of all *APC* variants listed in ClinVar and the InSiGHT *APC* reference database LOVD. The criteria were embedded and applied in a streamlined algorithm, which was supplemented by further data mining and curation to achieve the most accurate classification. The results were compared to their original assertions in respective databases, and any discrepancies were addressed.

## Methods

### *APC* variant database merging and centralization

Prior to the extraction of variants, the landscape of all publicly available databases containing *APC* variants was identified and examined for activity and curation status. Of at least 19 *APC* databases, nine are inactive and another three are not curated (Table S1). All listed curators, in particular those of inactive, outdated, or orphaned databases, were contacted to request sharing and merging of data with the reference LOVD (v.3.0) installation. To establish a centralized, curated data source of *APC* variants with consistent reporting format and phenotypic description, the InSiGHT *APC* LOVD and the Global Variome shared LOVD were subsequently merged to generate one international reference *APC* variant database in LOVD, abbreviated in the following as LOVD and accessible via all three URLs.

### ClinVar and LOVD variant extraction and annotation

ClinVar variants with summary evaluation and individual submitter annotations were retrieved from ClinVar in March 2022 (https://ftp.ncbi.nlm.nih.gov/pub/clinvar/xml/). The complete public dataset was downloaded, and all alleles associated with *APC* were extracted. The merged *APC* LOVD database was downloaded on May 12, 2022. The legacy description of published variants was recorded alongside their standardized nomenclature as per the Human Genome Variation Society (HGVS) guidelines on the preferred reference transcript GenBank: NM_000038.6,^25^ correcting for any errors where possible. All non-structural variants were annotated using the Ensembl Variant Effect Predictor (VEP).^26^ Structural variants defined by genomic alterations greater than 50 bp in size (gross deletions, duplications, inversions, in-frame, Alu and SVA retrotransposon insertions, inversions, and complex variants) were annotated manually using Mutalyzer.^27^

### Reclassification algorithm

**Figure 1:**
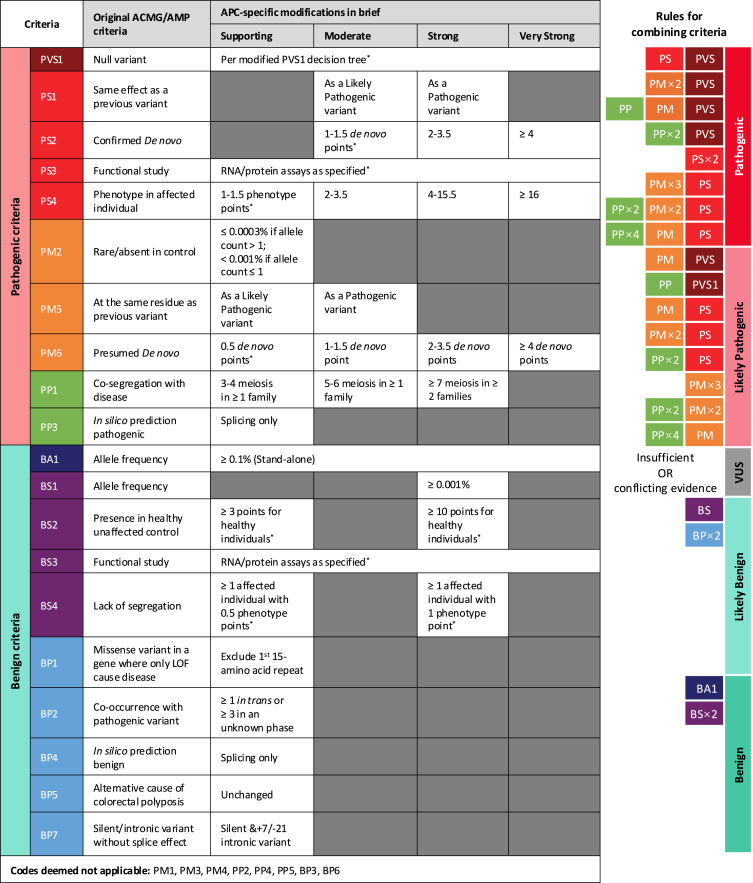
*APC*-specific criteria in brief The ACMG/AMP guidelines defined pathogenic (P) and benign (B) criteria encompassing evidence in population, experimental, computational, and clinical domains.^28^ The criteria are weighed and coded as benign stand-alone (BA), pathogenic very strong (PVS), strong (BS/PS), moderate (PM/BM), and supporting (PP/BP), the combination of which leads to a final classification of pathogenic (P), likely pathogenic (LP), variant of uncertain significance (VUS), likely benign (LB), or benign (B). This figure is only intended as a quick reference guide to the *APC*-specific criteria^24^; the most updated version can be found at https://cspec.genome.network/cspec/ui/svi/doc/GN089. ^∗^Details not shown here.

**Figure 2:**
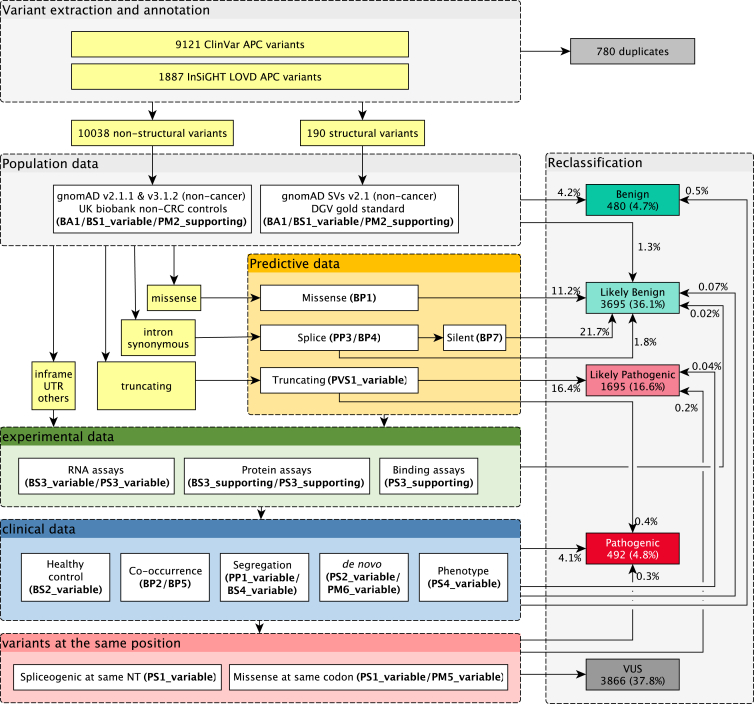
*APC-*specific criteria embedded in a reclassification algorithm An algorithm demonstrating the application of all eligible *APC-*specific codes to *APC* variants in ClinVar and the InSiGHT LOVD in a stepwise approach, and the percentage of variants that reached a B/LB or P/LP classification at each step. Firstly, the highest MAF of non-structural and structural variants was calculated from gnomAD non-cancer datasets or UK Biobank non-colorectal cancer control data, and gnomAD structural variants (SVs) or database of genomic variants (DGV) gold standard, respectively. Predictive criteria were then applied based on the most severe variant consequence as predicted by Ensemble variant effect predictor (VEP).^26^ Variants with any experimental and/or clinical evidence were identified and assigned corresponding code. Finally, splice variants at the same nucleotide and missense variants at the same codon were identified, and the variants at the same position criteria were applied.

Details of the *APC-*specific criteria were as published previously and summarized in Figure 1.^24^ A stepwise algorithm encompassing all evidence codes was designed to systematically evaluate all *APC* variants in ClinVar and LOVD (Figure 2).

### Minor allele frequency data (BA1, BS1, PM2_supporting)

The frequency of all *APC* variants in reference populations were compared against the minor allele frequency (MAF) criteria BA1 (≥0.001), BS1 (≥0.00001), and PM2_supporting (≤0.000003 or absent; first version of criteria). The non-cancer datasets from gnomAD (the Genome Aggregation Database) v2.1.1 and v3.1.2 were used as the reference population frequency data for non-structural variants.^29^ Exome sequencing data from 323,228 healthy individuals without a diagnosis of colorectal cancer (CRC) in the UK Biobank were also used to further enhance the detection of rare *APC* variants.^30^ If a variant was present in multiple reference population datasets, the highest MAF was calculated from any subpopulation with more than 2,000 alleles, with the exclusion of founder populations. The frequency of structural variants was examined in gnomAD structural variants (SVs) v2.1 and the Database of Genomic Variants (DGV) Gold Standard release from May 15, 2016.^31^

### Predictive data (PVS1, PP3, BP1, BP4, and BP7)

Truncating variants, canonical ±1/2 splice site variants, and exonic last nucleotide guanine to non-guanine variants were assigned the loss-of-function (LoF) criterion PVS1 if they are located between codons 49 and 2645 inclusive.^24^ Splice prediction was performed using SpliceAI and MaxEntScan via VEP, which determined PP3 and BP4 eligibility for synonymous and intronic variants and PP3 eligibility for presumed missense variants to reveal possible splicing effects.^32^^,^^33^ Variants exceeding a score of 0.6 in SpliceAI and a 15% reduction from the native site prediction in MaxEntScan were considered spliceogenic, and variants with a SpliceAI score of less than 0.2 and a MaxEntScan score of less than 3 were considered to have no impact on splicing. The criteria were only applied when both prediction tools showed concordant results. BP7 was subsequently applied to synonymous and deep intronic variants at or beyond +7/−21, which satisfied BP4. The missense code BP1 was applied to missense variants located outside of the first 15-amino-acid repeat of the β-catenin binding domain (codons 1021–1035) if they were consistently deemed non-spliceogenic by SpliceAI and MaxEntScan. Computational prediction models for conservation, evolution, etc. are not applicable for the evaluation of *APC* missense variants as described previously.^24^^,^^34^

### Experimental data (BS3, PS3)

Published mRNA splicing assays and protein function assays of *APC* variants were collated in a systematic review by the *APC* VCEP to derive gene-specific recommendations for the application of the experimental criteria BS3 and PS3.^24^ VCEP-approved experimental evidence for *APC* variant classifications included RNA assays for PS3 and BS3, β-catenin regulated transcriptional assays for PS3_supporting and BS3_supporting, and surface plasmon resonance assays for PS3_supporting. The proportion of aberrant transcripts, evidence of bi-allelic expression, and use of nonsense-mediated decay inhibition were also noted from the original publications when available, which determined the quality of the data and therefore the weight assigned for PS3 and BS3.

### Clinical data (PS4, PS2, PM6, PP1, BS4, BP2, BP5, BS2)

The phenotype details of individuals with *APC* variants from the InSiGHT LOVD download were retrieved. In individuals where phenotype details were recorded as unstructured text, text mining was employed to extract and stratify useful information. Affected individuals were scored for PS4 as described previously.^24^ Further data mining was undertaken focusing on the identification of confirmed *de novo APC* variants (PS2/PM6), segregation and non-segregation analysis (PP1/BS4), co-occurrence of the variant under assessment with other established pathogenic *APC* variant (BP2), or with an alternative molecular basis of disease (BP5), including heterozygous PVs in *POLD1* (MIM: 174761) or *POLE* (MIM: 174762); bi-allelic PVs in *MUTYH* (MIM: 604933), *NTHL1* (MIM: 602656), *MSH3* (MIM: 600887), or the MMR genes (*MLH1* [MIM: 120436], *MSH2* [MIM: 609309], *MSH6* [MIM: 600678], or *PMS2* MIM: 600259]). The HGMD and Universal Mutation Database were also examined for additional data.^35^^,^^36^ For variants that were already listed in gnomAD, their presence in healthy unaffected individuals in the UK Biobank non-CRC population was regarded as additional evidence in support of a benign classification (BS2). The absolute number of heterozygous individuals in the UK Biobank non-CRC dataset was counted, scored according to the *APC* VCEP’s definition for a healthy individual, and given the healthy control code BS2 with appropriate weight. As homozygous LoF *APC* variants were shown to be lethal at embryonic developmental stages,^37^ the observation of variant homozygosity ≥2 times in any reference population database was also considered strong evidence for a benign classification as defined in the *APC*-specific criteria. Clinical data were obtained in accordance with the guidelines of the Ethics Committee of the Medical Faculty of the University of Bonn and the 1975 Declaration of Helsinki. Participants of clinical genetic testing gave written informed consent for their data to be used for clinical research and genetic investigations according to local regulations.

### Statistical analysis

McNemar’s test is used to assess dichotomous changes between VUS vs. non-VUS classifications before and after reclassification to assess the impact of the *APC*-specific criteria. The Mann-Whitney-Wilcoxon test with continuity correction was used to compare differences in distribution of classification for selected variant types before and after reclassification. Statistical analyses were performed using R software version 4.3.2 (R Project for Statistical Computing). All significance tests were 2-tailed, and *p* < 0.05 was considered statistically significant.

### Review and synthesis of primary criteria combination

The *APC*-specific criteria applied for each variant were pulled together to generate a preliminary criteria combination according to the *APC* VCEP’s rules for combining criteria.^24^ In addition to the phenotype description in LOVD, for variants that fulfilled a reasonable set of predictive and/or experimental criteria (e.g., PVS1, PS3, or BS3) but remained unclassified, the corresponding internal clinical records and/or reference publication of the variant in question was consulted to curate additional phenotype points, and further data mining was conducted using Mastermind, LitVar, and PubMed as required to reach a non-VUS classification. Additionally, the classification of the 58 published *APC* variants in the pilot study for the development of the *APC*-specific guidelines were incorporated, adding another layer of data and expert review to the result. Finally, the revised criteria combination was used to calculate a final pathogenicity class. Missense variants at the same codon and spliceogenic variants at the same nucleotide position as established pathogenic (P) or likely pathogenic (LP) variants were identified, and the criteria PS1 or PM5 were applied accordingly.

### Prioritized list of variants for further review

**Figure 3:**
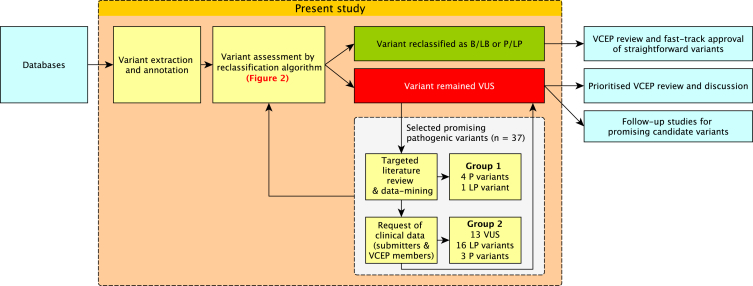
Reclassification workflow and suggested method of operation for ongoing VCEP activity This workflow summarized the organizational procedures undertaken in this study for variant reclassification and prioritization of variants with high clinical importance for different modes of VCEP review and approval. Relatively straightforward variants might be processed in batch and become candidates for fast-track VCEP approval (e.g., variants fulfilling BA1 or BS1 plus BP1). From the remaining 37 VUSs with some evidence for pathogenicity, five variants were reassessed as P/LP by a targeted literature review and data mining (group 1). The remaining 32 variants were reassessed based on further clinical information requested from respective ClinVar submitters and VCEP members, which ultimately lead to prioritized VCEP review.

The revised variant classification was compared with the original pathogenicity assertion in ClinVar and LOVD. Variants with clinically significant conflict (benign/likely benign [B/LB] vs. P/LP or VUS vs. P/LP) between the original assertion and reclassification were examined for causes of discrepancy. From the variants that remained VUSs after reclassification, 37 variants close to reaching a pathogenic classification were identified (Table 1), which included (1) truncating VUSs that fulfilled PVS1 but not PM2_supporting, (2) VUSs with pathogenic *in silico* predictions, (3) VUSs with experimental findings suggestive of deleterious effect, and (4) VUSs observed in individuals with FAP-associated phenotypes. A targeted literature search was conducted to acquire further information for all 37 variants. If a clinically relevant classification could be achieved, no further work up was done (group 1 in Table 1; Figure 3). Otherwise, a standardized form was used to ask VCEP members whether these variants were available in their internal laboratory databases, including clinical information (number of polyps, etc.) and RNA analysis data for variants with suspected splice effect (group 2 in Table 1; Figure 3).

**Table 1:** Further curation of selected variants remaining VUSs after application of reclassification algorithm

**HGVSc**	**Database ID**^a^	**Original classification**	**Criteria applied by algorithm**	**Classification by algorithm**	**Further curation**	**Final criteria applied**	**Final classification of VCEP**
**Group 1 previously pathogenic variants—further assessment based on literature review, data mining, and reassessment of minor allele frequency (MAF) criteria resulted in a pathogenic classification**							

c.471G>A (p.Trp157Ter)	ClinVar: 411479	pathogenic	PVS1	VUS	1.5 phenotype points^38^^,^^39^^,^^40^^,^^41^; revised MAF criteria	PVS1, PS4_supp, PM2_supp^b^	pathogenic
c.1312+4_1312+19del (p.?)	LOVD: APC_001939	pathogenic	BP4, PM2_supp	VUS	1 phenotype point, RNA assay result, segregated in 7 meioses^42^	PS3_mod, PS4_supp, PP1_mod, PM2_supp	likely pathogenic
c.1333C>T (p.Gln445Ter)	ClinVar: 438865	pathogenic	PVS1, BS1	VUS	1 phenotype point^43^^,^^44^; revised MAF criteria	PVS1, PS4_supp, PM2_supp^b^	pathogenic
c.2546_2551del (p.Asp849_Ser851delinsGly)	LOVD APC_000075	pathogenic	PM2_supp, PS4_mod	VUS	error in annotation: c.2546delATAGAAG (legacy name). Correct nomenclature: c.2546_2552del; p.(Asp849Valfs^∗^10), 1 phenotype point, segregation in 3 meioses^11^	PVS1, PS4_supp, PM2_supp, PP1	pathogenic
c.4669_4670del (p.Ile1557Ter)	ClinVar: 183857	pathogenic	PVS1	VUS	1 phenotype point^45^^,^^46^; revised MAF criteria	PVS1, PS4_supp, PM2_supp^b^	pathogenic

**Group 2 promising potentially pathogenic VUS—further assessment based on clinical evidence from ClinVar submitters and VCEP members and reassessment of MAF criteria**							

c.70C>T (p.Arg24Ter)	ClinVar: 184702	likely pathogenic	BS1	likely benign	1.5 phenotype points; >10 healthy individual points in total^47^^,^^48^; revised MAF criteria	PS4_supp, BS2	VUS
c.136−4A>G (p.?)	ClinVar: 925741	VUS	PP3, BS1	VUS	observed in 1 individual worth 0 phenotype points; revised MAF criteria	PM2_supp	VUS
c.156del (p.Gly53GlufsTer17)	ClinVar: 654864	pathogenic	PVS1	VUS	observed in 1 individual worth 0 phenotype points; revised MAF criteria	PVS1, PM2_supp^a^	likely pathogenic
c.203del (p.Leu68TyrfsTer2)	ClinVar: 934724	pathogenic	PVS1	VUS	observed in 4 individuals worth 1 phenotype point; revised MAF criteria	PVS1, PS4_supp, PM2_supp^b^	pathogenic
c.220+2T>A (p.?)	ClinVar: 141515	likely pathogenic	PVS1, BS1	VUS	observed in 35 individuals worth 8 phenotype points; revised MAF criteria	PVS1, PS4, PM2_supp^b^	pathogenic
c.422G>C (p.Arg141Thr)	ClinVar: 1056286	VUS	PVS1_strong, PM2_supp	VUS	observed in 3 individuals worth 0.5 phenotype points	PVS1_strong, PM2_supp	VUS
c.422G>A (p.Arg141Lys)	ClinVar: 824696	VUS	PVS1_strong	VUS	observed in 0 individual worth 0 phenotype points; revised MAF criteria	PVS1_strong, PM2_supp^b^	VUS
c.423−9A>G (p.?)	ClinVar: 469955	pathogenic	PP3, PM2_supp, PS3_mod	VUS	observed in 2 families worth 2 phenotype points	PP3, PM2_supp, PS3_mod, PS4_mod	likely pathogenic
c.531+5_531+8del (p.?)	ClinVar: 537529	likely pathogenic	PP3, PM2_supp, PS3_mod, PS4_supp	VUS	observed in 5 individuals worth 3.5 phenotype points^49^^,^^50^	PP3, PM2_supp, PS3_mod, PS4_mod	likely pathogenic
c.531+5G>A (p.?)	ClinVar: 127305	pathogenic	PP3, PM2_supp, PS4_supp, PS1_mod	VUS	observed in 6 individuals worth 2 phenotype points^51^^,^^52^^,^^53^	PP3, PM2_supp, PS4_mod, PS1_mod	likely pathogenic
c.531+6T>C (p.?)	ClinVar: 576816	VUS	PP3, PM2_supp, PS3_mod	VUS	observed in 2 individuals worth 2 phenotype points	PP3, PM2_supp, PS3_mod, PS4_mod	likely pathogenic
c.623A>G (p.Gln208Arg)	LOVD: APC_000758	pathogenic	BP1, PM2_supp, PP1	VUS	1 phenotype point^54^; not observed by VCEP members	BP1, PM2_supp, PS4_supp	VUS
c.645+2T>G (p.?)	ClinVar: 185659	likely pathogenic	PVS1_mod, PM2_supp	VUS	observed in 2 individuals worth 1 phenotype point	PVS1_mod, PM2_supp, PS4_supp	VUS
c.835−17A>G (p.?)	ClinVar: 822326	likely pathogenic	PM2_supp, PS3_mod	VUS	observed in 4 individuals worth 0 phenotype points	PM2_supp, PS3_supp	VUS
c.835−7T>G (p.?)	ClinVar: 433614	likely pathogenic	PP3, PM2_supp, PS3_mod	VUS	observed in 3 families worth 2.5 phenotype points	PP3, PM2_supp, PS3_mod, PS4_mod	likely pathogenic
c.933G>C (p.Lys311Asn)	ClinVar: 1025291	likely pathogenic	PVS1_supp, PM2_supp	VUS	observed in 2 individuals worth 1.5 phenotype points^55^	PVS1_strong, PM2_supp, PS4_supp	likely pathogenic
c.1042C>T (p.?)^c^	ClinVar: 955439	conflicting (pathogenic/VUS)	PVS1	VUS	1 phenotype point^56^^,^^57^; not observed by VCEP members; revised MAF criteria	PS4_supp, PM2_supp^b^, BS2_supp	VUS
c.1312+3A>C (p.?)	ClinVar: 486792	likely pathogenic	PP3, PM2_supp, PS1_mod	VUS	observed in 2 individuals worth 2 phenotype points^58^	PP3, PM2_supp, PS1, PS4_mod	likely pathogenic
c.1312+5G>C (p.?)	ClinVar: 265372	likely pathogenic	PM2_supp, PS4_supp, PS1_mod	VUS	observed in 3 individuals worth 2.5 phenotype points	PM2_supp, PS4_mod, PS1_mod, PP3	likely pathogenic
c.1408+735A>T (p.?)	LOVD: APC_001244	pathogenic	PM2_supp, PS3_mod, PS4_supp	VUS	1 phenotype point^59^; not observed by VCEP members	PS4_supp, PS3_mod, PM2_supp	VUS
c.1409−5A>G (p.?)	ClinVar: 411406	pathogenic	PP3, PM2_supp, PS3_mod, PS4_supp	VUS	observed in 5 individuals worth 3 phenotype points	PS3_mod, PS4_mod, PP3, PM2_supp	likely pathogenic
c.1409−3T>G (p.?)	ClinVar: 485146	likely pathogenic	PP3, PM2_supp, PS3_mod	VUS	observed in 4 individuals worth 2 phenotype points^55^	PS3_mod, PS4_mod, PP3, PM2_supp	likely pathogenic
c.1743G>C (p.Lys581Asn)	ClinVar: 428153	likely pathogenic	PVS1_strong, PM2_supp	VUS	observed in 7 individuals worth 1 phenotype point	PVS1_strong, PS4_supp, PM2_supp	likely pathogenic
c.1902T>G (p.Ser634Arg)	ClinVar: 231954	conflicting (likely pathogenic/VUS)	BP1, PS3_mod	VUS	observed in 2 individuals worth 0.5 phenotype points; revised MAF criteria and functional data	BP1, PM2_supp^b^	VUS
c.3950A>G (p.Glu1317Gly)	ClinVar: 1319598	VUS	BP1, PM2_supp	VUS	observed in 2 individuals worth 0 phenotype points	BP1, PM2_supp	VUS
c.4139C>T (p.Thr1380Ile)	ClinVar: 233890	VUS	BP1, PM2_supp, PS4_supp	VUS	observed in 9 individuals worth 3 phenotype points	BP1, PM2_supp, PS4_mod	VUS
c.4735A>T (p.Ile1579Phe)	ClinVar: 246402	VUS	BP1, PM2_supp, PS3_supp	VUS	observed in 4 individuals worth 1.5 phenotype points	BP1, PM2_supp, PS3_supp PS4_supp	VUS
c.5038C>T (p.Gln1680Ter)	ClinVar: 230520	likely pathogenic	PVS1, BS1	VUS	observed in 1 individual worth 0 phenotype points; revised MAF criteria	PVS1, PM2_supp^b^	likely pathogenic
c.6905C>G (p.Ser2302Ter)	ClinVar: 428166	pathogenic	PVS1	VUS	observed in 1 individual worth 0 phenotype points; revised MAF criteria	PVS1, PM2_supp^b^	likely pathogenic
c.7489_7490insT (p.Ser2497PhefsTer14)	ClinVar: 653103	pathogenic	PVS1, BS1	VUS	observed in 4 individuals worth 1 phenotype point; revised MAF criteria	PVS1, PS4_supp, PM2_supp^b^	pathogenic
c.7798_7801del (p.Gln2600ValfsTer15)	ClinVar: 827255	pathogenic	PVS1, BS1	VUS	observed in 2 individuals worth 0.5 phenotype points; observed in 3 individuals worth 3 healthy individual points; revised MAF criteria	PVS1, PM2_supp^b^, BS2_supp	likely pathogenic
c.7803_7807del (p.Ser2601ArgfsTer17)	ClinVar: 648862	likely pathogenic	PVS1	VUS	observed in 1 individual worth 0 phenotype points; revised MAF criteria	PVS1, PM2_supp^b^	likely pathogenic

a Database ID from the LOVD has the prefix of “APC_”; database ID from ClinVar are numbers only.

b Variants for which the reassessment of minor allele frequency (MAF) criteria where relevant for the final evaluation as LP/P.

c This variant seems to be a nonsense variant (p.Arg348Ter) located in exon 10, but RNA analysis (Ambry internal data) resulted in an in-frame aberrant transcript lacking part of exon 10 (r.934_1074del [p.Val312_Gln358del]) and increased expression of a naturally occurring alternative transcript (r.934_1236del [p.Val312_Gln412del], known as “exon 9a”) relative to controls. The predicted premature stop codon (p.Arg348Ter) was excluded from both the aberrant and naturally occurring transcripts and might provide a rescue mechanism for this nonsense alteration.

## Results

### Variant assessment using the reclassification algorithm based on *APC-*specific criteria

**Figure 4:**
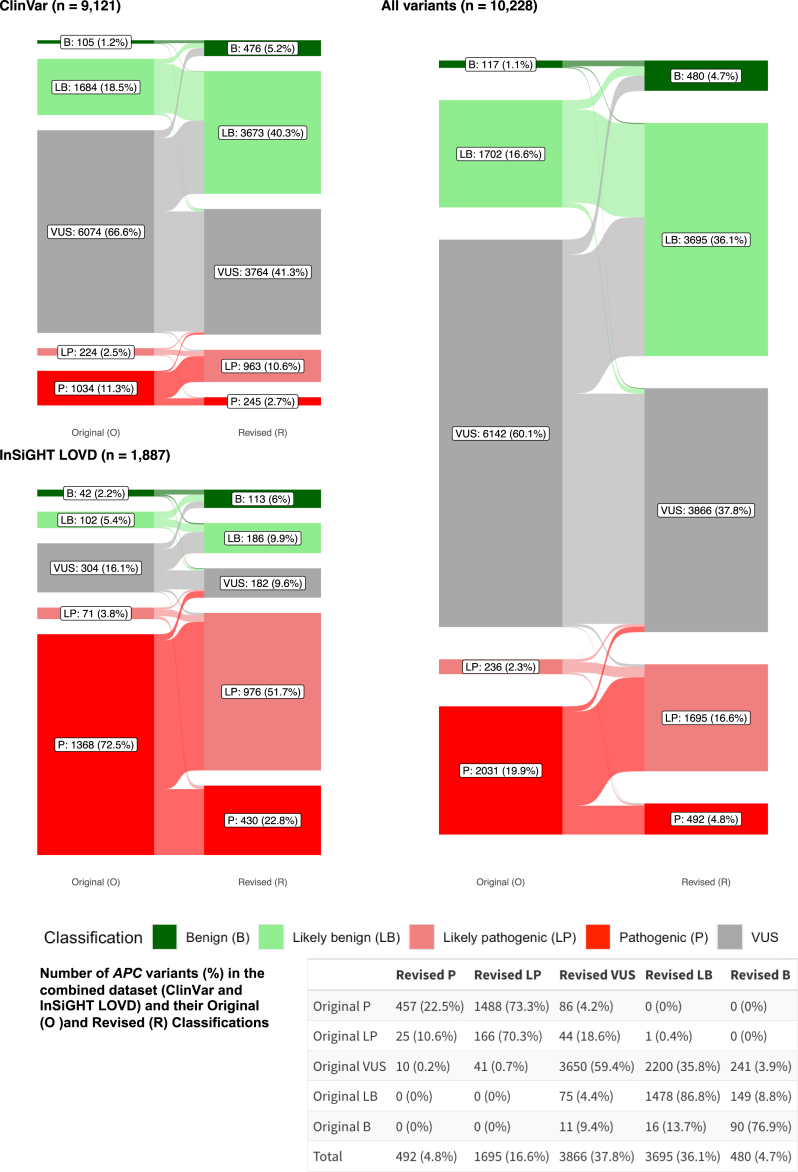
Classification of all *APC* variants in ClinVar, InSiGHT LOVD, and the combined dataset Each plot shows the classification change between the original (left) and revised classifications (right) for the *APC* variants in ClinVar (top left), InSiGHT LOVD (bottom left), and the combined dataset (right). The bottom table shows the number of *APC* variants (%) in the combined dataset (ClinVar and InSiGHT LOVD) and their original and revised classifications.

A total of 10,228 *APC* variants were analyzed in this study, which included 190 (2%) structural and 10,038 (98%) non-structural variants (Table S2). A total of 9,121 and 1,887 *APC* variants were present in ClinVar and LOVD, respectively (Figure 4); 780 (41% of LOVD) variants are shared between ClinVar and LOVD. The largest group in the ClinVar dataset is the VUS class (67%) followed by the B/LB group (20%) and P/LP group (14%). Most variants in LOVD are P/LP (76%), whereas VUS only account for 16% and B/LB variants for 8%.

Comparison between the original ClinVar or LOVD assertions and the revised classification are shown in Figures 4 and S1. By applying the *APC*-specific criteria, in the ClinVar dataset, now the largest group is the B/LB group (46%) followed by VUS (41%) and P/LP (13%). In the LOVD dataset, the majority of variants remained as P/LP (75%) followed by B/LB (16%) and VUS (10%). All the other reclassification results refer to the combined dataset in which the B/LB variants represent the largest group (41%) followed by VUS (38%) and P/LP (21%). Notably, 95% of variants with an initial classification of B/LB or P/LP remained in their respective benign and pathogenic categories after reclassification. A considerable portion of previous P variants were downgraded to LP (70%). Remarkably, 41% of previous VUSs were re-classified into clinically relevant pathogenicity classes (40% as B/LB and 1% as P/LP). On the other hand, 86 previously B/LB (4.7%) and 130 previously P/LP variants (5.7%) were reclassified as VUSs, which are summarized in Table S3. Therefore, the total number of VUSs was significantly reduced by 37% from 6,142 to 3,866 through the reclassification process (*p* value <0.05).

**Figure 5:**
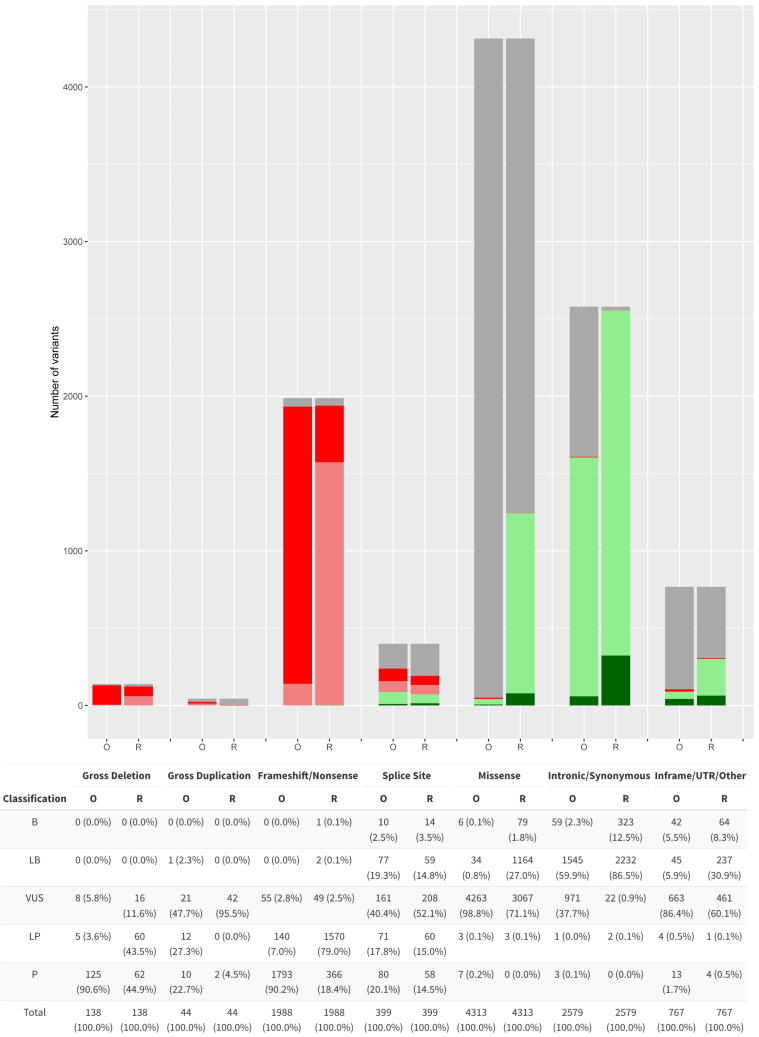
Classification of all *APC* variants in the original database (O) and their revised classification (R) by variant type Variants are broadly categorized into seven categories: 138 gross deletions, 44 gross duplications, 1,988 frameshift/nonsense, 399 splice site, 4,313 missense, 2,579 intronic/synonymous, and 767 other variants (120 in-frame, 631 UTR, and 16 other variants, which included start-loss, stop-loss, stop-retained, Alu and SVA retrotransposon insertions, inversions, and complex variants).

Classification by variant type is shown in Figure 5 with the majority being missense variants (42%) followed by synonymous or intronic variants (25%) and truncating (frameshift/nonsense) variants (19%). The original (O) and revised (R) pathogenicity class was compared for each variant type. In total, the percentage of frameshift/nonsense variants classified as P/LP is the same before and after reclassification (97%). In contrast, the application of the *APC*-specific criteria reduced the percentage of synonymous/intronic (at or beyond +7/−21 intronic positions) VUSs significantly from 38% to 1%, classifying the vast majority as B/LB (99%; *p* value <0.05). In the original assertions, 99% of putative missense variants are VUSs. This proportion was reduced significantly to 71% using the *APC*-specific criteria, where 29% of all missense variants were reclassified as B/LB (*p* value <0.05). For variants flanking splice sites (within +7/−21 intronic positions), the range of classifications was similar before and after reclassification. 39% of in-frame, UTR, and other variants were also classified as B/LB, while prior to reclassification they were mostly VUSs (86%). After application of the specific criteria, 3,866 variants remained VUSs, which included 3,067 missense variants (79%) and low numbers of other variant types.

### Impact and usage of the *APC*-specific codes and code combinations

The frequency of use of each *APC*-specific code is shown in Table S4A and the codes and code combinations with highest impact on VUS reclassification in Table S4B. All criteria were used at least once during the reclassification process. The most frequently applied codes were the two pathogenic criteria PM2_supporting (69%) and PVS1_variable (21%) and the four benign criteria BP1 (41%), BP4 (27%), BP7 (22%), and BS1 (20%). On the other hand, half of the codes are used for less than 1% of variants. 3,145 variants (31%) were present in the gnomAD v.2.1.1 non-cancer population and/or the UK Biobank non-CRC dataset, of which 2,021 (20%) could be classified as likely benign by the BS1 code alone and 427 (4%) as benign by the BA1 code alone, resulting in a significant reduction of VUSs (*p* values <0.05 for both). 155 variants (1.5%) were present in the non-CRC control dataset of UK Biobank, which fulfilled the definition for a healthy unaffected individual in the *APC*-specific criteria and allowed the assessment for BS2_variable. The most common code combination overall was BP1 and PM2_supporting resulting in a VUS classification (2,611, 26%). As shown in Table S4B, the most common code combination that led to a non-VUS classification was PVS1 and PM2_supporting, which were applied to 2,138 variants (21%). 80% of these variants (1,707; 17% of all variants) were classified as LP without the need of clinical information, and 20% (431; 4% of all variants) were classified as P together with variable other codes (e.g., PS4_variable). This was also the most efficient code combination to reclassify VUSs into a pathogenic (LP/P) class, although the number is small (0.7% reduction of all VUSs; *p* value <0.05) since only very few truncating variants were previously classified as VUSs.

As expected, the allele frequency threshold criterion BS1 was most frequently applied and has the highest impact on shifts from VUS into a (L)B classification (25% reduction of all VUSs; *p* value < 0.05). In addition, the codes BP4 and BP7 for intronic and synonymous variants without splice effect had a significant impact on VUS reduction (11% reduction of all VUSs; *p* value < 0.05).

### Further data mining and criteria review for a prioritized list of variants

After the initial application of the *APC*-specific criteria, a considerable fraction of VUSs (63%) remained as expected. We selected 37 promising variants from these remaining VUSs with some evidence for pathogenicity (for details, see methods), which formed a prioritized list of variants for further review as outlined in the workflow (Figure 3).

In 11 variants for which PVS1 is applicable (Table 1, encompassing both group 1 and 2), the relegation of their prior LP/P classification was due to their presence at very low frequencies in reference population databases, in this case the occurrence of one allele in a gnomAD non-cancer subpopulation. Depending on the denominator (i.e., size of the subpopulation), seven previously LP/P truncating variants were precluded from the use of PM2_supporting, and four even fulfilled threshold for BS1 using version 1.0.0 of the *APC*-specific criteria. To resolve this issue, the *APC* VCEP added a caveat to PM2_supporting in the criteria version 2.1.0 where the allele frequency threshold of ≤0.0003% (0.000003) is only used if the allele count is >1. To tolerate singleton allele occurrence in gnomAD, the *APC* VCEP set an allele frequency of <0.001% (0.00001) (lower than BS1) if the allele count is ≤1. Moreover, the *APC* VCEP recommended in criteria version 2.1.0 the use of the filtering allele frequency (FAF) for BA1 and BS1 to avoid the issue of singleton alleles satisfying the allele frequency criteria. This allowed the use of PM2_supporting and the reclassification of these 11 variants as LP/P.

For five variants that were originally P/LP in ClinVar or LOVD but reclassified as VUSs by the algorithm, a more extensive data mining and literature review led to the return of P/LP as their final classification (group 1). The previous pathogenic classification would indicate the observation of these variants in affected individuals on multiple occasions, and a targeted search was finally informative.

For the remaining 32 variants (group 2) further phenotype data was requested from VCEP members, given their persistent VUS status despite additional curation of the literature. This allowed the upgrade of classification from VUS to P/LP for 11 (34%) variants (group 2). Five variants of group 2 were evaluated as LP based on the reassessment of the MAF criteria, but no relevant phenotypic information was available. Overall, further data mining of selected representative variants resulted in the enhanced classification of 24 out of 37 variants (65%) into meaningful pathogenicity classes.

## Discussion

The rising number of VUSs in clinically actionable genes such as *APC* represents an important issue in the post-genomic era that hinders the translation of genetic diagnostics into clinical practice. In this study, we first identified the current landscape of *APC* variants in two of the most prominent international databases, namely ClinVar and the InSiGHT LOVD, and then applied the full set of ClinGen-approved, gene-specific ACMG/AMP criteria with the aim to improve consistency and accuracy in *APC* variant classification for >10,000 variants. A striking difference was noted in the distribution of pathogenicity classes: while around two-thirds of ClinVar variants were originally VUSs and 20% B/LB, approximately 75% of *APC* variants on LOVD were P/LP (Figure 4). This is not unexpected since variants submitted to LOVD are usually detected in individuals with the relevant phenotype (i.e., clinically evident colorectal adenomatous polyposis) where the detection of PVs is more likely. In contrast, variants in ClinVar are mostly derived from high-throughput sequencing approaches in individuals with less specific or unrelated phenotypes (e.g., multigene hereditary cancer panel testing in patients having testing for reasons other than a history of adenomatous polyposis) and healthy individuals. Consequently, the majority of these variants is expected to be benign or have a low penetrance. However, in the absence of overwhelming evidence, they are usually conservatively classified as VUSs.

One of the major findings of this study is that the application of the *APC*-specific criteria reduced the number of VUSs by 41% collectively in ClinVar and LOVD, the majority (40%) were reclassified as B/LB (*n* = 2,441) owing to their presence in reference population databases fulfilling MAF criteria (BA1 or BS1) or no predicted impact on splicing (BP4 + BP7). Their benign classification in turn alleviates anxiety and potential overtreatment in affected individuals worldwide.^60^ On the other hand, 51 previous VUSs were reclassified as P/LP (0.8%), a result which confirms the diagnosis of FAP and enables the timely management and predictive testing of all at-risk relatives. Finally, 95% and 94% of the previously B/LB and P/LP variants remained in their concordant classifications. These findings demonstrate that the application of the *APC*-specific criteria is highly effective in improving the reclassification of VUSs into clinically relevant pathogenicity classes while preserving the original interpretation of variants with existing evidence-based classifications.

In the combined databases, a total of 130 P/LP variants (93 non-structural, 37 structural) and 86 B/LB variants (85 structural, 1 non-structural) were reclassified as VUSs (216 in total, 5% of previous non-VUSs), which is clinically significant for the previous P/LP variants and will affect the diagnosis, counseling, and predictive testing (Table S3). Among the 130 P/LP-to-VUS variants, 14 were gross deletions, affecting only the promoter region and/or the first coding exon, and 21 gross duplications which had unknown impact on the reading frame, even though many *APC* gross duplications are indeed located in a tandem position.^4^^,^^61^^,^^62^ In the absence of convincing clinical data, these gross deletions and duplications could not be classified as LP/P. Sixteen truncating variants at the 5′ or 3′ end of the gene were excluded from the application of PVS1 and were classified as VUSs. We also noted that canonical ±1/2 splice site changes and intronic variants flanking the splice sites (+7/−21 bp) were mostly deemed LP/P by LOVD and ClinVar submitters (43/62 variants [69%] that were reclassified as VUSs), although splice predictions and transcript analyses might have suggested otherwise (i.e., weak native site, alternative transcripts, etc.). On the other hand, RNA analysis data supporting a splice effect were mainly not available for this large-scale project. The remaining variants included 55 deep intronic, synonymous, in-frame, and UTR variants, as well as 25 missense variants, where the reclassification as VUS was the appropriate result of a combination of scarcity of clinical data and non-contributory *in silico* predictions.

In the *APC*-specific criteria, a range of evidence weight adjustments is specified as a means of improving precision and quality. The lack of detailed evidence descriptions in databases and publications meant that certain criteria can only be applied at lower weights, even though ClinVar and LOVD submitters may have additional information, especially the phenotype (including segregation) and functional analyses. Since this information is either not publicly available in the databases or is elaborate to extract from publications, the clinical-data-driven criteria are only applied at the end of the reclassification algorithm (Figure 2). In this study, clinical data were only extracted from individuals with phenotype data in LOVD. For a selected number of promising variants (*n* = 37; Table 1), additional data mining and a request among our VCEP members for clinical and if necessary for RNA analysis data was carried out. 65% of these variants could again be reclassified into P/LP, demonstrating the relevance of further clinical and RNA analysis data.

A considerable number of previous P variants were downgraded to LP (70%) (e.g., truncating variants fulfilling only PVS1 and PM2_supporting)—a result also known from other genes where gene-specific rules are being applied. Submitters may be overrating the quality of phenotype data, have additional data, or were applying the original moderate strength for PM2. Consequently, it seems likely that some reclassified LP variants are in fact P, which has the potential for upgrade through diligent reporting of clinical information and data sharing. In practical terms, an LP classification has a posterior probability of pathogenicity of 0.9–0.99, which nonetheless demands clinical action when detected.^63^^,^^64^

After reclassification, 80% of the remaining VUSs (3,067/3,866) were presumed missense variants, which don’t meet the allele frequency thresholds for BA1 and BS1. As discussed previously,^24^^,^^34^ true LP/P missense variants are extremely rare in *APC* since the fundamental mechanism of *APC* pathogenicity is based on the loss of a large C-terminal part of the protein which includes the relevant functional domains. In addition to further evidence of functional redundancy in the APC protein, the central and C-terminal domains of the APC protein are natively unfolded, which likely explains its resistance to missense variation.^65^ Consequently, the vast majority of the remaining missense *APC* VUSs in ClinVar are presumably benign incidental findings from non-targeted testing in individuals with unrelated phenotypes, although this is challenging to verify since *in silico* prediction tools are not applicable for *APC* missense variants.^24^ Similarly, massively parallel functional assays on protein function are unlikely to contribute significantly to improve classifications. Interpreting a missense *APC* variant as benign is therefore heavily dependent on the clinical description of the associated individual (a lack of CRC/polyposis phenotype), in which case BS2_supporting could be applied in conjunction with BP1 to result in an LB classification. The recruitment of variant data from large reference population projects such as the UK Biobank is another option to determine the pathogenicity of these missense variants.

This reclassification endeavor was also considered a proof-of-concept study for the ongoing method of operation of VCEPs (Figure 3). The review and discussion of every single submitted constitutional variant in *APC* is unrealistic with respect to the resources currently provided to a VCEP until appropriate bioinformatic tools become available in the future. In this study, we developed a stratified variant curation process whereby the relatively straightforward variants could be processed in batch and become candidates for fast-track VCEP review (e.g., variants fulfilling BA1 or BS1 plus BP1 without any conflicting classifications submitted to ClinVar or LOVD) in an updated variant curation interface (VCI) to streamline the variant approval process. We identified several prioritized groups of variants that can be the subject of further targeted literature review (Table S3), data-mining, and clinical data requests from database submitters and VCEP members, a process to enhance variant interpretation as demonstrated in this study. These challenging variants can also form the basis for scientific follow-up studies to evaluate the causal relationships using additional investigations such as segregation or transcript analyses.

Variants that are listed as “conflicting” in ClinVar require special consideration by the VCEPs. However, there are only few *APC* variants in ClinVar with clinically significant conflict (defined as variant with concomitant VUS and P/LP classifications), including variants with different interpretations of splicing effects, truncating variants upstream or downstream of the PVS1 boundaries and few missense variants. Some of these variants have been already assessed by the VCEP or are prioritized for further expert curation and panel review. As per the ClinVar assertion process, variants fully classified by the VCEP will appear with a three-star pathogenicity assertion that overrules the other submissions and therefore will no longer be conflicting. A special case are the two widely known low-penetrant variants c.3920T>A (p.Ile1307Lys) and c.3949G>C (p.Glu1317Gln), which are listed as conflicting in ClinVar. As the ACMG/AMP rules were designed for a Mendelian inheritance model, they cannot be applied to such variants.^24^ The current evidence of the p.Ile307Lys variant was recently summarized,^66^ and the ClinGen Low Penetrance/Risk Allele Working Group has published recommendations for how these variants should be reported.^67^

In this study, we developed and applied an algorithm for large-scale variant reclassification and demonstrated that the application of *APC*-specific criteria can substantially alleviate the burden of VUSs in ClinVar and LOVD, thereby laying the groundwork for a prospective streamlined expert panel approval of clinically actionable *APC* variants in the VCI. By using the VCEP specifications, diagnostic laboratories could reduce their rates of reporting VUSs. Previous studies for other genes were either smaller, used gene- or disease-specifications that covered only partial evidence domains,^68^ or meta-classification methods such as the multifactorial likelihood analysis.^69^^,^^70^ The present study highlights the utility of a systematic, data-driven analysis using gene-specific ACMG/AMP criteria, complemented by further targeted data-mining and clinical data requests. By this approach, this study marks the initiation of a dynamic, long-term curation process for the *APC* VCEP. The suggested workflow can serve as a generalizable model of operation for other gene- or disease-specific variant interpretation initiatives, achieving accurate and highly efficient variant interpretation based on an array of carefully curated evidence.

To further improve VUS interpretation and provide clinically informative variant classification beyond this approach, the availability of more population-based datasets and user-friendly modes of sharing clinical and molecular data are needed; a challenge that needs to be addressed by the respective expert communities and data submitters.

## Data and code availability

All data supporting the findings and conclusions and all significant results generated during this study are available within the published article and the supplemental information. All variants reviewed and reclassified by the ClinGen-InSiGHT Variant Curation Expert Panel in this study have been submitted to the ClinVar Database. The detailed evidence used for the classification of these variants is available in the ClinGen Evidence Repository (https://erepo.clinicalgenome.org/evrepo/). These data and all internal data cited in this manuscript may also become available upon a data transfer agreement approved by the local ethics committee and can be obtained after contacting the corresponding author (S.A.) upon request.

## Acknowledgments

This publication was supported in part by the 10.13039/100000051National Human Genome Research Institute of the National Institutes of Health for the Baylor College of Medicine/Stanford University Clinical Genome Resource-2U24HG009649 and from the 10.13039/100000054National Cancer Institute U24 Curation Panels through U24CA258119. This work was supported (not financially) by the European Reference Network on Genetic Tumour Risk Syndromes (ERN GENTURIS): project ID no. 739547. ERN GENTURIS is partly co-funded by the 10.13039/501100000780European Union within the framework of the Third Health Programme “ERN-2016—Framework Partnership Agreement 2017–2021.” We thank John Ranola for his support and guidance in statistical analyses.

## Declaration of interests

S.E.P. is a member of the scientific advisory panel of Baylor Genetics Laboratories.

## Web resources

ClinGen (Clinical Genome Resource), www.clinicalgenome.org
ClinGen-InSiGHT Hereditary Colorectal Cancer/Polyposis Variant Curation Expert Panel, https://www.clinicalgenome.org/affiliation/50099/
Criteria Specification Registry, https://cspec.genome.network/cspec/ui/svi/
ClinVar, https://www.ncbi.nlm.nih.gov/clinvar/
Ensembl Variant Effect Predictor, https://asia.ensembl.org/info/docs/tools/vep/index.html
Global Variome shared Leiden Open Variation Database (LOVD) https://www.lovd.nl/apc; https://databases.lovd.nl/shared/genes/APC
HGVS (Human Genome Variation Society), https://varnomen.hgvs.org/
InSiGHT (International Society for Gastrointestinal Hereditary Tumours), https://www.insight-group.org/
InSiGHT *APC* LOVD, https://www.insight-database.org/genes/APC
MaxEntScan for 5′ sites, http://hollywood.mit.edu/burgelab/maxent/Xmaxentscan_scoreseq.html
MaxEntScan for 3′ sites, http://hollywood.mit.edu/burgelab/maxent/Xmaxentscan_scoreseq_acc.html
OMIM (Online Mendelian Inheritance in Man), https://www.omim.org
SpliceAI, https://spliceailookup.broadinstitute.org/

## Supplemental information

Document S1. Figure S1 and Tables S1, S3, and S4

Table S2. Final classification of 10,228 *APC* variants in ClinVar and InSiGHT LOVD

Document S2. Article plus supplemental information
