# Supplementary material for: Large-scale application of ClinGen-InSiGHT APC-specific ACMG/AMP variant classification criteria leads to substantial reduction in VUS
Source: Am J Hum Genet. 2024 Oct 1;111(11):2427–43. doi: 10.1016/j.ajhg.2024.09.002 (PMC11568752; doi:10.1016/j.ajhg.2024.09.002)
Supplement: Document S1. Figure S1 and Tables S1, S3, and S4 [file mmc1.pdf]

The American Journal of Human Genetics, Volume *111*

## Supplemental information

# Large-scale application of ClinGen-InSiGHT *APC*-specific ACMG/AMP variant classification criteria leads to substantial reduction in VUS

**Xiaoyu Yin, Marcy Richardson, Andreas Laner, Xuemei Shi, Elisabet Ognedal, Valeria Vasta, Thomas v.O. Hansen, Marta Pineda, Deborah Ritter, Johan de Dunnen, Emadeldin Hassanin, Wencong Lyman Lin, Ester Borras, Karl Krahn, Margareta Nordling, Alexandra Martins, Khalid Mahmood, Emily Nadeau, Victoria Beshay, Carli Tops, Maurizio Genuardi, Tina Pesaran, Ian M. Frayling, Gabriel Capellá, Andrew Latchford, Sean V. Tavtigian, Carlo Maj, Sharon E. Plon, Marc S. Greenblatt, Finlay A. Macrae, Isabel Spier, and Stefan Aretz**

**Figure S1 (A)** Number of *APC* variants (%) in the ClinVar and their original and revised classifications

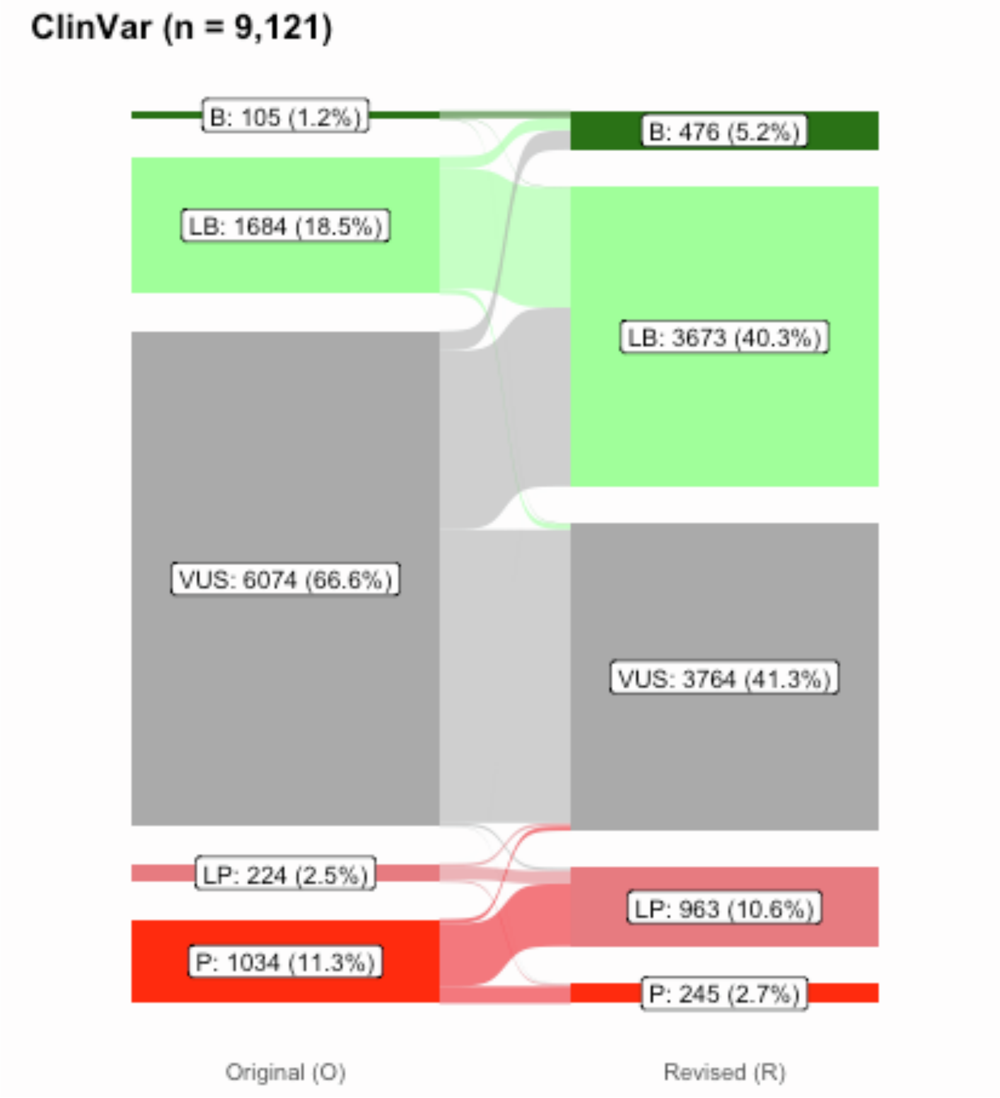


| | Revised P | Revised LP | Revised VUS | Revised LB | Revised B |
|---|---|---|---|---|---|
| Original P | 212 (20.5%) | 768 (74.3%) | 54 (5.2%) | 0 (0%) | 0 (0%) |
| Original LP | 24 (10.7%) | 159 (71%) | 40 (17.9%) | 1 (0.4%) | 0 (0%) |
| Original VUS | 9 (0.1%) | 36 (0.6%) | 3597 (59.2%) | 2193 (36.1%) | 239 (3.9%) |
| Original LB | 0 (0%) | 0 (0%) | 66 (3.9%) | 1471 (87.4%) | 147 (8.7%) |
| Original B | 0 (0%) | 0 (0%) | 7 (6.7%) | 8 (7.6%) | 90 (85.7%) |
| Total | 245 (2.7%) | 963 (10.6%) | 3764 (41.3%) | 3673 (40.3%) | 476 (5.2%) |

**(B)** Number of *APC* variants (%) in the InSiGHT LOVD and their original and revised classifications

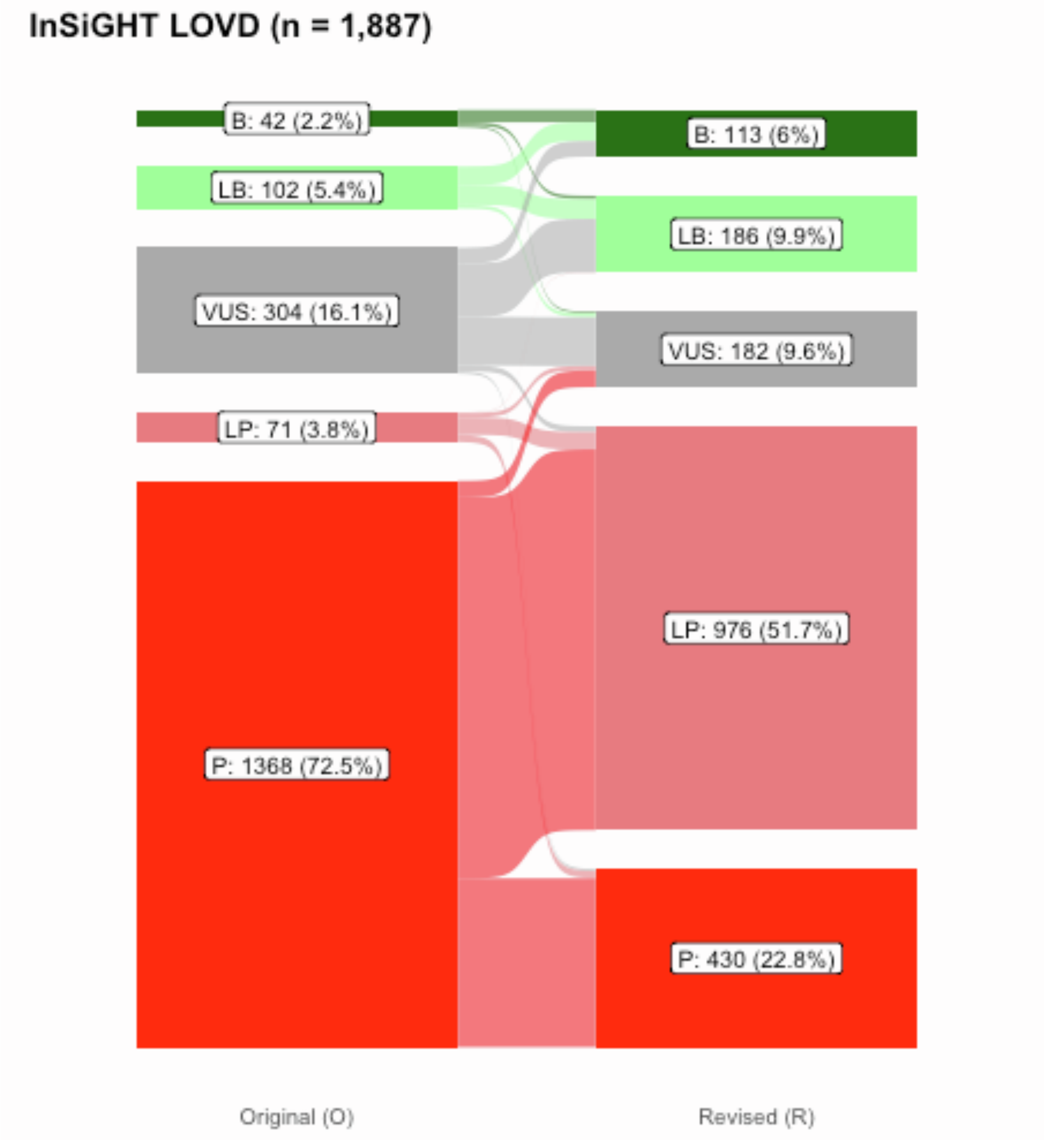


| | Revised P | Revised LP | Revised VUS | Revised LB | Revised B |
|---|---|---|---|---|---|
| Original P | 408 (29.8%) | 920 (67.3%) | 40 (2.9%) | 0 (0%) | 0 (0%) |
| Original LP | 17 (23.9%) | 42 (59.2%) | 11 (15.5%) | 1 (1.4%) | 0 (0%) |
| Original VUS | 5 (1.6%) | 14 (4.6%) | 118 (38.8%) | 129 (42.4%) | 38 (12.5%) |
| Original LB | 0 (0%) | 0 (0%) | 9 (8.8%) | 48 (47.1%) | 45 (44.1%) |
| Original B | 0 (0%) | 0 (0%) | 4 (9.5%) | 8 (19%) | 30 (71.4%) |
| Total | 430 (22.8%) | 976 (51.7%) | 182 (9.6%) | 186 (9.9%) | 113 (6%) |

**Table S1 Status of ClinVar and all *APC* variant databases**

| *Database name & URL* | Curation & submission status | Date of Access | Submissions | Unique variant | Status |
|---|---|---|---|---|---|
| ClinVar https://www.ncbi.nlm.nih.gov/clinvar/?term=APC[gene] | Not curated, accepting submissions | 21 March 2022 | 18029 | 9121 | Active |
| Global Variome shared LOVD https://www.lovd.nl/apc; https://databases.lovd.nl/shared/genes/APC | Curated, accepting submissions | 12 May 2022 | 5663 | 1877 | Active |
| InSiGHT *APC* LOVD http://www.insight-database.org/genes/APC | Merged with Global Variome shared LOVD | 12 May 2022 | 5663 | 1877 | Active |
| The UMD *APC* mutations database http://www.umd.be/APC/ | Curated, not accepting submissions | 21 July 2022 | 3717 | 720 | Active |
| Argentina National Institute of Cancer http://www.inc.gob.ar/sither/genes/APC | Curated, accepting submissions | 21 July 2022 | 161 | 48 | Active |
| Brazilian initiative on Precision Medicine<br>http://bipmed.iqm.unicamp.br/genes/APC<br>http://bipmed.iqm.unicamp.br/snparray/genes/APC<br>http://bipmed.iqm.unicamp.br/snparray_hg19/genes/APC<br>http://bipmed.iqm.unicamp.br/snparray_296/genes/APC<br>http://bipmed.iqm.unicamp.br/wes_hg19/genes/APC | Not curated, not accepting submissions | 21 July 2022 | <br>54<br>23<br>23<br>23<br>74 | <br>54<br>23<br>23<br>23<br>74 | Active |
| The *APC* mutation database http://fap.taenzer.me/ | Inactive, URL not found | | | | Inactive |
| Canadian Open Genetics Repository http://opengenetics.ca/#/brca/gene/APC | Curated, not accepting submissions | 21 July 2022 | | 259 | Active |
| CanVas – A Greek Cancer Patient Genetic Variation Resource http://ithaka.rrp.demokritos.gr/CanVaS/genes/APC | Curated, accepting submissions | 21 July 2022 | 593 | 177 | Active |
| The Cyprus *APC* LOVD http://db.cshg.org.cy/genes/APC | Inactive, URL not found | | | | Inactive |
| Dian Diagnostics & Zhejiang University Center for Genetic and Genomic Medicine http://www.genomed.org/lovd2/home.php?select_db=APC | Installation lost | | | | Inactive |
| Iran Variation Database http://genet.ir/variome/genes/APC | Contained hidden *APC* variants only; LOVD installation partially broken | | | | Inactive |
| Malaysian Node of the Human Variome Project Database http://www.kk.usm.my/LOVDv.3.0/genes/APC | Curated, accepting submissions | 21 July 2022 | 35 | 29 | Active |
| MexVar https://bipmed.fcm.unicamp.br/mexvar/genes/APC | Inactive, URL not found | | | | Inactive |
| Nicaragua APC http://databases.lovd.ni/shared/genes/APC | Inactive, URL not found | | | | Inactive |
| Spain MDB https://lovd3.isciii.es/genes/APC | Did not contain any *APC* variant record | | | | Inactive |
| Zhejiang University-Adinovo Center APC Database http://databases.lovd.nl/genomed/home.php?select_db=APC | Inactive, URL not found | | | | Inactive |
| Other LOVD installation – LOVD3 whole genome datasets http://databases.lovd.nl/whole_genome/ | Not curated; not accepting submissions; imported from Exome Variant Server | 21 July 2022 | 321 | 321 | Active |
| Other LOVD installation – by the University of Melbourne http://proteomics.bio21.unimelb.edu.au/lovd/genes/APC | Not curated; inactive, not accepting submissions | | | | Inactive |
| | | | | | |

# Table S3 Variants reclassified as VUS from clinically relevant classifications (B/LB/P/LP)

***Deletion at the extremities of the gene with unclear molecular consequences on the protein structure***

| Database_ID | HGVSc; HGVSp | Predicted consequence | Prior classification | Reclassification by APC-specific criteria |
|---|---|---|---|---|
| ClinVar 495348 | NM_000038.5:c.(?_-37541)_(-27791_?)del | deletion: promoter 1B | **Pathogenic** | VUS: PM2_supporting |
| ClinVar 832064 | NC_000005.10:g.(?_112707312)_(112755035_?)del | deletion: promoter 1B and 1A, exons 2 | **Pathogenic** | VUS: PM2_supporting |
| LOVD APC_001246 | NM_001127511.3:c.165+17816_166-18738delinsTGCTCTATGACCAATT | deletion: promoter 1B deletion | **Pathogenic** | VUS: PM2_supporting |
| ClinVar 580918 | NC_000005.9:g.112001178_112043328del42151 | deletion: promoter 1B partial | Likely pathogenic | VUS: PM2_supporting |
| ClinVar 433562 | NM_000038.5:c.-85-?_-19+?del | deletion: exon 1 | **Pathogenic** | VUS: PM2_supporting |
| LOVD APC_000526 | NM_000038.6:c.-85_(135+1_136-1) | deletion: exon 1-2 | **Pathogenic** | VUS: PS4_supporting, PM2_supporting |
| ClinVar 1049778 | NM_000038.6:c.-2_135+1824del | deletion: exon 2 | **Pathogenic** | VUS: PM2_supporting |
| ClinVar 1049324 | NM_000038.6:c.-2_135+1274del | deletion: exon 2 | **Pathogenic** | VUS: PM2_supporting |
| ClinVar 1049076 | NM_000038.6:c.-2_136-2903del | deletion: exon 2 | **Pathogenic** | VUS: PM2_supporting |
| ClinVar 584665 | NM_000038.6:c.1895_1958+28del | deletion: exon 15 partial | **Pathogenic** | VUS: PM2_supporting |
| ClinVar 433572 | NM_000038.5:c.3146-?_8532+?del | deletion: exon 16 partial | **Pathogenic** | VUS: PM2_supporting |
| ClinVar 433571 | NM_000038.5:c.2155-?_3960+?del | deletion: exon 16 partial | **Pathogenic** | VUS: PM2_supporting |
| ClinVar 1071358 | NC_000005.9:g.(?_112174702)_112203173del | deletion: exon 16 partial | **Pathogenic** | VUS: PM2_supporting |
| ClinVar 1071357 | NC_000005.9:g.(?_112173930)_112310702del | deletion: exon 16 partial | **Pathogenic** | VUS: PM2_supporting |

***Large duplication and complex variants with unknown impact on the reading frame***

| Database_ID | HGVSc; HGVSp | Predicted consequence | Prior classification | Reclassification by APC-specific criteria |
|---|---|---|---|---|
| ClinVar 1067201 | NC_000005.9:g.(?_112072721)_(112090732_?)dup | duplication: promoter 1A, exon 2 | **Likely pathogenic** | VUS: PM2_supporting |
| ClinVar 584349 | NC_000005.9:g.(?_112072721)_(112090728_?)dup | duplication: promoter 1A, exon 2 | **Likely pathogenic** | VUS: PM2_supporting |
| ClinVar 469686 | NC_000005.9:g.(?_112072721)_(112111440_?)dup | duplication: promoter 1A, exons 2-5 | **Likely pathogenic** | VUS: PM2_supporting |
| ClinVar 1067204 | NC_000005.9:g.(?_112071797)_112137006dup | duplication: promoter 1A, exons 2-7, exon 8 partial | **Likely pathogenic** | VUS: PM2_supporting |
| ClinVar 1067082 | NC_000005.9:g.(?_112090582)_(112157694_?)dup | duplication: exon 1-11 | **Likely pathogenic** | VUS: PM2_supporting |
| ClinVar 58098 | GRCh38/hg38 5q15-22.3(chr5:96454445-114050905)x3 | duplication: exon 1-16 | **Pathogenic** | VUS: PM2_supporting |
| ClinVar 394550 | GRCh37/hg19 5q21.3-35.3(chr5:106716357-180687338)x3 | duplication: exon 1-16 | **Pathogenic** | VUS: PM2_supporting |
| ClinVar 425542 | GRCh37/hg19 5q15-35.3(chr5:94844077-178830410)x3 | duplication: exon 1-16 | Likely benign | VUS: PM2_supporting |
| ClinVar 688598 | GRCh37/hg19 5q14.3-23.3(chr5:89949118-129317455)x3 | duplication: exon 1-16 | **Pathogenic** | VUS: PM2_supporting |
| ClinVar 607687 | GRCh37/hg19 5p15.33-q35.3(chr5:25328-180693344)x3 | duplication: exon 1-16 | **Pathogenic** | VUS: PM2_supporting |
| ClinVar 607681 | GRCh37/hg19 5p15.33-q35.3(chr5:13648-180905029)x3 | duplication: exon 1-16 | **Pathogenic** | VUS: PM2_supporting |
| ClinVar 441919 | GRCh37/hg19 5p15.33-q35.3(chr5:113577-180719789)x3 | duplication: exon 1-16 | **Pathogenic** | VUS: PM2_supporting |
| ClinVar 441920 | GRCh37/hg19 5p15.33-q35.3(chr5:113577-180719789) | duplication: exon 1-16 | **Pathogenic** | VUS: PM2_supporting |
| ClinVar 417556 | NC_000005.9:g.(?_112090570)_(112157688_?)dup | duplication: exon 2-11 | **Likely pathogenic** | VUS: PVS1_strong, PM2_supporting |
| ClinVar 469688 | Single allele | duplication: exon 2-4 | **Likely pathogenic** | VUS: PVS1_strong, PM2_supporting |
| ClinVar 830510 | NC_000005.10:g.(?_112754891)_(112767400_?)dup | duplication: exons 2-4 | **Likely pathogenic** | VUS: PVS1_strong, PM2_supporting |
| ClinVar 1067200 | NC_000005.9:g.(?_112090582)_(112137086_?)dup | duplication: exon 2-8 | **Likely pathogenic** | VUS: PVS1_strong, PM2_supporting |
| LOVD APC_001753 | NM_000038.6:c.(135+1_136-1)_(422+1_423-1)dup | duplication: exon 3-4 | **Pathogenic** | VUS: PVS1_strong, PM2_supporting |
| LOVD APC_000750 | NM_000038.6:c.(422+1_423-1)_(531+1_532-1)dup | duplication: exon 4-5 | **Pathogenic** | VUS: PVS1_strong, PM2_supporting |
| LOVD APC_001782 | NM_000038.6:c.(834+1_835-1)_(1408+1_1409-1)dup | duplication: exon9-11 | **Likely pathogenic** | VUS: PVS1_strong, PM2_supporting |
| ClinVar 1067081 | NC_000005.9:g.(?_112170638)_(112170872_?)dup | duplication: exon 15 | **Likely pathogenic** | VUS: PVS1_strong, PM2_supporting |
| LOVD APC_001415 | NM_000038.6:c.1806_1817delinsN[300] | other | **Pathogenic** | VUS: PM2_supporting |
| LOVD APC_001953 | complex 3.9mb rearrangement | other | **Likely pathogenic** | VUS: PS4_supporting, PM2_supporting |
| ClinVar 243004 | NM_001127511.2:c.[-125delA;-195A>C] | other | **Pathogenic** | VUS: PM2_supporting |

***Variants at the 5' end of the gene and therefore excluded from the application of PVS1***

| Database_ID | HGVSc; HGVSp | Predicted consequences | Prior classification | Reclassification by APC-specific criteria |
|---|---|---|---|---|
| ClinVar 243007 | NM_001127511.3:c.-192A>T | UTR | **Pathogenic** | VUS: BP4, PM2_supporting |
| ClinVar 243006 | NM_001127511.3:c.-192A>G | UTR | **Likely pathogenic** | VUS: BS1 |
| ClinVar 652807 | NM_001127511.3:c.-192_-191delinsTAGCAAGGG | UTR | **Likely pathogenic** | VUS: PM2_supporting |
| ClinVar 243005 | NM_001127511.3:c.-191T>C | UTR | **Pathogenic** | VUS: BP4, PM2_supporting |
| LOVD APC_001802 | NM_000038.6:c.-190G>A | UTR | **pathogenic** | VUS: PM2_supporting |
| ClinVar 1050412 | NM_001127511.3:c.166-28469_166-27547del | UTR | **Pathogenic** | VUS: PM2_supporting |
| ClinVar 1050584 | NM_001127511.3:c.166-28467del | UTR | **Pathogenic** | VUS: BP4, PM2_supporting |
| ClinVar 537477 | NM_000038.6:c.14del; NP_000029.2:p.Ser5TyrfsTer6 | frameshift | **Pathogenic** | VUS: PM2_supporting |
| ClinVar 630969 | NM_000038.6:c.26_27insTTTA; NP_000029.2:p.Leu9PhefsTer7 | frameshift | **Pathogenic** | VUS: PM2_supporting |
| ClinVar 628229 | NM_000038.6:c.32_33insA; NP_000029.2:p.Gln12AlafsTer3 | frameshift | **Pathogenic** | VUS : PM2_supporting |
| ClinVar 970282 | NM_000038.6:c.55G>T; NP_000029.2:p.Glu19Ter | nonsense | **Pathogenic** | VUS: PM2_supporting |
| ClinVar 470090 | NM_000038.6:c.74_75del; NP_000029.2:p.Gln25ArgfsTer5 | frameshift | **Pathogenic** | VUS: PM2_supporting |
| ClinVar 428113 | NM_000038.6:c.93del; NP_000029.2:p.Asn32IlefsTer13 | frameshift | **Pathogenic** | VUS: PM2_supporting |
| ClinVar, LOVD 428154 | NM_000038.6:c.104del; NP_000029.2:p.Thr35LysfsTer10 | frameshift | **Pathogenic** | VUS: PM2_supporting |
| ClinVar 537502 | NM_000038.6:c.108del; NP_000029.2:p.Lys36AsnfsTer9 | frameshift | **Pathogenic** | VUS: PM2_supporting |
| ClinVar 579756 | NM_000038.6:c.132dup; NP_000029.2:p.Lys45GlufsTer5 | frameshift | **Pathogenic** | VUS: PM2_supporting |

***Variants that satisfied PVS1, however were present at very low frequencies in population reference database (PM2_supporting not met)***

| Database_ID | HGVSc; HGVSp | Predicted consequences | Prior classification | Reclassification by APC-specific criteria |
|---|---|---|---|---|
| ClinVar 654864 | NM_000038.6:c.156del; NP_000029.2:p.Gly53GlufsTer17 | frameshift | **Pathogenic** | VUS: PVS1 |
| ClinVar, LOVD 934724 | NM_000038.6:c.203del; NP_000029.2:p.Leu68TyrfsTer2 | frameshift | **Pathogenic** | VUS: PVS1 |
| ClinVar, LOVD 411479 | NM_000038.6:c.471G>A; NP_000029.2:p.Trp157Ter | nonsense | **Pathogenic** | VUS: PVS1 |
| ClinVar, LOVD 955439 | NM_000038.6:c.1042C>T; NP_000029.2:p.Arg348Ter | nonsense | **Pathogenic** | VUS: PVS1 |
| ClinVar 438865 | NM_000038.6:c.1333C>T; NP_000029.2:p.Gln445Ter | nonsense | **Pathogenic** | VUS: PVS1, BS1 |
| ClinVar, LOVD 183857 | NM_000038.6:c.4669_4670del; NP_000029.2:p.Ile1557Ter | frameshift | **Pathogenic** | VUS: PVS1 |
| ClinVar, LOVD 230520 | NM_000038.6:c.5038C>T; NP_000029.2:p.Gln1680Ter | nonsense | **Likely pathogenic** | VUS: PVS1, BS1 |
| ClinVar 428166 | NM_000038.6:c.6905C>G; NP_000029.2:p.Ser2302Ter | nonsense | **Pathogenic** | VUS: PVS1 |
| ClinVar 653103 | NM_000038.6:c.7489_7490insT; NP_000029.2:p.Ser2497PhefsTer14 | frameshift | **Pathogenic** | VUS: PVS1, BS1 |
| ClinVar 827255 | NM_000038.6:c.7798_7801del; NP_000029.2:p.Gln2600ValfsTer15 | frameshift | **Pathogenic** | VUS: PVS1, BS1 |
| ClinVar 648862 | NM_000038.6:c.7803_7807del; NP_000029.2:p.Ser2601ArgfsTer17 | frameshift | **Likely pathogenic** | VUS: PVS1 |

***Missense variants that are unable to be classified, mainly because minor allele frequency thresholds for BA1/BS1 are not met and lack of additional information***

| Database ID | HGVSc; HGVSp | Predicted consequences | Prior classification | Reclassification by APC-specific criteria |
|---|---|---|---|---|
| LOVD APC_001042 | NM_000038.6:c.446A>T; NP_000029.2:p.Asp149Val | missense | **pathogenic** | VUS: BP1, PM2_supporting, PS4_supporting |
| LOVD APC_000758 | NM_000038.6:c.623A>G; NP_000029.2:p.Gln208Arg | missense | **pathogenic** | VUS: BP1, PM2_supporting, PP1 |
| LOVD APC_000627 | NM_000038.6:c.1060C>T; NP_000029.2:p.Pro354Ser | missense | **pathogenic** | VUS: BP1, PM2_supporting |
| ClinVar 231954 | NM_000038.6:c.1902T>G; NP_000029.2:p.Ser634Arg | missense | **Likely pathogenic** | VUS: BP1, PS3_moderate |
| ClinVar 428167 | NM_000038.6:c.3077A>C; NP_000029.2:p.Asn1026Thr | missense | **Likely pathogenic** | VUS : PM2_supporting, PM5_supporting |
| ClinVar 802 | NM_000038.6:c.3359G>A; NP_000029.2:p.Gly1120Glu | missense | **Pathogenic** | VUS: BP1, PM2_supporting |
| ClinVar 817 | NM_000038.6:c.4183A>T; NP_000029.2:p.Ser1395Cys | missense | **Pathogenic** | VUS: BP1, PM2_supporting |
| LOVD APC_000208 | NM_000038.6:c.4549C>G; NP_000029.2:p.Gln1517Glu | missense | **pathogenic** | VUS: BP1, PM2_supporting, PS4_supporting |
| LOVD APC_001630 | NM_000038.6:c.6257C>A; NP_000029.2:p.Pro2086Gln | missense | **Likely pathogenic** | VUS: BP1, PM2_supporting |
| ClinVar 486771 | NM_000038.6:c.743A>G; NP_000029.2:p.Asn248Ser | missense | Likely benign | VUS: BP1 |
| ClinVar 827113 | NM_000038.6:c.754A>G; NP_000029.2:p.Thr252Ala | missense | Likely benign | VUS : BP1, PM2_supporting |
| ClinVar 487015 | NM_000038.6:c.2581G>A; NP_000029.2:p.Gly861Ser | missense | Likely benign | VUS: BP1, PM2_supporting |
| LOVD APC_001944 | NM_000038.6:c.2605A>G; NP_000029.2:p.Asn869Asp | missense | Likely benign | VUS: BP1, PM2_supporting |
| ClinVar 617980 | NM_000038.6:c.2651C>T; NP_000029.2:p.Ala884Val | missense | Likely benign | VUS: BP1, PM2_supporting |
| ClinVar 428175 | NM_000038.6:c.2909G>C; NP_000029.2:p.Ser970Thr | missense | Likely benign | VUS: BP1, PM2_supporting |
| LOVD APC_001667 | NM_000038.6:c.3290A>G; NP_000029.2:p.Glu1097Gly | missense | Likely benign | VUS: BP1, PM2_supporting |
| ClinVar 482305 | NM_000038.6:c.3608G>T; NP_000029.2:p.Gly1203Val | missense | Likely benign | VUS: BP1, PM2_supporting |
| LOVD APC_001945 | NM_000038.6:c.3930G>T; NP_000029.2:p.Lys1310Asn | missense | Likely benign | VUS: BP1, PM2_supporting |
| LOVD APC_001676 | NM_000038.6:c.4316C>G; NP_000029.2:p.Pro1439Arg | missense | Likely benign | VUS: BP1, PM2_supporting |
| ClinVar 824838 | NM_000038.6:c.4370C>T; NP_000029.2:p.Ala1457Val | missense | Likely benign | VUS: BP1, PM2_supporting |
| LOVD APC_001865 | NM_000038.6:c.5249T>C; NP_000029.2:p.Val1750Ala | missense | Likely benign | VUS: BP1, PM2_supporting |
| ClinVar 482255 | NM_000038.6:c.5651C>G; NP_000029.2:p.Ala1884Gly | missense | Likely benign | VUS: BP1 |
| LOVD APC_001947 | NM_000038.6:c.5731C>A; NP_000029.2:p.Gln1911Lys | missense | Likely benign | VUS: BP1, PM2_supporting |
| ClinVar 617981 | NM_000038.6:c.5839A>C; NP_000029.2:p.Thr1947Pro | missense | Likely benign | VUS: BP1, PM2_supporting |
| ClinVar 236654 | NM_000038.6:c.8255A>C; NP_000029.2:p.Asn2752Thr | missense | Benign | VUS: BP1 |

***Variants in the flanking intronic region with unknown consequences***

| Database ID | HGVSc; HGVSp | Predicted consequences | Prior classification | Reclassification by APC-specific criteria |
|---|---|---|---|---|
| ClinVar 482476 | NM_000038.6:c.135+1G>T | splice | **Likely pathogenic** | VUS: PP3, PM2_supporting |
| ClinVar 490194 | NM_000038.6:c.135+2T>C | splice | **Likely pathogenic** | VUS: PP3, PM2_supporting |
| ClinVar, LOVD 469955 | NM_000038.6:c.423-9A>G | splice | **Pathogenic** | VUS: PP3, PM2_supporting, PS3_moderate |
| LOVD APC_000602 | NM_000038.6:c.423-6_424delinsGAAGCAAGATCAG | splice | **pathogenic** | VUS: PM2_supporting, PS4_supporting |
| LOVD APC_000624 | NM_000038.6:c.531+1del | splice | **pathogenic** | VUS: PP3, PM2_supporting, PM6, PS4_supporting |
| ClinVar, LOVD 537529 | NM_000038.6:c.531+5_531+8del | splice | **Likely pathogenic** | VUS: PP3, PM2_supporting, PS3_moderate, PS4_supporting |
| ClinVar, LOVD 428099 | NM_000038.6:c.531+3A>C | splice | **Likely pathogenic** | VUS: PP3, PM2_supporting |
| ClinVar 127305 | NM_000038.6:c.531+5G>A | splice | **Pathogenic** | VUS: PP3, PM2_supporting, PS4_supporting, PS1_moderate |
| LOVD APC_001369 | NM_000038.6:c.532-2_532-1insAAAC | splice | **pathogenic** | VUS: BP4, PM2_supporting |
| LOVD APC_000429 | NM_000038.6:c.645+1G>C | splice | **pathogenic** | VUS: PVS1_moderate, PM2_supporting, PS1_moderate |
| LOVD APC_001803 | NM_000038.6:c.645+2T>C | splice | **pathogenic** | VUS: PVS1_moderate, PM2_supporting, PS1_moderate |
| ClinVar 185659 | NM_000038.6:c.645+2T>G | splice | **Likely pathogenic** | VUS: PVS1_moderate, PM2_supporting, PS1_moderate |
| ClinVar 822326 | NM_000038.6:c.835-17A>G | splice | **Likely pathogenic** | VUS: PM2_supporting, PS3_moderate |
| ClinVar, LOVD 433614 | NM_000038.6:c.835-7T>G | splice | **Likely pathogenic** | VUS: PP3, PM2_supporting, PS3_moderate |
| LOVD APC_000327 | NM_000038.6:c.933+1del | splice | **pathogenic** | VUS: PP3, PM2_supporting, PS4_supporting |
| ClinVar 181775 | NM_000038.6:c.1312+3_1312+4del | splice | **Pathogenic** | VUS: PP3, PM2_supporting |
| ClinVar 486792 | NM_000038.6:c.1312+3A>C | splice | **Likely pathogenic** | VUS: PP3, PM2_supporting, PS1_moderate |
| LOVD APC_001939 | NM_000038.6:c.1312+4_1312+19del | splice | **pathogenic** | VUS: BP4, PM2_supporting |
| ClinVar, LOVD 265372 | NM_000038.6:c.1312+5G>C | splice | **Likely pathogenic** | VUS: PM2_supporting, PS4_supporting, PS1_moderate |
| LOVD APC_001275 | NM_000038.6:c.1312+8C>T | splice | **pathogenic** | VUS: BP4, PM2_supporting |
| ClinVar 1066995 | NM_000038.6:c.1313-2A>C | splice | **Likely pathogenic** | VUS: PP3, PM2_supporting |
| ClinVar 578480 | NM_000038.6:c.1313-2A>G | splice | **Likely pathogenic** | VUS: PP3, PM2_supporting, PS3_moderate |
| ClinVar 1339656 | NM_000038.6:c.1313-1G>T | splice | **Likely pathogenic** | VUS: PP3, PM2_supporting |

| | | | | |
|---|---|---|---|---|
| ClinVar 1323307 | NM_000038.6:c.1313-1G>C | splice | **Pathogenic** | VUS: PP3, PM2_supporting |
| LOVD APC_000792 | NM_000038.6:c.1408+7C>G | splice | **pathogenic** | VUS: BP4, PM2_supporting, PS4_supporting |
| ClinVar, LOVD 411406 | NM_000038.6:c.1409-5A>G | splice | **Pathogenic** | VUS: PP3, PM2_supporting, PS3_moderate, PS4_supporting |
| ClinVar, LOVD 485146 | NM_000038.6:c.1409-3T>G | splice | **Likely pathogenic** | VUS: PP3, PM2_supporting, PS3_moderate |
| LOVD APC_001291 | NM_000038.6:c.1548_1548+1delinsTT | splice | **pathogenic** | VUS: BP4, PM2_supporting |
| LOVD APC_001083 | NM_000038.6:c.1548+1del | splice | **pathogenic** | VUS: PP3, PM2_supporting |
| LOVD APC_001292 | NM_000038.6:c.1548+1_1548+9del | splice | **pathogenic** | VUS: PP3, PM2_supporting |
| ClinVar 265375 | NM_000038.6:c.1548+3_1548+4del | splice | **Likely pathogenic** | VUS: PP3, PM2_supporting |
| LOVD APC_001727 | NM_000038.6:c.1549-3C>G | splice | **Likely pathogenic** | VUS: PP3, PM2_supporting |
| LOVD APC_000214 | NM_000038.6:c.1627-8A>G | splice | **pathogenic** | VUS: PP3, PM2_supporting |
| LOVD APC_000321 | NM_000038.6:c.1742A>G; NP_000029.2:p.Lys581Arg | splice | **pathogenic** | VUS: PM2_supporting, PS3_moderate |
| ClinVar 428153 | NM_000038.6:c.1743G>C; NP_000029.2:p.Lys581Asn | splice | **Likely pathogenic** | VUS: PVS1_strong, PM2_supporting |
| LOVD APC_001411 | NM_000038.6:c.1743+1del | splice | **pathogenic** | VUS: PP3, PM2_supporting |
| LOVD APC_000296 | NM_000038.6:c.1744-17_1744-5delinsTC | splice | **pathogenic** | VUS: BP4, PM2_supporting |
| ClinVar 664704 | NM_000038.6:c.1744-6_1744-4delinsAG | splice | **Pathogenic** | VUS: PP3, PM2_supporting |
| ClinVar 433625 | NM_000038.6:c.1744-4C>G | splice | **Likely pathogenic** | VUS: PM2_supporting |
| ClinVar 819988 | NM_000038.6:c.1744-3T>G | splice | **Pathogenic** | VUS: PP3, PM2_supporting |
| LOVD APC_001467 | NM_000038.6:c.1954_1958+15del | splice | **pathogenic** | VUS: BP4, PM2_supporting |
| LOVD APC_001445 | NM_000038.6:c.1958+1del | splice | **pathogenic** | VUS: PP3, PM2_supporting |
| ClinVar 265560 | NM_000038.6:c.1958+3A>T | splice | **Likely pathogenic** | VUS: PP3, PM2_supporting, PS1_moderate |
| ClinVar 439406 | NM_000038.6:c.-18-13T>G | splice | Benign | VUS: PM2_supporting |
| ClinVar 672308 | NM_000038.6:c.135+6A>G | splice | Likely benign | VUS: BP4, PM2_supporting |
| ClinVar 1130754 | NM_000038.6:c.135+7A>G | splice | Likely benign | VUS: BP4, PM2_supporting |
| ClinVar 918312 | NM_000038.6:c.136-13T>G | splice | Likely benign | VUS: BP4, PM2_supporting |
| ClinVar 490196 | NM_000038.6:c.136-12T>C | splice | Likely benign | VUS: BP4, PM2_supporting |
| ClinVar 490239 | NM_000038.6:c.221-11A>T | splice | Likely benign | VUS: BP4, PM2_supporting |
| LOVD APC_001759 | NM_000038.6:c.423-18_423-17insA | splice | benign | VUS: BP4, PM2_supporting |
| ClinVar 181780 | NM_000038.6:c.423-17_423-16insT | splice | Benign | VUS: BP4, PM2_supporting |
| ClinVar 1143398 | NM_000038.6:c.423-9A>T | splice | Likely benign | VUS: BP4, PM2_supporting |
| ClinVar 1116379 | NM_000038.6:c.423-8A>T | splice | Likely benign | VUS: BP4, PM2_supporting |
| ClinVar 1149314 | NM_000038.6:c.423-4A>T | splice | Likely benign | VUS: BP4, PM2_supporting |
| ClinVar 537617 | NM_000038.6:c.532-7G>T | splice | Likely benign | VUS: BP4 |
| ClinVar 380375 | NM_000038.6:c.532-7G>C | splice | Likely benign | VUS: BP4 |
| ClinVar 1110067 | NM_000038.6:c.645+7T>C | splice | Likely benign | VUS: BP4, PM2_supporting |
| ClinVar 1137468 | NM_000038.6:c.645+8A>C | splice | Likely benign | VUS: BP4, PM2_supporting |
| ClinVar 926761 | NM_000038.6:c.645+8A>T | splice | Likely benign | VUS: BP4, PM2_supporting |
| ClinVar 793315 | NM_000038.6:c.646-8T>C | splice | Likely benign | VUS: BP4, PM2_supporting |
| ClinVar 490358 | NM_000038.6:c.729+3T>A | splice | Likely benign | VUS: BP4, PM2_supporting |
| ClinVar 1103461 | NM_000038.6:c.729+8A>G | splice | Likely benign | VUS: BP4, PM2_supporting |

***Deep intronic, synonymous, in-frame variants and variants in the UTR that are unable to be classified due to missing additional information***

| **Database_ID** | **HGVSc; HGVSp** | **Predicted consequences** | **Prior classification** | **Reclassification by APC-specific criteria** |
|---|---|---|---|---|
| LOVD APC_001244 | NM_000038.6:c.1408+735A>T | intron | **pathogenic** | VUS: PM2_supporting, PS3_moderate, PS4_supporting |
| ClinVar 823173 | NM_000038.6:c.933+829A>G | intron | **Likely pathogenic** | VUS: PM2_supporting |
| ClinVar 1111220 | NM_001127511.3:c.-199C>T | UTR | Likely benign | VUS: BP4, PM2_supporting |
| ClinVar 1169564 | NM_001127511.3:c.-167_-166insG | UTR | Benign | VUS: BP4, PM2_supporting |
| ClinVar 641353 | NM_001127511.3:c.-134_-133insGGG | UTR | Likely benign | VUS: BP4, PM2_supporting |
| ClinVar 1164593 | NM_001127511.3:c.-133_-132delinsGT | UTR | Benign | VUS: PM2_supporting |
| LOVD APC_001812 | NM_001127511.3:c.-126dup | UTR | Likely benign | VUS: PM2_supporting |
| ClinVar 1316326 | NM_001127511.3:c.-126_-125insGA | UTR | Likely benign | VUS: BP4, PM2_supporting |
| ClinVar 469825 | NM_001127511.3:c.-124C>G | UTR | Benign | VUS: BP4, PM2_supporting |
| LOVD APC_001815 | NM_001127511.3:c.15G>C; NP_001120983.2:p.Gly6= | UTR | Likely benign | VUS: PM2_supporting |
| ClinVar 918662 | NM_000038.6:c.730-19G>T | intron | Likely benign | VUS: BP4, PM2_supporting |
| ClinVar 796802 | NM_000038.6:c.834+7A>G | splice | Likely benign | VUS: BP4, PM2_supporting |
| ClinVar 628015 | NM_000038.6:c.835-20A>C | intron | Likely benign | VUS: BP4, PM2_supporting |
| ClinVar 381344 | NM_000038.6:c.835-15G>A | splice | Likely benign | VUS: BP4, PM2_supporting |
| ClinVar 1332031 | NM_000038.6:c.835-13G>A | splice | Likely benign | VUS: BP4, PM2_supporting |
| ClinVar 1115430 | NM_000038.6:c.835-10T>C | splice | Likely benign | VUS: PM2_supporting |
| LOVD APC_000439 | NM_000038.6:c.933G>A; NP_000029.2:p.Lys311= | splice | benign | VUS: PVS1_supporting, PM2_supporting |
| ClinVar 927827 | NM_000038.6:c.934-7C>T | splice | Likely benign | VUS: BP4, PM2_supporting |
| ClinVar 823565 | NM_000038.6:c.993G>T; NP_000029.2:p.Ser331= | synonymous | Likely benign | VUS |
| ClinVar 734263 | NM_000038.6:c.1071C>A; NP_000029.2:p.Ile357= | synonymous | Likely benign | VUS: PM2_supporting |
| ClinVar 1119186 | NM_000038.6:c.1312+8C>G | splice | Likely benign | VUS: BP4, PM2_supporting |
| ClinVar 928340 | NM_000038.6:c.1313-15G>T | splice | Likely benign | VUS: BP4, PM2_supporting |
| ClinVar 921987 | NM_000038.6:c.1313-14del | splice | Likely benign | VUS: BP4, PM2_supporting |

| | | | | |
|---|---|---|---|---|
| ClinVar 918459 | NM_000038.6:c.1313-13T>C | splice | Likely benign | VUS: BP4 |
| ClinVar 416761 | NM_000038.6:c.1313-8T>A | splice | Likely benign | VUS: BP4, PM2_supporting |
| ClinVar 819130 | NM_000038.6:c.1407A>G; NP_000029.2:p.Leu469= | splice | Likely benign | VUS: BP4 |
| ClinVar 919829 | NM_000038.6:c.1408+7C>T | splice | Likely benign | VUS: BP4, PM2_supporting |
| ClinVar 1128391 | NM_000038.6:c.1408+8A>T | splice | Likely benign | VUS: BP4, PM2_supporting |
| ClinVar 388511 | NM_000038.6:c.1408+8A>G | splice | Likely benign | VUS: BP4, PM2_supporting |
| ClinVar 371848 | NM_000038.6:c.1409-17T>G | splice | Likely benign | VUS: BP4 |
| ClinVar 923782 | NM_000038.6:c.1409-16G>A | splice | Likely benign | VUS: BP4 |
| ClinVar 922204 | NM_000038.6:c.1409-16G>C | splice | Likely benign | VUS: BP4, PM2_supporting |
| ClinVar 508018 | NM_000038.6:c.1409-13C>G | splice | Likely benign | VUS: BP4, PM2_supporting |
| ClinVar 922965 | NM_000038.6:c.1410G>T; NP_000029.2:p.Gly470= | splice | Likely benign | VUS: BP4, PM2_supporting |
| ClinVar 630114 | NM_000038.6:c.1626+8T>G | splice | Likely benign | VUS: BP4, PM2_supporting |
| ClinVar 490219 | NM_000038.6:c.1626+8T>C | splice | Likely benign | VUS: BP4, PM2_supporting |
| ClinVar 490220 | NM_000038.6:c.1627-17A>G | splice | Likely benign | VUS: PM2_supporting |
| ClinVar 627826 | NM_000038.6:c.1627-16A>C | splice | Likely benign | VUS: BP4, PM2_supporting |
| ClinVar 490223 | NM_000038.6:c.1744-20C>T | intron | Likely benign | VUS: BP4, PM2_supporting |
| ClinVar 548881 | NM_000038.6:c.1744-14_1744-13del | splice | Likely benign | VUS: BP4, PM2_supporting |
| LOVD APC_001729 | NM_000038.6:c.1744-11T>G | splice | Likely benign | VUS: PM2_supporting |
| ClinVar 516255 | NM_000038.6:c.1744-10T>C | splice | Likely benign | VUS: BP4, PM2_supporting |
| LOVD APC_000607 | NM_000038.6:c.1869G>T; NP_000029.2:p.Arg623= | synonymous | benign | VUS: BP4, BP7, PM2_supporting, PS3_moderate, PS4 |
| ClinVar 627857 | NM_000038.6:c.1959-18C>G | intron | Likely benign | VUS: BP4, PM2_supporting |
| ClinVar 630908 | NM_000038.6:c.1959-17T>C | splice | Likely benign | VUS: BP4, PM2_supporting |
| LOVD APC_000458 | NM_000038.6:c.1959G>C; NP_000029.2:p.Arg653Ser | splice | benign | VUS: PM2_supporting |
| ClinVar 389718 | NM_000038.6:c.1962A>G; NP_000029.2:p.Gln654= | synonymous | Likely benign | VUS: PM2_supporting |
| ClinVar 733258 | NM_000038.6:c.2031C>G; NP_000029.2:p.Val677= | synonymous | Likely benign | VUS |
| ClinVar 918747 | NM_000038.6:c.4966_4967delinsAG; NP_000029.2:p.Ser1656= | synonymous | Likely benign | VUS: PM2_supporting |
| ClinVar 439411 | NM_000038.6:c.5265_5268delinsATCG; NP_000029.2:p.AlaSer1755= | synonymous | Benign | VUS: PM2_supporting |
| ClinVar 757607 | NM_000038.6:c.7404_7406del; NP_000029.2:p.Ser2469del | inframe | Likely benign | VUS: BP4, PM2_supporting |
| ClinVar 802150 | NM_000038.6:c.*415_*414insAAAAAA | UTR | Likely benign | VUS: PM2_supporting |
| ClinVar 217930 | NM_000038.6:c.1525_1527del; NP_000029.2:p.Thr509del | inframe | **Likely pathogenic** | VUS: BP4, PM2_supporting |
| LOVD APC_000075 | NM_000038.6:c.2546_2551del; NP_000029.2:p.Asp849_Ser851delinsGly | inframe | **pathogenic** | VUS: BP4, PM2_supporting, PS4_moderate |
| LOVD APC_000883 | NM_000038.6:c.3542_3568del; NP_000029.2:p.Leu1181_Ser1189del | inframe | **pathogenic** | VUS: BP4, PM2_supporting |
| ***Truncating variants at the 3' end of the gene and therefore excluded from the application of PVS1*** | | | | |
| **Database_ID** | **HGVSc; HGVSp** | **Predicted** | **Prior_classification** | **Reclassification by APC-specific criteria** |
| ClinVar 545875 | NM_000038.6:c.7946_7955del; NP_000029.2:p.Pro2649LeufsTer8 | frameshift | **Pathogenic** | VUS: PM2_supporting |
| ClinVar 545737 | NM_000038.6:c.7959_7962del; NP_000029.2:p.Thr2654ArgfsTer5 | frameshift | **Pathogenic** | VUS: PM2_supporting |
| ClinVar 428168 | NM_000038.6:c.8047del; NP_000029.2:p.Ile2683LeufsTer40 | frameshift | **Pathogenic** | VUS: PM2_supporting |
| ClinVar 827446 | NM_000038.6:c.8099_8102del; NP_000029.2:p.Asn2700ArgfsTer22 | frameshift | **Likely pathogenic** | VUS: PM2_supporting |
| LOVD APC_001492 | NM_000038.6:c.8344del; NP_000029.2:p.Thr2782LeufsTer28 | frameshift | **pathogenic** | VUS: PM2_supporting |
| ClinVar, LOVD 486740 | NM_000038.6:c.8514C>A; NP_000029.2:p.Tyr2838Ter | nonsense | **Likely pathogenic** | VUS: PM2_supporting |
| ClinVar 233392 | NM_000038.6:c.8514C>G; NP_000029.2:p.Tyr2838Ter | nonsense | **Likely pathogenic** | VUS: PM2_supporting |

*Note: Classification based on the ClinGen InSiGHT Hereditary Colorectal Cancer/Polyposis Expert Panel Specifications to the ACMG/AMP Variant Interpretation Guidelines for APC Version 1.0.0. Criteria applied in a stepwise fashion using a classification algorithm, not all available evidence may have been considered to arrive at current classification. All classifications are preliminary.*

**Table S4 (A)** Frequency of application of the APC-specific variant classification criteria

| Criteria | Variant count for each criteria applied | | | | | | |
|---|---|---|---|---|---|---|---|
| Code_weight | supporting | moderate | strong | very strong | stand alone | Total count | Total % |
| PM2_supporting | 7083 | | | | | 7083 | 69.3% |
| BP1 | 4220 | | | | | 4220 | 41.3% |
| BP4 | 2789 | | | | | 2789 | 27.3% |
| BP7 | 2236 | | | | | 2236 | 21.9% |
| PVS1_variable | 1 | 5 | 22 | 2164 | | 2192 | 21.4% |
| BS1 | | | 2089 | | | 2089 | 20.4% |
| PS4_variable | 281 | 93 | 68 | 12 | | 454 | 4.4% |
| BA1 | | | | | 427 | 427 | 4.2% |
| BS2_variable | 109 | | 46 | | | 155 | 1.5% |
| PP3 | 60 | | | | | 60 | 0.6% |
| PS3_variable | 7 | 32 | 5 | 4 | | 48 | 0.5% |
| PS1_variable | | 38 | 3 | | | 41 | 0.4% |
| PS2_variable | | 15 | 1 | | | 16 | 0.2% |
| PM6_variable | 5 | 9 | 0 | 1 | | 15 | 0.1% |
| No code applied | | | | | | 11 | 0.1% |
| BS3_variable | 10 | | 0 | | | 10 | 0.1% |
| PP1_variable | 5 | 1 | 2 | | | 8 | 0.1% |
| BP5 | 8 | | | | | 8 | 0.1% |
| PM5_variable | 5 | 0 | | | | 5 | 0.0% |
| BS4_variable | 2 | | 0 | | | 2 | 0.0% |
| BP2 | 1 | | | | | 1 | 0.0% |

**Table S4(B)** Most frequent APC-specific variant classification codes and code combinations resulting in non-VUS classifications

| Codes | Classification outcome | Number of variants | % of all variants (n=10,228) | Number of previous VUS | % of all previous VUS (n=6,142) [d] | McNemar's χ2 (p value) |
|---|---|---|---|---|---|---|
| **PVS1, PM2_supporting** [a] | Likely pathogenic | 2138 | 20.9% | 46 | 0.7% | 40.02 ($p < 0.05$) |
| **BS1** [b] | Likely benign | 2071 | 20.2% | 1542 | 25.1% | 1540 ($p < 0.05$) |
| **BP4, BP7** [c] | Likely benign | 1669 | 16.3% | 687 | 11.2% | 685 ($p < 0.05$) |
| **BA1** | Benign | 427 | 4.2% | 208 | 3.4% | 206 ($p < 0.05$) |

[a] for 431 of these variants one additional code with at least supporting evidence could be applied (e. g. PS4_supporting), resulting in a final classification as pathogenic

[b] for 1881 of these variants additional codes could be applied (BP1, BP4, BP7 and/or BS2_supporting) without influence on the classification outcome; for 53 variants at least on additional strong code (BS2) could be applied, resulting in a classification as benign

[c] for 1505 of these variants, PM2_supporting was applicable, which was not considered as a conflict based on the APC-specific criteria

[d] % of all VUS that could be reclassified into the respective meaningful class based on this code (combination)
