# Supplementary material for: Large-scale application of ClinGen-InSiGHT APC-specific ACMG/AMP variant classification criteria leads to substantial reduction in VUS
Source: Am J Hum Genet. 2024 Oct 1;111(11):2427–43. doi: 10.1016/j.ajhg.2024.09.002 (PMC11568752; doi:10.1016/j.ajhg.2024.09.002)
Supplement: Document S2. Article plus supplemental information [file mmc3.pdf]

Large-scale application of ClinGen-InSiGHT *APC*-specific ACMG/AMP variant classification criteria leads to substantial reduction in VUS

Graphical abstract

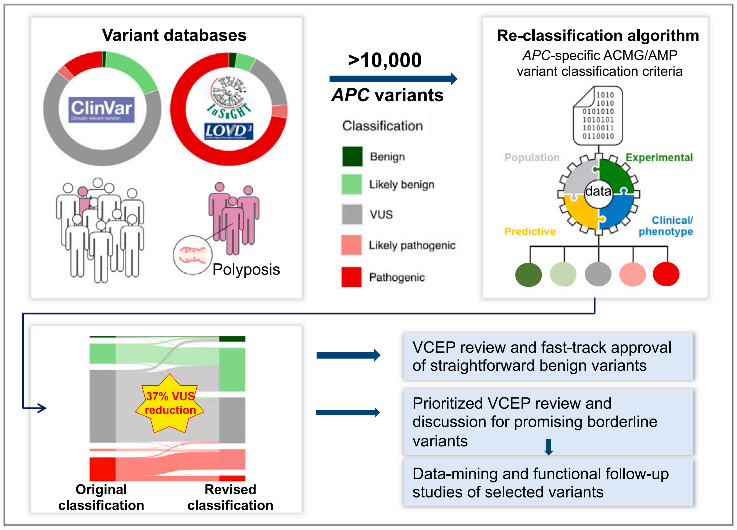

Authors

Xiaoyu Yin, Marcy Richardson, Andreas Laner, ..., Finlay A. Macrae, Isabel Spier, Stefan Aretz

Correspondence

stefan.aretz@uni-bonn.de

Application of the gene-specific ACMG/AMP variant classification criteria to >10,000 publicly available germline *APC* variants through a streamlined algorithm reduced the fraction of VUSs by 37%, demonstrating the feasibility of a systematic classification approach in large datasets. This study might serve as a model for other variant interpretation initiatives.

Yin et al., 2024, The American Journal of Human Genetics *111*, 2427–2443
November 7, 2024 © 2024 The Author(s). Published by Elsevier Inc. on behalf of American Society of Human Genetics.
https://doi.org/10.1016/j.ajhg.2024.09.002

Large-scale application of ClinGen-InSiGHT *APC*-specific ACMG/AMP variant classification criteria leads to substantial reduction in VUS

Xiaoyu Yin,[1,2,3] Marcy Richardson,[4] Andreas Laner,[5] Xuemei Shi,[6] Elisabet Ognedal,[7] Valeria Vasta,[8] Thomas v.O. Hansen,[9,10] Marta Pineda,[11,12,13] Deborah Ritter,[14,15] Johan de Dunnen,[16] Emadeldin Hassanin,[17,18] Wencong Lyman Lin,[19] Ester Borras,[20] Karl Krahn,[21] Margareta Nordling,[22,23] Alexandra Martins,[24] Khalid Mahmood,[25] Emily Nadeau,[26] Victoria Beshay,[27] Carli Tops,[16] Maurizio Genuardi,[28] Tina Pesaran,[4] Ian M. Frayling,[29,30,31] Gabriel Capellá,[11,12,13] Andrew Latchford,[29,32] Sean V. Tavtigian,[33,34] Carlo Maj,[17,35] Sharon E. Plon,[14,15] Marc S. Greenblatt,[26] Finlay A. Macrae,[1,2] Isabel Spier,[3,11,36,37] and Stefan Aretz[3,11,36,37,*]

Summary

Pathogenic constitutional *APC* variants underlie familial adenomatous polyposis, the most common hereditary gastrointestinal polyposis syndrome. To improve variant classification and resolve the interpretative challenges of variants of uncertain significance (VUSs), APC-specific variant classification criteria were developed by the ClinGen-InSiGHT Hereditary Colorectal Cancer/Polyposis Variant Curation Expert Panel (VCEP) based on the criteria of the American College of Medical Genetics and Genomics and the Association for Molecular Pathology (ACMG/AMP). A streamlined algorithm using the *APC*-specific criteria was developed and applied to assess all *APC* variants in ClinVar and the International Society for Gastrointestinal Hereditary Tumours (InSiGHT) international reference *APC* Leiden Open Variation Database (LOVD) variant database, which included a total of 10,228 unique *APC* variants. Among the ClinVar and LOVD variants with an initial classification of (likely) benign or (likely) pathogenic, 94% and 96% remained in their original categories, respectively. In contrast, 41% ClinVar and 61% LOVD VUSs were reclassified into clinically meaningful classes, the vast majority as (likely) benign. The total number of VUSs was reduced by 37%. In 24 out of 37 (65%) promising *APC* variants that remained VUS despite evidence for pathogenicity, a data-mining-driven work-up allowed their reclassification as (likely) pathogenic. These results demonstrated that the application of *APC*-specific criteria substantially reduced the number of VUSs in ClinVar and LOVD. The study also demonstrated the feasibility of a systematic approach to variant classification in large datasets, which might serve as a generalizable model for other gene- or disease-specific variant interpretation initiatives. It also allowed for the prioritization of VUSs that will benefit from in-depth evidence collection. This subset of *APC* variants was approved by the VCEP and made publicly available through ClinVar and LOVD for widespread clinical use.

Introduction

Familial adenomatous polyposis (FAP; MIM: 175100) is an autosomal-dominant precancerous condition and the most common monogenic gastrointestinal polyposis syndrome caused by constitutional (germline) pathogenic variants (PVs) in the tumor suppressor gene *APC* (MIM: 611731).[1–3] The colorectal phenotype exhibits high

[1]Department of Colorectal Medicine and Genetics, Royal Melbourne Hospital, Parkville, VIC, Australia; [2]Department of Medicine, University of Melbourne, Parkville, VIC, Australia; [3]Institute of Human Genetics, Medical Faculty, University of Bonn, Bonn, Germany; [4]Ambry Genetics, Aliso Viejo, CA, USA; [5]Medical Genetics Center Munich, MGZ Munich, Germany; [6]Greenwood Genetic Center, Greenwood, SC, USA; [7]Western Norway Familial Cancer Center, Haukeland University Hospital, Bergen, Norway; [8]Northwest Genomics Center, Department of Genome Sciences, University of Washington, Seattle, WA, USA; [9]Department of Clinical Genetics, Rigshospitalet, Copenhagen University Hospital, Copenhagen, Denmark; [10]Department of Clinical Medicine, Faculty of Health and Medical Sciences, University of Copenhagen, Copenhagen, Denmark; [11]European Reference Network on Genetic Tumour Risk Syndromes (ERN GENTURIS), Nijmegen, the Netherlands; [12]Hereditary Cancer Program, Catalan Institute of Oncology – ONCOBELL, IDIBELL, Barcelona, Spain; [13]Centro de Investigación Biomédica en Red de Cáncer (CIBERONC), Instituto Salud Carlos III, Madrid, Spain; [14]Baylor College of Medicine, Houston, TX, USA; [15]Texas Children's Cancer Center, Texas Children's Hospital, Houston, TX, USA; [16]Departments of Human Genetics & Clinical Genetics, Leiden University Medical Center, Leiden, the Netherlands; [17]Institute for Genomic Statistics and Bioinformatics, University Hospital Bonn, Bonn, Germany; [18]Luxembourg Centre for Systems Biomedicine, University of Luxembourg, Esch-sur-Alzette, Luxembourg; [19]St Vincents Hospital Melbourne, East Melbourne, VIC, Australia; [20]Invitae Corporation, San Francisco, CA, USA; [21]GeneDx, Gaithersburg, MD, USA; [22]Department of Biomedical and Clinical Sciences, Linköping University, Linköping, Sweden; [23]Department of Clinical Genetics, Linköping University Hospital, Linköping, Sweden; [24]Université de Rouen Normandie, Inserm U1245, 76000 Rouen, France; [25]Colorectal Oncogenomics Group, Department of Clinical Pathology, University of Melbourne, Melbourne, VIC, Australia; [26]Department of Medicine, Larner College of Medicine, University of Vermont, Burlington, VT, USA; [27]Peter MacCallum Cancer Centre, Melbourne, VIC, Australia; [28]Fondazione Policlinico Universitario A. Gemelli IRCCS, and Dipartimento di Scienze della Vita e Sanità Pubblica, Università Cattolica del Sacro Cuore, Rome, Italy; [29]Polyposis Registry, St Mark's Hospital, London, UK; [30]Inherited Tumour Syndromes Research Group, Institute of Cancer & Genetics, Cardiff University, Cardiff, UK; [31]National Centre for Colorectal Disease, St Vincent's University Hospital, Dublin, Ireland; [32]Department of Surgery and Cancer, Imperial College, London, UK; [33]Huntsman Cancer Institute, University of Utah, Salt Lake City, UT, USA; [34]Department of Oncological Sciences, School of Medicine, University of Utah, Salt Lake City, UT, USA; [35]Centre for Human Genetics, University of Marburg, Marburg, Germany; [36]National Center for Hereditary Tumor Syndromes, University Hospital Bonn, Bonn, Germany
[37]These authors contributed equally
*Correspondence: stefan.aretz@uni-bonn.de
https://doi.org/10.1016/j.ajhg.2024.09.002.
© 2024 The Author(s). Published by Elsevier Inc. on behalf of American Society of Human Genetics.
This is an open access article under the CC BY license (http://creativecommons.org/licenses/by/4.0/).

inter- and intra-familial variability from the growth of less than 100 up to thousands of adenomatous polyps.[4] Surveillance colonoscopy and/or prophylactic (procto)colectomy are warranted to prevent colorectal cancer or delay disease progression.[5–7] The identification of an *APC* PV therefore has direct relevance for individuals and their relatives, defining *APC* as a highly clinically actionable gene.[8] Depending on the colorectal phenotype and family history, causative *APC* variants can be identified in up to 85% of individuals with adenomatous polyposis,[4,9–13] the vast majority of which are nonsense and frameshift variants leading to a truncated protein with abrogated function.[5–7,14] During the last three decades, thousands of rare *APC* PVs have been identified in patients with FAP. Variants are distributed across the gene, the majority of which are private, observed in only one or very few families. Concurrently, the widespread use of large multi-gene panel testing and exome or genome sequencing have generated an additional plethora of *APC* variants in (healthy) individuals without a polyposis phenotype, many of which are missense alterations. In the absence of comprehensive data and consensus for the level of evidence required to corroborate variant interpretation, most of these variants remain variants of uncertain significance (VUSs) or variants with conflicting assertions, accounting for around 67% of *APC* variants in ClinVar. These VUSs confer diagnostic uncertainty and pose challenges in clinical practice.

Since its inception, the American College of Medical Genetics and Genomics and the Association for Molecular Pathology (ACMG/AMP) guidelines have evolved through further refinement to the various variant assessment methods and evidence codes[15–21] and the development of gene- or disease-specific ACMG/AMP classification criteria by variant curation expert panels (VCEPs) under the governance of ClinGen (Clinical Genome Resource).[22] The International Society for Gastrointestinal Hereditary Tumours (InSiGHT) houses and curates the world's largest databases for variants of gastrointestinal-cancer-predisposing genes in the Leiden Open Variation Database (LOVD).[23] Recently, a ClinGen-InSiGHT Hereditary Colorectal Cancer/Polyposis VCEP (HCCP VCEP) was established. The *APC* subcommittee (*APC* VCEP) developed and validated *APC*-specific ACMG/AMP classification criteria,[24] readying the VCEP for variant submissions to ClinVar as an FDA-recognized expert panel. The most updated version of the VCEP specifications can be found in the online criteria specification registry.

In this study, we used the *APC*-specific criteria to perform a large-scale reclassification exercise of all *APC* variants listed in ClinVar and the InSiGHT *APC* reference database LOVD. The criteria were embedded and applied in a streamlined algorithm, which was supplemented by further data mining and curation to achieve the most accurate classification. The results were compared to their original assertions in respective databases, and any discrepancies were addressed.

Methods

APC variant database merging and centralization

Prior to the extraction of variants, the landscape of all publicly available databases containing *APC* variants was identified and examined for activity and curation status. Of at least 19 *APC* databases, nine are inactive and another three are not curated (Table S1). All listed curators, in particular those of inactive, outdated, or orphaned databases, were contacted to request sharing and merging of data with the reference LOVD (v.3.0) installation. To establish a centralized, curated data source of *APC* variants with consistent reporting format and phenotypic description, the InSiGHT *APC* LOVD and the Global Variome shared LOVD were subsequently merged to generate one international reference *APC* variant database in LOVD, abbreviated in the following as LOVD and accessible via all three URLs.

ClinVar and LOVD variant extraction and annotation

ClinVar variants with summary evaluation and individual submitter annotations were retrieved from ClinVar in March 2022 (https://ftp.ncbi.nlm.nih.gov/pub/clinvar/xml/). The complete public dataset was downloaded, and all alleles associated with *APC* were extracted. The merged *APC* LOVD database was downloaded on May 12, 2022. The legacy description of published variants was recorded alongside their standardized nomenclature as per the Human Genome Variation Society (HGVS) guidelines on the preferred reference transcript GenBank: NM_000038.6,[25] correcting for any errors where possible. All non-structural variants were annotated using the Ensembl Variant Effect Predictor (VEP).[26] Structural variants defined by genomic alterations greater than 50 bp in size (gross deletions, duplications, inversions, in-frame, Alu and SVA retrotransposon insertions, inversions, and complex variants) were annotated manually using Mutalyzer.[27]

Reclassification algorithm

Details of the *APC*-specific criteria were as published previously and summarized in Figure 1.[24] A stepwise algorithm encompassing all evidence codes was designed to systematically evaluate all *APC* variants in ClinVar and LOVD (Figure 2).

Minor allele frequency data (BA1, BS1, PM2_supporting)

The frequency of all *APC* variants in reference populations were compared against the minor allele frequency (MAF) criteria BA1 ($\geq$0.001), BS1 ($\geq$0.00001), and PM2_supporting ($\leq$0.000003 or absent; first version of criteria). The non-cancer datasets from gnomAD (the Genome Aggregation Database) v2.1.1 and v3.1.2 were used as the reference population frequency data for non-structural variants.[29] Exome sequencing data from 323,228 healthy individuals without a diagnosis of colorectal cancer (CRC) in the UK Biobank were also used to further enhance the detection of rare *APC* variants.[30] If a variant was present in multiple reference population datasets, the highest MAF was calculated from any subpopulation with more than 2,000 alleles, with the exclusion of founder populations. The frequency of structural variants was examined in gnomAD structural variants (SVs) v2.1 and the Database of Genomic Variants (DGV) Gold Standard release from May 15, 2016.[31]

Predictive data (PVS1, PP3, BP1, BP4, and BP7)

Truncating variants, canonical $\pm$1/2 splice site variants, and exonic last nucleotide guanine to non-guanine variants were assigned the loss-of-function (LoF) criterion PVS1 if they are located between

Criteria		Original ACMG/AMP criteria	APC-specific modifications in brief			
			Supporting	Moderate	Strong	Very Strong
Pathogenic criteria	PVS1	Null variant	Per modified PVS1 decision tree*			
	PS1	Same effect as a previous variant		As a Likely Pathogenic variant	As a Pathogenic variant	
	PS2	Confirmed *De novo*		1-1.5 *de novo* points*	2-3.5	≥ 4
	PS3	Functional study	RNA/protein assays as specified*			
	PS4	Phenotype in affected individual	1-1.5 phenotype points*	2-3.5	4-15.5	≥ 16
	PM2	Rare/absent in control	≤ 0.0003% if allele count > 1; < 0.001% if allele count ≤ 1			
	PM5	At the same residue as previous variant	As a Likely Pathogenic variant	As a Pathogenic variant		
	PM6	Presumed *De novo*	0.5 *de novo* points*	1-1.5 *de novo* point	2-3.5 *de novo* points	≥ 4 *de novo* points
	PP1	Co-segregation with disease	3-4 meiosis in ≥ 1 family	5-6 meiosis in ≥ 1 family	≥ 7 meiosis in ≥ 2 families	
	PP3	*In silico* prediction pathogenic	Splicing only			
Benign criteria	BA1	Allele frequency	≥ 0.1% (Stand-alone)			
	BS1	Allele frequency			≥ 0.001%	
	BS2	Presence in healthy unaffected control	≥ 3 points for healthy individuals*		≥ 10 points for healthy individuals*	
	BS3	Functional study	RNA/protein assays as specified*			
	BS4	Lack of segregation	≥ 1 affected individual with 0.5 phenotype points*		≥ 1 affected individual with 1 phenotype point*	
	BP1	Missense variant in a gene where only LOF cause disease	Exclude 1st 15-amino acid repeat			
	BP2	Co-occurrence with pathogenic variant	≥ 1 *in trans* or ≥ 3 in an unknown phase			
	BP4	*In silico* prediction benign	Splicing only			
	BP5	Alternative cause of colorectal polyposis	Unchanged			
	BP7	Silent/intronic variant without splice effect	Silent &+7/-21 intronic variant			

Codes deemed not applicable: PM1, PM3, PM4, PP2, PP4, PP5, BP3, BP6

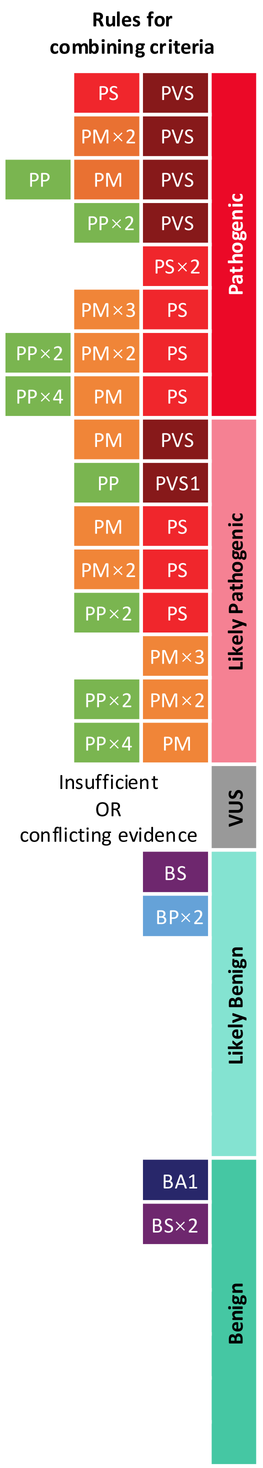

Figure 1. ***APC*-specific criteria in brief**
The ACMG/AMP guidelines defined pathogenic (P) and benign (B) criteria encompassing evidence in population, experimental, computational, and clinical domains.[28] The criteria are weighed and coded as benign stand-alone (BA), pathogenic very strong (PVS), strong (BS/PS), moderate (PM/BM), and supporting (PP/BP), the combination of which leads to a final classification of pathogenic (P), likely pathogenic (LP), variant of uncertain significance (VUS), likely benign (LB), or benign (B). This figure is only intended as a quick reference guide to the *APC*-specific criteria[24]; the most updated version can be found at https://cspec.genome.network/cspec/ui/svi/doc/GN089. *Details not shown here.

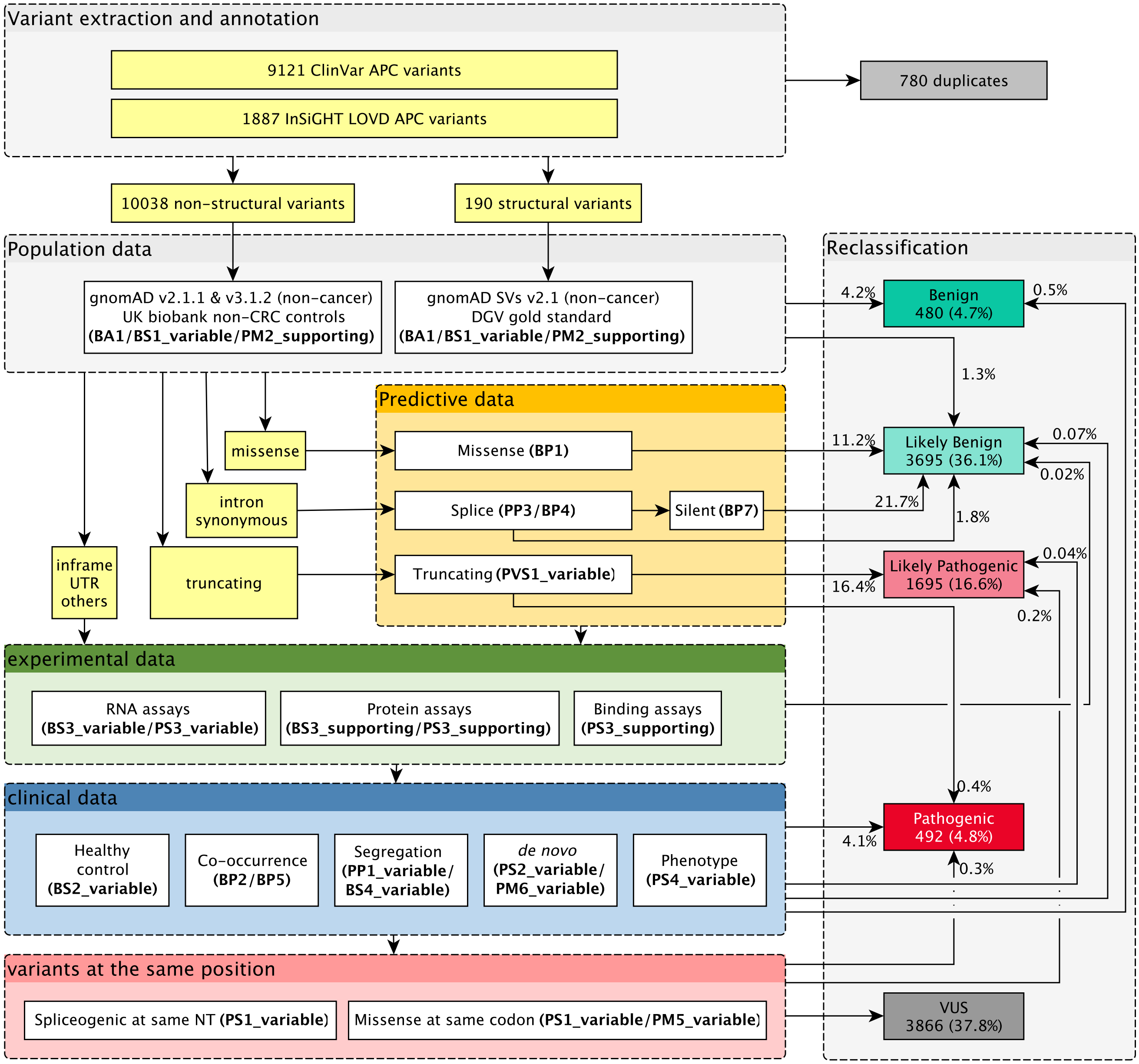

Figure 2. ***APC*-specific criteria embedded in a reclassification algorithm**
An algorithm demonstrating the application of all eligible *APC*-specific codes to *APC* variants in ClinVar and the InSiGHT LOVD in a stepwise approach, and the percentage of variants that reached a B/LB or P/LP classification at each step. Firstly, the highest MAF of non-structural and structural variants was calculated from gnomAD non-cancer datasets or UK Biobank non-colorectal cancer control data, and gnomAD structural variants (SVs) or database of genomic variants (DGV) gold standard, respectively. Predictive criteria were then applied based on the most severe variant consequence as predicted by Ensemble variant effect predictor (VEP).[26] Variants with any experimental and/or clinical evidence were identified and assigned corresponding code. Finally, splice variants at the same nucleotide and missense variants at the same codon were identified, and the variants at the same position criteria were applied.

codons 49 and 2645 inclusive.[24] Splice prediction was performed using SpliceAI and MaxEntScan via VEP, which determined PP3 and BP4 eligibility for synonymous and intronic variants and PP3 eligibility for presumed missense variants to reveal possible splicing effects.[32,33] Variants exceeding a score of 0.6 in SpliceAI and a 15% reduction from the native site prediction in MaxEntScan were considered spliceogenic, and variants with a SpliceAI score of less than 0.2 and a MaxEntScan score of less than 3 were considered to have no impact on splicing. The criteria were only applied when both prediction tools showed concordant results. BP7 was subsequently applied to synonymous and deep intronic variants at or beyond +7/−21, which satisfied BP4. The missense code BP1 was applied to missense variants located outside of the first 15-amino-acid repeat of the β-catenin binding domain (codons 1021–1035) if they were consistently deemed non-spliceogenic by SpliceAI and MaxEntScan. Computational prediction models for conservation, evolution, etc. are not applicable for the evaluation of *APC* missense variants as described previously.[24,34]

Experimental data (BS3, PS3)

Published mRNA splicing assays and protein function assays of *APC* variants were collated in a systematic review by the *APC*

VCEP to derive gene-specific recommendations for the application of the experimental criteria BS3 and PS3.[24] VCEP-approved experimental evidence for *APC* variant classifications included RNA assays for PS3 and BS3, β-catenin regulated transcriptional assays for PS3_supporting and BS3_supporting, and surface plasmon resonance assays for PS3_supporting. The proportion of aberrant transcripts, evidence of bi-allelic expression, and use of nonsense-mediated decay inhibition were also noted from the original publications when available, which determined the quality of the data and therefore the weight assigned for PS3 and BS3.

Clinical data (PS4, PS2, PM6, PP1, BS4, BP2, BP5, BS2)

The phenotype details of individuals with *APC* variants from the InSiGHT LOVD download were retrieved. In individuals where phenotype details were recorded as unstructured text, text mining was employed to extract and stratify useful information. Affected individuals were scored for PS4 as described previously.[24] Further data mining was undertaken focusing on the identification of confirmed *de novo APC* variants (PS2/PM6), segregation and non-segregation analysis (PP1/BS4), co-occurrence of the variant under assessment with other established pathogenic *APC* variant (BP2), or with an alternative molecular basis of disease (BP5), including heterozygous PVs in *POLD1* (MIM: 174761) or *POLE* (MIM: 174762); bi-allelic PVs in *MUTYH* (MIM: 604933), *NTHL1* (MIM: 602656), *MSH3* (MIM: 600887), or the MMR genes (*MLH1* [MIM: 120436], *MSH2* [MIM: 609309], *MSH6* [MIM: 600678], or *PMS2* MIM: 600259]). The HGMD and Universal Mutation Database were also examined for additional data.[35,36] For variants that were already listed in gnomAD, their presence in healthy unaffected individuals in the UK Biobank non-CRC population was regarded as additional evidence in support of a benign classification (BS2). The absolute number of heterozygous individuals in the UK Biobank non-CRC dataset was counted, scored according to the *APC* VCEP's definition for a healthy individual, and given the healthy control code BS2 with appropriate weight. As homozygous LoF *APC* variants were shown to be lethal at embryonic developmental stages,[37] the observation of variant homozygosity ≥2 times in any reference population database was also considered strong evidence for a benign classification as defined in the *APC*-specific criteria. Clinical data were obtained in accordance with the guidelines of the Ethics Committee of the Medical Faculty of the University of Bonn and the 1975 Declaration of Helsinki. Participants of clinical genetic testing gave written informed consent for their data to be used for clinical research and genetic investigations according to local regulations.

Statistical analysis

McNemar's test is used to assess dichotomous changes between VUS vs. non-VUS classifications before and after reclassification to assess the impact of the *APC*-specific criteria. The Mann-Whitney-Wilcoxon test with continuity correction was used to compare differences in distribution of classification for selected variant types before and after reclassification. Statistical analyses were performed using R software version 4.3.2 (R Project for Statistical Computing). All significance tests were 2-tailed, and $p < 0.05$ was considered statistically significant.

Review and synthesis of primary criteria combination

The *APC*-specific criteria applied for each variant were pulled together to generate a preliminary criteria combination according to the *APC* VCEP's rules for combining criteria.[24] In addition to the phenotype description in LOVD, for variants that fulfilled a reasonable set of predictive and/or experimental criteria (e.g., PVS1, PS3, or BS3) but remained unclassified, the corresponding internal clinical records and/or reference publication of the variant in question was consulted to curate additional phenotype points, and further data mining was conducted using Mastermind, LitVar, and PubMed as required to reach a non-VUS classification. Additionally, the classification of the 58 published *APC* variants in the pilot study for the development of the *APC*-specific guidelines were incorporated, adding another layer of data and expert review to the result. Finally, the revised criteria combination was used to calculate a final pathogenicity class. Missense variants at the same codon and spliceogenic variants at the same nucleotide position as established pathogenic (P) or likely pathogenic (LP) variants were identified, and the criteria PS1 or PM5 were applied accordingly.

Prioritized list of variants for further review

The revised variant classification was compared with the original pathogenicity assertion in ClinVar and LOVD. Variants with clinically significant conflict (benign/likely benign [B/LB] vs. P/LP or VUS vs. P/LP) between the original assertion and reclassification were examined for causes of discrepancy. From the variants that remained VUSs after reclassification, 37 variants close to reaching a pathogenic classification were identified (Table 1), which included (1) truncating VUSs that fulfilled PVS1 but not PM2_supporting, (2) VUSs with pathogenic *in silico* predictions, (3) VUSs with experimental findings suggestive of deleterious effect, and (4) VUSs observed in individuals with FAP-associated phenotypes. A targeted literature search was conducted to acquire further information for all 37 variants. If a clinically relevant classification could be achieved, no further work up was done (group 1 in Table 1; Figure 3). Otherwise, a standardized form was used to ask VCEP members whether these variants were available in their internal laboratory databases, including clinical information (number of polyps, etc.) and RNA analysis data for variants with suspected splice effect (group 2 in Table 1; Figure 3).

Results

Variant assessment using the reclassification algorithm based on *APC*-specific criteria

A total of 10,228 *APC* variants were analyzed in this study, which included 190 (2%) structural and 10,038 (98%) non-structural variants (Table S2). A total of 9,121 and 1,887 *APC* variants were present in ClinVar and LOVD, respectively (Figure 4); 780 (41% of LOVD) variants are shared between ClinVar and LOVD. The largest group in the ClinVar dataset is the VUS class (67%) followed by the B/LB group (20%) and P/LP group (14%). Most variants in LOVD are P/LP (76%), whereas VUS only account for 16% and B/LB variants for 8%.

Comparison between the original ClinVar or LOVD assertions and the revised classification are shown in Figures 4 and S1. By applying the *APC*-specific criteria, in the ClinVar dataset, now the largest group is the B/LB group (46%) followed by VUS (41%) and P/LP (13%). In the LOVD dataset, the majority of variants remained as P/LP (75%) followed by B/LB (16%) and VUS (10%). All the other reclassification results refer to the combined

Table 1. Further curation of selected variants remaining VUSs after application of reclassification algorithm

HGVSc	Database ID[a]	Original classification	Criteria applied by algorithm	Classification by algorithm	Further curation	Final criteria applied	Final classification of VCEP
**Group 1 previously pathogenic variants—further assessment based on literature review, data mining, and reassessment of minor allele frequency (MAF) criteria resulted in a pathogenic classification**							
c.471G>A (p.Trp157Ter)	ClinVar: 411479	pathogenic	PVS1	VUS	1.5 phenotype points[38–41]; revised MAF criteria	PVS1, PS4_supp, PM2_supp[b]	pathogenic
c.1312+4_ 1312+19del (p.?)	LOVD: APC_001939	pathogenic	BP4, PM2_supp	VUS	1 phenotype point, RNA assay result, segregated in 7 meioses[42]	PS3_mod, PS4_supp, PP1_mod, PM2_supp	likely pathogenic
c.1333C>T (p.Gln445Ter)	ClinVar: 438865	pathogenic	PVS1, BS1	VUS	1 phenotype point[43,44]; revised MAF criteria	PVS1, PS4_supp, PM2_supp[b]	pathogenic
c.2546_2551del (p.Asp849_ Ser851delinsGly)	LOVD APC_000075	pathogenic	PM2_supp, PS4_mod	VUS	error in annotation: c.2546delATAGAAG (legacy name). Correct nomenclature: c.2546_ 2552del; p.(Asp849Valfs*10), 1 phenotype point, segregation in 3 meioses[11]	PVS1, PS4_supp, PM2_supp, PP1	pathogenic
c.4669_4670del (p.Ile1557Ter)	ClinVar: 183857	pathogenic	PVS1	VUS	1 phenotype point[45,46]; revised MAF criteria	PVS1, PS4_supp, PM2_supp[b]	pathogenic
**Group 2 promising potentially pathogenic VUS—further assessment based on clinical evidence from ClinVar submitters and VCEP members and reassessment of MAF criteria**							
c.70C>T (p.Arg24Ter)	ClinVar: 184702	likely pathogenic	BS1	likely benign	1.5 phenotype points; >10 healthy individual points in total[47,48]; revised MAF criteria	PS4_supp, BS2	VUS
c.136−4A>G (p.?)	ClinVar: 925741	VUS	PP3, BS1	VUS	observed in 1 individual worth 0 phenotype points; revised MAF criteria	PM2_supp	VUS
c.156del (p.Gly53GlufsTer17)	ClinVar: 654864	pathogenic	PVS1	VUS	observed in 1 individual worth 0 phenotype points; revised MAF criteria	PVS1, PM2_supp[a]	likely pathogenic
c.203del (p.Leu68TyrfsTer2)	ClinVar: 934724	pathogenic	PVS1	VUS	observed in 4 individuals worth 1 phenotype point; revised MAF criteria	PVS1, PS4_supp, PM2_supp[b]	pathogenic
c.220+2T>A (p.?)	ClinVar: 141515	likely pathogenic	PVS1, BS1	VUS	observed in 35 individuals worth 8 phenotype points; revised MAF criteria	PVS1, PS4, PM2_supp[b]	pathogenic
c.422G>C (p.Arg141Thr)	ClinVar: 1056286	VUS	PVS1_strong, PM2_supp	VUS	observed in 3 individuals worth 0.5 phenotype points	PVS1_strong, PM2_supp	VUS
c.422G>A (p.Arg141Lys)	ClinVar: 824696	VUS	PVS1_strong	VUS	observed in 0 individual worth 0 phenotype points; revised MAF criteria	PVS1_strong, PM2_supp[b]	VUS

(*Continued on next page*)

Table 1. *Continued*

HGVSc	Database ID[a]	Original classification	Criteria applied by algorithm	Classification by algorithm	Further curation	Final criteria applied	Final classification of VCEP
c.423−9A>G (p.?)	ClinVar: 469955	pathogenic	PP3, PM2_supp, PS3_mod	VUS	observed in 2 families worth 2 phenotype points	PP3, PM2_supp, PS3_mod, PS4_mod	likely pathogenic
c.531+5_531+8del (p.?)	ClinVar: 537529	likely pathogenic	PP3, PM2_supp, PS3_mod, PS4_supp	VUS	observed in 5 individuals worth 3.5 phenotype points[49,50]	PP3, PM2_supp, PS3_mod, PS4_mod	likely pathogenic
c.531+5G>A (p.?)	ClinVar: 127305	pathogenic	PP3, PM2_supp, PS4_supp, PS1_mod	VUS	observed in 6 individuals worth 2 phenotype points[51–53]	PP3, PM2_supp, PS4_mod, PS1_mod	likely pathogenic
c.531+6T>C (p.?)	ClinVar: 576816	VUS	PP3, PM2_supp, PS3_mod	VUS	observed in 2 individuals worth 2 phenotype points	PP3, PM2_supp, PS3_mod, PS4_mod	likely pathogenic
c.623A>G (p.Gln208Arg)	LOVD: APC_000758	pathogenic	BP1, PM2_supp, PP1	VUS	1 phenotype point[54]; not observed by VCEP members	BP1, PM2_supp, PS4_supp	VUS
c.645+2T>G (p.?)	ClinVar: 185659	likely pathogenic	PVS1_mod, PM2_supp	VUS	observed in 2 individuals worth 1 phenotype point	PVS1_mod, PM2_supp, PS4_supp	VUS
c.835−17A>G (p.?)	ClinVar: 822326	likely pathogenic	PM2_supp, PS3_mod	VUS	observed in 4 individuals worth 0 phenotype points	PM2_supp, PS3_supp	VUS
c.835−7T>G (p.?)	ClinVar: 433614	likely pathogenic	PP3, PM2_supp, PS3_mod	VUS	observed in 3 families worth 2.5 phenotype points	PP3, PM2_supp, PS3_mod, PS4_mod	likely pathogenic
c.933G>C (p.Lys311Asn)	ClinVar: 1025291	likely pathogenic	PVS1_supp, PM2_supp	VUS	observed in 2 individuals worth 1.5 phenotype points[55]	PVS1_strong, PM2_supp, PS4_supp	likely pathogenic
c.1042C>T (p.?)[c]	ClinVar: 955439	conflicting (pathogenic/VUS)	PVS1	VUS	1 phenotype point[56,57]; not observed by VCEP members; revised MAF criteria	PS4_supp, PM2_supp[b], BS2_supp	VUS
c.1312+3A>C (p.?)	ClinVar: 486792	likely pathogenic	PP3, PM2_supp, PS1_mod	VUS	observed in 2 individuals worth 2 phenotype points[58]	PP3, PM2_supp, PS1, PS4_mod	likely pathogenic
c.1312+5G>C (p.?)	ClinVar: 265372	likely pathogenic	PM2_supp, PS4_supp, PS1_mod	VUS	observed in 3 individuals worth 2.5 phenotype points	PM2_supp, PS4_mod, PS1_mod, PP3	likely pathogenic
c.1408+735A>T (p.?)	LOVD: APC_001244	pathogenic	PM2_supp, PS3_mod, PS4_supp	VUS	1 phenotype point[59]; not observed by VCEP members	PS4_supp, PS3_mod, PM2_supp	VUS
c.1409−5A>G (p.?)	ClinVar: 411406	pathogenic	PP3, PM2_supp, PS3_mod, PS4_supp	VUS	observed in 5 individuals worth 3 phenotype points	PS3_mod, PS4_mod, PP3, PM2_supp	likely pathogenic
c.1409−3T>G (p.?)	ClinVar: 485146	likely pathogenic	PP3, PM2_supp, PS3_mod	VUS	observed in 4 individuals worth 2 phenotype points[55]	PS3_mod, PS4_mod, PP3, PM2_supp	likely pathogenic
c.1743G>C (p.Lys581Asn)	ClinVar: 428153	likely pathogenic	PVS1_strong, PM2_supp	VUS	observed in 7 individuals worth 1 phenotype point	PVS1_strong, PS4_supp, PM2_supp	likely pathogenic
c.1902T>G (p.Ser634Arg)	ClinVar: 231954	conflicting (likely pathogenic/VUS)	BP1, PS3_mod	VUS	observed in 2 individuals worth 0.5 phenotype points; revised MAF criteria and functional data	BP1, PM2_supp[b]	VUS

(*Continued on next page*)

Table 1. *Continued*

HGVSc	Database ID[a]	Original classification	Criteria applied by algorithm	Classification by algorithm	Further curation	Final criteria applied	Final classification of VCEP
c.3950A>G (p.Glu1317Gly)	ClinVar: 1319598	VUS	BP1, PM2_supp	VUS	observed in 2 individuals worth 0 phenotype points	BP1, PM2_supp	VUS
c.4139C>T (p.Thr1380Ile)	ClinVar: 233890	VUS	BP1, PM2_supp, PS4_supp	VUS	observed in 9 individuals worth 3 phenotype points	BP1, PM2_supp, PS4_mod	VUS
c.4735A>T (p.Ile1579Phe)	ClinVar: 246402	VUS	BP1, PM2_supp, PS3_supp	VUS	observed in 4 individuals worth 1.5 phenotype points	BP1, PM2_supp, PS3_supp PS4_supp	VUS
c.5038C>T (p.Gln1680Ter)	ClinVar: 230520	likely pathogenic	PVS1, BS1	VUS	observed in 1 individual worth 0 phenotype points; revised MAF criteria	PVS1, PM2_supp[b]	likely pathogenic
c.6905C>G (p.Ser2302Ter)	ClinVar: 428166	pathogenic	PVS1	VUS	observed in 1 individual worth 0 phenotype points; revised MAF criteria	PVS1, PM2_supp[b]	likely pathogenic
c.7489_7490insT (p.Ser2497PhefsTer14)	ClinVar: 653103	pathogenic	PVS1, BS1	VUS	observed in 4 individuals worth 1 phenotype point; revised MAF criteria	PVS1, PS4_supp, PM2_supp[b]	pathogenic
c.7798_7801del (p.Gln2600ValfsTer15)	ClinVar: 827255	pathogenic	PVS1, BS1	VUS	observed in 2 individuals worth 0.5 phenotype points; observed in 3 individuals worth 3 healthy individual points; revised MAF criteria	PVS1, PM2_supp[b], BS2_supp	likely pathogenic
c.7803_7807del (p.Ser2601ArgfsTer17)	ClinVar: 648862	likely pathogenic	PVS1	VUS	observed in 1 individual worth 0 phenotype points; revised MAF criteria	PVS1, PM2_supp[b]	likely pathogenic

[a]Database ID from the LOVD has the prefix of "APC_"; database ID from ClinVar are numbers only.
[b]Variants for which the reassessment of minor allele frequency (MAF) criteria where relevant for the final evaluation as LP/P.
[c]This variant seems to be a nonsense variant (p.Arg348Ter) located in exon 10, but RNA analysis (Ambry internal data) resulted in an in-frame aberrant transcript lacking part of exon 10 (r.934_1074del [p.Val312_Gln358del]) and increased expression of a naturally occurring alternative transcript (r.934_1236del [p.Val312_Gln412del], known as "exon 9a") relative to controls. The predicted premature stop codon (p.Arg348Ter) was excluded from both the aberrant and naturally occurring transcripts and might provide a rescue mechanism for this nonsense alteration.

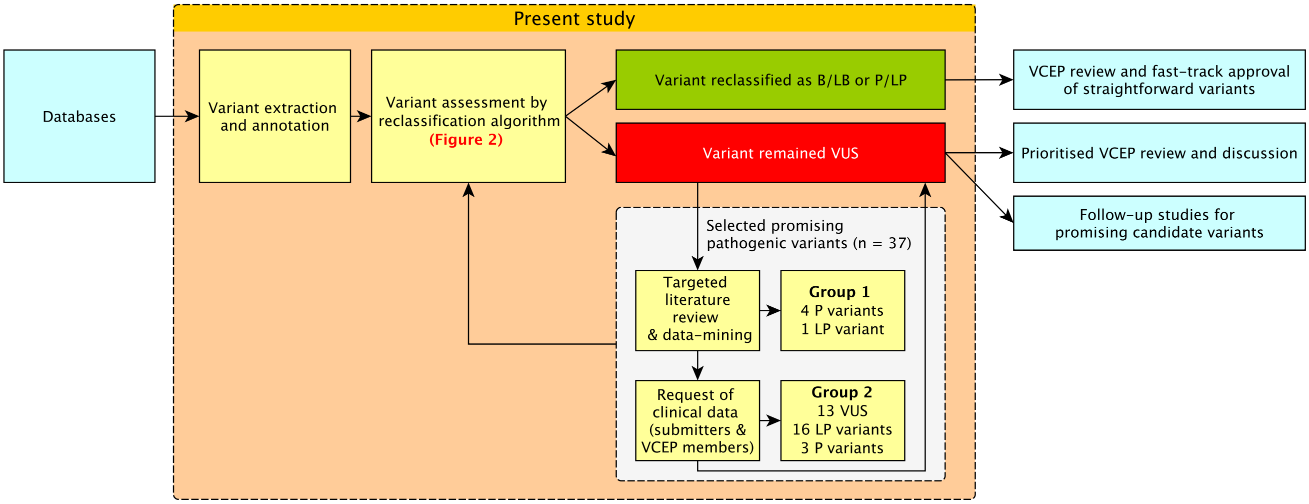

Figure 3. Reclassification workflow and suggested method of operation for ongoing VCEP activity
This workflow summarized the organizational procedures undertaken in this study for variant reclassification and prioritization of variants with high clinical importance for different modes of VCEP review and approval. Relatively straightforward variants might be processed in batch and become candidates for fast-track VCEP approval (e.g., variants fulfilling BA1 or BS1 plus BP1). From the remaining 37 VUSs with some evidence for pathogenicity, five variants were reassessed as P/LP by a targeted literature review and data mining (group 1). The remaining 32 variants were reassessed based on further clinical information requested from respective ClinVar submitters and VCEP members, which ultimately lead to prioritized VCEP review.

dataset in which the B/LB variants represent the largest group (41%) followed by VUS (38%) and P/LP (21%). Notably, 95% of variants with an initial classification of B/LB or P/LP remained in their respective benign and pathogenic categories after reclassification. A considerable portion of previous P variants were downgraded to LP (70%). Remarkably, 41% of previous VUSs were re-classified into clinically relevant pathogenicity classes (40% as B/LB and 1% as P/LP). On the other hand, 86 previously B/LB (4.7%) and 130 previously P/LP variants (5.7%) were reclassified as VUSs, which are summarized in Table S3. Therefore, the total number of VUSs was significantly reduced by 37% from 6,142 to 3,866 through the reclassification process (p value <0.05).

Classification by variant type is shown in Figure 5 with the majority being missense variants (42%) followed by synonymous or intronic variants (25%) and truncating (frameshift/nonsense) variants (19%). The original (O) and revised (R) pathogenicity class was compared for each variant type. In total, the percentage of frameshift/nonsense variants classified as P/LP is the same before and after reclassification (97%). In contrast, the application of the *APC*-specific criteria reduced the percentage of synonymous/intronic (at or beyond +7/−21 intronic positions) VUSs significantly from 38% to 1%, classifying the vast majority as B/LB (99%; p value <0.05). In the original assertions, 99% of putative missense variants are VUSs. This proportion was reduced significantly to 71% using the *APC*-specific criteria, where 29% of all missense variants were reclassified as B/LB (p value <0.05). For variants flanking splice sites (within +7/−21 intronic positions), the range of classifications was similar before and after reclassification. 39% of in-frame, UTR, and other variants were also classified as B/LB, while prior to reclassification they were mostly VUSs (86%). After application of the specific criteria, 3,866 variants remained VUSs, which included 3,067 missense variants (79%) and low numbers of other variant types.

Impact and usage of the *APC*-specific codes and code combinations

The frequency of use of each *APC*-specific code is shown in Table S4A and the codes and code combinations with highest impact on VUS reclassification in Table S4B. All criteria were used at least once during the reclassification process. The most frequently applied codes were the two pathogenic criteria PM2_supporting (69%) and PVS1_variable (21%) and the four benign criteria BP1 (41%), BP4 (27%), BP7 (22%), and BS1 (20%). On the other hand, half of the codes are used for less than 1% of variants. 3,145 variants (31%) were present in the gnomAD v.2.1.1 non-cancer population and/or the UK Biobank non-CRC dataset, of which 2,021 (20%) could be classified as likely benign by the BS1 code alone and 427 (4%) as benign by the BA1 code alone, resulting in a significant reduction of VUSs (p values <0.05 for both). 155 variants (1.5%) were present in the non-CRC control dataset of UK Biobank, which fulfilled the definition for a healthy unaffected individual in the *APC*-specific criteria and allowed the assessment for BS2_variable. The most common code combination overall was BP1 and PM2_supporting resulting in a VUS classification (2,611, 26%). As shown in Table S4B, the most common code combination that led to a non-VUS classification was PVS1 and PM2_supporting, which were applied to 2,138 variants (21%). 80% of these variants (1,707; 17%

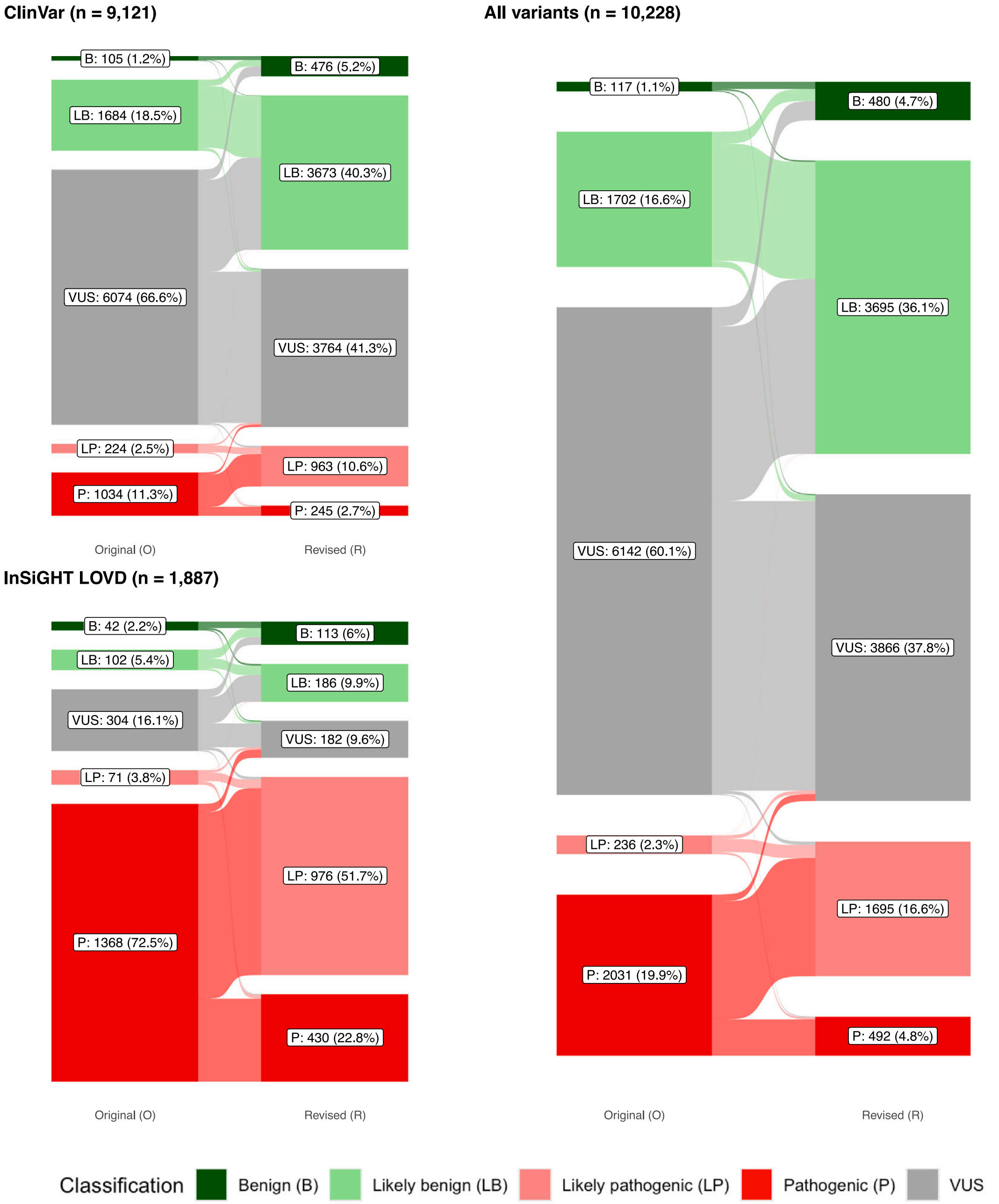

Number of *APC* variants (%) in the combined dataset (ClinVar and InSiGHT LOVD) and their Original (O)and Revised (R) Classifications

	Revised P	Revised LP	Revised VUS	Revised LB	Revised B
Original P	457 (22.5%)	1488 (73.3%)	86 (4.2%)	0 (0%)	0 (0%)
Original LP	25 (10.6%)	166 (70.3%)	44 (18.6%)	1 (0.4%)	0 (0%)
Original VUS	10 (0.2%)	41 (0.7%)	3650 (59.4%)	2200 (35.8%)	241 (3.9%)
Original LB	0 (0%)	0 (0%)	75 (4.4%)	1478 (86.8%)	149 (8.8%)
Original B	0 (0%)	0 (0%)	11 (9.4%)	16 (13.7%)	90 (76.9%)
Total	492 (4.8%)	1695 (16.6%)	3866 (37.8%)	3695 (36.1%)	480 (4.7%)

Figure 4. Classification of all *APC* variants in ClinVar, InSiGHT LOVD, and the combined dataset
Each plot shows the classification change between the original (left) and revised classifications (right) for the *APC* variants in ClinVar (top left), InSiGHT LOVD (bottom left), and the combined dataset (right). The bottom table shows the number of *APC* variants (%) in the combined dataset (ClinVar and InSiGHT LOVD) and their original and revised classifications.

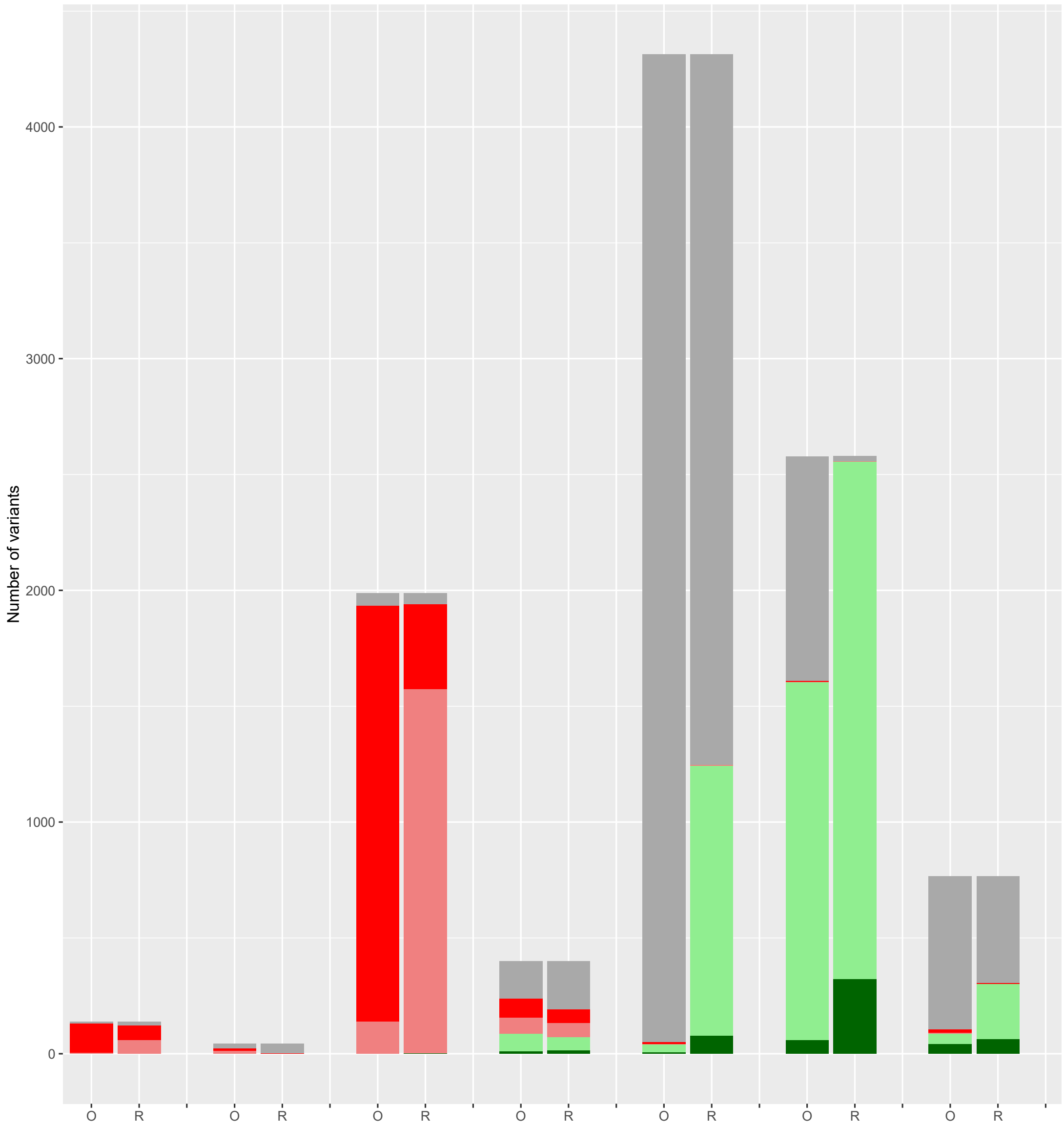

Classification	Gross Deletion O	Gross Deletion R	Gross Duplication O	Gross Duplication R	Frameshift/Nonsense O	Frameshift/Nonsense R	Splice Site O	Splice Site R	Missense O	Missense R	Intronic/Synonymous O	Intronic/Synonymous R	Inframe/UTR/Other O	Inframe/UTR/Other R
B	0 (0.0%)	0 (0.0%)	0 (0.0%)	0 (0.0%)	0 (0.0%)	1 (0.1%)	10 (2.5%)	14 (3.5%)	6 (0.1%)	79 (1.8%)	59 (2.3%)	323 (12.5%)	42 (5.5%)	64 (8.3%)
LB	0 (0.0%)	0 (0.0%)	1 (2.3%)	0 (0.0%)	0 (0.0%)	2 (0.1%)	77 (19.3%)	59 (14.8%)	34 (0.8%)	1164 (27.0%)	1545 (59.9%)	2232 (86.5%)	45 (5.9%)	237 (30.9%)
VUS	8 (5.8%)	16 (11.6%)	21 (47.7%)	42 (95.5%)	55 (2.8%)	49 (2.5%)	161 (40.4%)	208 (52.1%)	4263 (98.8%)	3067 (71.1%)	971 (37.7%)	22 (0.9%)	663 (86.4%)	461 (60.1%)
LP	5 (3.6%)	60 (43.5%)	12 (27.3%)	0 (0.0%)	140 (7.0%)	1570 (79.0%)	71 (17.8%)	60 (15.0%)	3 (0.1%)	3 (0.1%)	1 (0.0%)	2 (0.1%)	4 (0.5%)	1 (0.1%)
P	125 (90.6%)	62 (44.9%)	10 (22.7%)	2 (4.5%)	1793 (90.2%)	366 (18.4%)	80 (20.1%)	58 (14.5%)	7 (0.2%)	0 (0.0%)	3 (0.1%)	0 (0.0%)	13 (1.7%)	4 (0.5%)
Total	138 (100.0%)	138 (100.0%)	44 (100.0%)	44 (100.0%)	1988 (100.0%)	1988 (100.0%)	399 (100.0%)	399 (100.0%)	4313 (100.0%)	4313 (100.0%)	2579 (100.0%)	2579 (100.0%)	767 (100.0%)	767 (100.0%)

Figure 5. Classification of all *APC* variants in the original database (O) and their revised classification (R) by variant type
Variants are broadly categorized into seven categories: 138 gross deletions, 44 gross duplications, 1,988 frameshift/nonsense, 399 splice site, 4,313 missense, 2,579 intronic/synonymous, and 767 other variants (120 in-frame, 631 UTR, and 16 other variants, which included start-loss, stop-loss, stop-retained, Alu and SVA retrotransposon insertions, inversions, and complex variants).

of all variants) were classified as LP without the need of clinical information, and 20% (431; 4% of all variants) were classified as P together with variable other codes (e.g., PS4_variable). This was also the most efficient code combination to reclassify VUSs into a pathogenic (LP/P) class, although the number is small (0.7% reduction of all VUSs; p value <0.05) since only very few truncating variants were previously classified as VUSs.

As expected, the allele frequency threshold criterion BS1 was most frequently applied and has the highest impact on shifts from VUS into a (L)B classification (25% reduction of all VUSs; p value < 0.05). In addition, the codes BP4 and BP7 for intronic and synonymous variants without splice effect had a significant impact on VUS reduction (11% reduction of all VUSs; p value < 0.05).

Further data mining and criteria review for a prioritized list of variants

After the initial application of the *APC*-specific criteria, a considerable fraction of VUSs (63%) remained as expected. We selected 37 promising variants from these remaining VUSs with some evidence for pathogenicity (for details, see methods), which formed a prioritized list of variants for further review as outlined in the workflow (Figure 3).

In 11 variants for which PVS1 is applicable (Table 1, encompassing both group 1 and 2), the relegation of their prior LP/P classification was due to their presence at very low frequencies in reference population databases, in this case the occurrence of one allele in a gnomAD non-cancer subpopulation. Depending on the denominator (i.e., size of the subpopulation), seven previously LP/P truncating variants were precluded from the use of PM2_supporting, and four even fulfilled threshold for BS1 using version 1.0.0 of the *APC*-specific criteria. To resolve this issue, the *APC* VCEP added a caveat to PM2_supporting in the criteria version 2.1.0 where the allele frequency threshold of ≤0.0003% (0.000003) is only used if the allele count is >1. To tolerate singleton allele occurrence in gnomAD, the *APC* VCEP set an allele frequency of <0.001% (0.00001) (lower than BS1) if the allele count is ≤1. Moreover, the *APC* VCEP recommended in criteria version 2.1.0 the use of the filtering allele frequency (FAF) for BA1 and BS1 to avoid the issue of singleton alleles satisfying the allele frequency criteria. This allowed the use of PM2_supporting and the reclassification of these 11 variants as LP/P.

For five variants that were originally P/LP in ClinVar or LOVD but reclassified as VUSs by the algorithm, a more extensive data mining and literature review led to the return of P/LP as their final classification (group 1). The previous pathogenic classification would indicate the observation of these variants in affected individuals on multiple occasions, and a targeted search was finally informative.

For the remaining 32 variants (group 2) further phenotype data was requested from VCEP members, given their persistent VUS status despite additional curation of the literature. This allowed the upgrade of classification from VUS to P/LP for 11 (34%) variants (group 2). Five variants of group 2 were evaluated as LP based on the reassessment of the MAF criteria, but no relevant phenotypic information was available. Overall, further data mining of selected representative variants resulted in the enhanced classification of 24 out of 37 variants (65%) into meaningful pathogenicity classes.

Discussion

The rising number of VUSs in clinically actionable genes such as *APC* represents an important issue in the post-genomic era that hinders the translation of genetic diagnostics into clinical practice. In this study, we first identified the current landscape of *APC* variants in two of the most prominent international databases, namely ClinVar and the InSiGHT LOVD, and then applied the full set of ClinGen-approved, gene-specific ACMG/AMP criteria with the aim to improve consistency and accuracy in *APC* variant classification for >10,000 variants. A striking difference was noted in the distribution of pathogenicity classes: while around two-thirds of ClinVar variants were originally VUSs and 20% B/LB, approximately 75% of *APC* variants on LOVD were P/LP (Figure 4). This is not unexpected since variants submitted to LOVD are usually detected in individuals with the relevant phenotype (i.e., clinically evident colorectal adenomatous polyposis) where the detection of PVs is more likely. In contrast, variants in ClinVar are mostly derived from high-throughput sequencing approaches in individuals with less specific or unrelated phenotypes (e.g., multigene hereditary cancer panel testing in patients having testing for reasons other than a history of adenomatous polyposis) and healthy individuals. Consequently, the majority of these variants is expected to be benign or have a low penetrance. However, in the absence of overwhelming evidence, they are usually conservatively classified as VUSs.

One of the major findings of this study is that the application of the *APC*-specific criteria reduced the number of VUSs by 41% collectively in ClinVar and LOVD, the majority (40%) were reclassified as B/LB (n = 2,441) owing to their presence in reference population databases fulfilling MAF criteria (BA1 or BS1) or no predicted impact on splicing (BP4 + BP7). Their benign classification in turn alleviates anxiety and potential overtreatment in affected individuals worldwide.[60] On the other hand, 51 previous VUSs were reclassified as P/LP (0.8%), a result which confirms the diagnosis of FAP and enables the timely management and predictive testing of all at-risk relatives. Finally, 95% and 94% of the previously B/LB and P/LP variants remained in their concordant classifications. These findings demonstrate that the application of the *APC*-specific criteria is highly effective in improving the reclassification of VUSs into clinically relevant pathogenicity classes while preserving the original interpretation of variants with existing evidence-based classifications.

In the combined databases, a total of 130 P/LP variants (93 non-structural, 37 structural) and 86 B/LB variants (85 structural, 1 non-structural) were reclassified as VUSs (216 in total, 5% of previous non-VUSs), which is clinically significant for the previous P/LP variants and will affect the diagnosis, counseling, and predictive testing (Table S3). Among the 130 P/LP-to-VUS variants, 14 were gross deletions, affecting only the promoter region and/or the first coding exon, and 21 gross duplications which had unknown impact on the reading frame, even though many *APC* gross duplications are indeed located in a tandem position.[4,61,62] In the absence of convincing clinical data, these gross deletions and duplications could not be classified as LP/P. Sixteen truncating variants at the 5′ or 3′ end of the gene were excluded from the application of PVS1 and were classified as VUSs. We also noted that canonical ±1/2 splice site changes and intronic variants flanking the splice sites (+7/−21 bp) were mostly deemed LP/P by LOVD and ClinVar submitters (43/62 variants [69%] that were reclassified as VUSs), although splice predictions and transcript analyses might have suggested otherwise (i.e., weak native site, alternative transcripts, etc.). On the other hand, RNA analysis data supporting a splice effect were mainly not available for this large-scale project. The remaining variants included 55 deep intronic, synonymous, in-frame, and UTR variants, as well as 25 missense variants, where the reclassification as VUS was the appropriate result of a combination of scarcity of clinical data and non-contributory *in silico* predictions.

In the *APC*-specific criteria, a range of evidence weight adjustments is specified as a means of improving precision and quality. The lack of detailed evidence descriptions in databases and publications meant that certain criteria can only be applied at lower weights, even though ClinVar and LOVD submitters may have additional information, especially the phenotype (including segregation) and functional analyses. Since this information is either not publicly available in the databases or is elaborate to extract from publications, the clinical-data-driven criteria are only applied at the end of the reclassification algorithm (Figure 2). In this study, clinical data were only extracted from individuals with phenotype data in LOVD. For a selected number of promising variants ($n = 37$; Table 1), additional data mining and a request among our VCEP members for clinical and if necessary for RNA analysis data was carried out. 65% of these variants could again be reclassified into P/LP, demonstrating the relevance of further clinical and RNA analysis data.

A considerable number of previous P variants were downgraded to LP (70%) (e.g., truncating variants fulfilling only PVS1 and PM2_supporting)—a result also known from other genes where gene-specific rules are being applied. Submitters may be overrating the quality of phenotype data, have additional data, or were applying the original moderate strength for PM2. Consequently, it seems likely that some reclassified LP variants are in fact P, which has the potential for upgrade through diligent reporting of clinical information and data sharing. In practical terms, an LP classification has a posterior probability of pathogenicity of 0.9–0.99, which nonetheless demands clinical action when detected.[63,64]

After reclassification, 80% of the remaining VUSs (3,067/3,866) were presumed missense variants, which don't meet the allele frequency thresholds for BA1 and BS1. As discussed previously,[24,34] true LP/P missense variants are extremely rare in *APC* since the fundamental mechanism of *APC* pathogenicity is based on the loss of a large C-terminal part of the protein which includes the relevant functional domains. In addition to further evidence of functional redundancy in the APC protein, the central and C-terminal domains of the APC protein are natively unfolded, which likely explains its resistance to missense variation.[65] Consequently, the vast majority of the remaining missense *APC* VUSs in ClinVar are presumably benign incidental findings from non-targeted testing in individuals with unrelated phenotypes, although this is challenging to verify since *in silico* prediction tools are not applicable for *APC* missense variants.[24] Similarly, massively parallel functional assays on protein function are unlikely to contribute significantly to improve classifications. Interpreting a missense *APC* variant as benign is therefore heavily dependent on the clinical description of the associated individual (a lack of CRC/polyposis phenotype), in which case BS2_supporting could be applied in conjunction with BP1 to result in an LB classification. The recruitment of variant data from large reference population projects such as the UK Biobank is another option to determine the pathogenicity of these missense variants.

This reclassification endeavor was also considered a proof-of-concept study for the ongoing method of operation of VCEPs (Figure 3). The review and discussion of every single submitted constitutional variant in *APC* is unrealistic with respect to the resources currently provided to a VCEP until appropriate bioinformatic tools become available in the future. In this study, we developed a stratified variant curation process whereby the relatively straightforward variants could be processed in batch and become candidates for fast-track VCEP review (e.g., variants fulfilling BA1 or BS1 plus BP1 without any conflicting classifications submitted to ClinVar or LOVD) in an updated variant curation interface (VCI) to streamline the variant approval process. We identified several prioritized groups of variants that can be the subject of further targeted literature review (Table S3), data-mining, and clinical data requests from database submitters and VCEP members, a process to enhance variant interpretation as demonstrated in this study. These challenging variants can also form the basis for scientific follow-up studies to evaluate the causal relationships using additional investigations such as segregation or transcript analyses.

Variants that are listed as "conflicting" in ClinVar require special consideration by the VCEPs. However, there are only few *APC* variants in ClinVar with clinically significant

conflict (defined as variant with concomitant VUS and P/LP classifications), including variants with different interpretations of splicing effects, truncating variants upstream or downstream of the PVS1 boundaries and few missense variants. Some of these variants have been already assessed by the VCEP or are prioritized for further expert curation and panel review. As per the ClinVar assertion process, variants fully classified by the VCEP will appear with a three-star pathogenicity assertion that overrules the other submissions and therefore will no longer be conflicting. A special case are the two widely known low-penetrant variants c.3920T>A (p.Ile1307Lys) and c.3949G>C (p.Glu1317Gln), which are listed as conflicting in ClinVar. As the ACMG/AMP rules were designed for a Mendelian inheritance model, they cannot be applied to such variants.[24] The current evidence of the p.Ile307Lys variant was recently summarized,[66] and the ClinGen Low Penetrance/Risk Allele Working Group has published recommendations for how these variants should be reported.[67]

In this study, we developed and applied an algorithm for large-scale variant reclassification and demonstrated that the application of *APC*-specific criteria can substantially alleviate the burden of VUSs in ClinVar and LOVD, thereby laying the groundwork for a prospective streamlined expert panel approval of clinically actionable *APC* variants in the VCI. By using the VCEP specifications, diagnostic laboratories could reduce their rates of reporting VUSs. Previous studies for other genes were either smaller, used gene- or disease-specifications that covered only partial evidence domains,[68] or meta-classification methods such as the multifactorial likelihood analysis.[69,70] The present study highlights the utility of a systematic, data-driven analysis using gene-specific ACMG/AMP criteria, complemented by further targeted data-mining and clinical data requests. By this approach, this study marks the initiation of a dynamic, long-term curation process for the *APC* VCEP. The suggested workflow can serve as a generalizable model of operation for other gene- or disease-specific variant interpretation initiatives, achieving accurate and highly efficient variant interpretation based on an array of carefully curated evidence.

To further improve VUS interpretation and provide clinically informative variant classification beyond this approach, the availability of more population-based datasets and user-friendly modes of sharing clinical and molecular data are needed; a challenge that needs to be addressed by the respective expert communities and data submitters.

Data and code availability

All data supporting the findings and conclusions and all significant results generated during this study are available within the published article and the supplemental information. All variants reviewed and reclassified by the ClinGen-InSiGHT Variant Curation Expert Panel in this study have been submitted to the ClinVar Database. The detailed evidence used for the classification of these variants is available in the ClinGen Evidence Repository (https://erepo.clinicalgenome.org/evrepo/). These data and all internal data cited in this manuscript may also become available upon a data transfer agreement approved by the local ethics committee and can be obtained after contacting the corresponding author (S.A.) upon request.

Acknowledgments

This publication was supported in part by the National Human Genome Research Institute of the National Institutes of Health for the Baylor College of Medicine/Stanford University Clinical Genome Resource-2U24HG009649 and from the National Cancer Institute U24 Curation Panels through U24CA258119. This work was supported (not financially) by the European Reference Network on Genetic Tumour Risk Syndromes (ERN GENTURIS): project ID no. 739547. ERN GENTURIS is partly co-funded by the European Union within the framework of the Third Health Programme "ERN-2016—Framework Partnership Agreement 2017–2021." We thank John Ranola for his support and guidance in statistical analyses.

Declaration of interests

S.E.P. is a member of the scientific advisory panel of Baylor Genetics Laboratories.

Supplemental information

Supplemental information can be found online at https://doi.org/10.1016/j.ajhg.2024.09.002.

Web resources

ClinGen (Clinical Genome Resource), www.clinicalgenome.org

ClinGen-InSiGHT Hereditary Colorectal Cancer/Polyposis Variant Curation Expert Panel, https://www.clinicalgenome.org/affiliation/50099/

Criteria Specification Registry, https://cspec.genome.network/cspec/ui/svi/

ClinVar, https://www.ncbi.nlm.nih.gov/clinvar/

Ensembl Variant Effect Predictor, https://asia.ensembl.org/info/docs/tools/vep/index.html

Global Variome shared Leiden Open Variation Database (LOVD) https://www.lovd.nl/apc; https://databases.lovd.nl/shared/genes/APC

HGVS (Human Genome Variation Society), https://varnomen.hgvs.org/

InSiGHT (International Society for Gastrointestinal Hereditary Tumours), https://www.insight-group.org/

InSiGHT *APC* LOVD, https://www.insight-database.org/genes/APC

MaxEntScan for 5′ sites, http://hollywood.mit.edu/burgelab/maxent/Xmaxentscan_scoreseq.html

MaxEntScan for 3′ sites, http://hollywood.mit.edu/burgelab/maxent/Xmaxentscan_scoreseq_acc.html

OMIM (Online Mendelian Inheritance in Man), https://www.omim.org

SpliceAI, https://spliceailookup.broadinstitute.org/

Received: April 14, 2024
Accepted: September 6, 2024
Published: October 1, 2024

References

1. Kinzler, K.W., Nilbert, M.C., Su, L.K., Vogelstein, B., Bryan, T.M., Levy, D.B., Smith, K.J., Preisinger, A.C., Hedge, P., and McKechnie, D. (1991). Identification of FAP locus genes from chromosome 5q21. Science *253*, 661–665.
2. Groden, J., Thliveris, A., Samowitz, W., Carlson, M., Gelbert, L., Albertsen, H., Joslyn, G., Stevens, J., Spirio, L., and Robertson, M. (1991). Identification and characterization of the familial adenomatous polyposis coli gene. Cell *66*, 589–600.
3. WHO (2024). The Introduction to Genetic Tumour Syndromes. In Genetic Tumour Syndromes 5th ed, B. WCoTE, ed.
4. Jasperson, K.W., Tuohy, T.M., Neklason, D.W., and Burt, R.W. (2010). Hereditary and familial colon cancer. Gastroenterology *138*, 2044–2058.
5. Syngal, S., Brand, R.E., Church, J.M., Giardiello, F.M., Hampel, H.L., Burt, R.W.; and American College of Gastroenterology (2015). ACG clinical guideline: Genetic testing and management of hereditary gastrointestinal cancer syndromes. Am. J. Gastroenterol. *110*, 223–263.
6. Monahan, K.J., Bradshaw, N., Dolwani, S., Desouza, B., Dunlop, M.G., East, J.E., Ilyas, M., Kaur, A., Lalloo, F., Latchford, A., et al. (2020). Guidelines for the management of hereditary colorectal cancer from the British Society of Gastroenterology (BSG)/Association of Coloproctology of Great Britain and Ireland (ACPGBI)/United Kingdom Cancer Genetics Group (UKCGG). Gut *69*, 411–444.
7. van Leerdam, M.E., Roos, V.H., van Hooft, J.E., Dekker, E., Jover, R., Kaminski, M.F., Latchford, A., Neumann, H., Pellisé, M., Saurin, J.C., et al. (2019). Endoscopic management of polyposis syndromes: European Society of Gastrointestinal Endoscopy (ESGE) Guideline. Endoscopy *51*, 877–895.
8. Miller, D.T., Lee, K., Abul-Husn, N.S., Amendola, L.M., Brothers, K., Chung, W.K., Gollob, M.H., Gordon, A.S., Harrison, S.M., Hershberger, R.E., et al. (2022). ACMG SF v3.1 list for reporting of secondary findings in clinical exome and genome sequencing: A policy statement of the American College of Medical Genetics and Genomics (ACMG). Genet. Med. *24*, 1407–1414.
9. van der Luijt, R.B., Khan, P.M., Vasen, H.F., Tops, C.M., van Leeuwen-Cornelisse, I.S., Wijnen, J.T., van der Klift, H.M., Plug, R.J., Griffioen, G., and Fodde, R. (1997). Molecular analysis of the APC gene in 105 Dutch kindreds with familial adenomatous polyposis: 67 germline mutations identified by DGGE, PTT, and southern analysis. Hum. Mutat. *9*, 7–16.
10. Wallis, Y.L., Morton, D.G., McKeown, C.M., and Macdonald, F. (1999). Molecular analysis of the APC gene in 205 families: extended genotype-phenotype correlations in FAP and evidence for the role of APC amino acid changes in colorectal cancer predisposition. J. Med. Genet. *36*, 14–20.
11. Friedl, W., Caspari, R., Sengteller, M., Uhlhaas, S., Lamberti, C., Jungck, M., Kadmon, M., Wolf, M., Fahnenstich, J., Gebert, J., et al. (2001). Can APC mutation analysis contribute to therapeutic decisions in familial adenomatous polyposis? Experience from 680 FAP families. Gut *48*, 515–521.
12. Bisgaard, M.L., Ripa, R., Knudsen, A.L., and Bülow, S. (2004). Familial adenomatous polyposis patients without an identified APC germline mutation have a severe phenotype. Gut *53*, 266–270.
13. Rivera, B., González, S., Sánchez-Tomé, E., Blanco, I., Mercadillo, F., Letón, R., Benítez, J., Robledo, M., Capellá, G., and Urioste, M. (2011). Clinical and genetic characterization of classical forms of familial adenomatous polyposis: a Spanish population study. Ann. Oncol. *22*, 903–909.
14. Vasen, H.F.A., Möslein, G., Alonso, A., Aretz, S., Bernstein, I., Bertario, L., Blanco, I., Bülow, S., Burn, J., Capella, G., et al. (2008). Guidelines for the clinical management of familial adenomatous polyposis (FAP). Gut *57*, 704–713.
15. Jarvik, G.P., and Browning, B.L. (2016). Consideration of Cosegregation in the Pathogenicity Classification of Genomic Variants. Am. J. Hum. Genet. *98*, 1077–1081.
16. Abou Tayoun, A.N., Pesaran, T., DiStefano, M.T., Oza, A., Rehm, H.L., Biesecker, L.G., Harrison, S.M.; and ClinGen Sequence Variant Interpretation Working Group ClinGen SVI (2018). Recommendations for interpreting the loss of function PVS1 ACMG/AMP variant criterion. Hum. Mutat. *39*, 1517–1524.
17. Biesecker, L.G., Harrison, S.M.; and ClinGen Sequence Variant Interpretation Working Group (2018). The ACMG/AMP reputable source criteria for the interpretation of sequence variants. Genet. Med. *20*, 1687–1688.
18. Ghosh, R., Harrison, S.M., Rehm, H.L., Plon, S.E., Biesecker, L.G.; and ClinGen Sequence Variant Interpretation Working Group (2018). Updated recommendation for the benign stand-alone ACMG/AMP criterion. Hum. Mutat. *39*, 1525–1530.
19. Brnich, S.E., Abou Tayoun, A.N., Couch, F.J., Cutting, G.R., Greenblatt, M.S., Heinen, C.D., Kanavy, D.M., Luo, X., McNulty, S.M., Starita, L.M., et al. (2019). Recommendations for application of the functional evidence PS3/BS3 criterion using the ACMG/AMP sequence variant interpretation framework. Genome Med. *12*, 3.
20. Rofes, P., Menéndez, M., González, S., Tornero, E., Gómez, C., Vargas-Parra, G., Montes, E., Salinas, M., Solanes, A., Brunet, J., et al. (2020). Improving Genetic Testing in Hereditary Cancer by RNA Analysis: Tools to Prioritize Splicing Studies and Challenges in Applying American College of Medical Genetics and Genomics Guidelines. J. Mol. Diagn. *22*, 1453–1468.
21. Smirnov, D., Schlieben, L.D., Peymani, F., Berutti, R., and Prokisch, H. (2022). Guidelines for clinical interpretation of variant pathogenicity using RNA phenotypes. Hum. Mutat. *43*, 1056–1070.
22. Rivera-Muñoz, E.A., Milko, L.V., Harrison, S.M., Azzariti, D.R., Kurtz, C.L., Lee, K., Mester, J.L., Weaver, M.A., Currey, E., Craigen, W., et al. (2018). ClinGen Variant Curation Expert Panel experiences and standardized processes for disease and gene-level specification of the ACMG/AMP guidelines for sequence variant interpretation. Hum. Mutat. *39*, 1614–1622.
23. Plazzer, J.P., Sijmons, R.H., Woods, M.O., Peltomäki, P., Thompson, B., Den Dunnen, J.T., and Macrae, F. (2013). The InSiGHT database: utilizing 100 years of insights into Lynch syndrome. Fam. Cancer *12*, 175–180.
24. Spier, I., Yin, X., Richardson, M., Pineda, M., Laner, A., Ritter, D., Boyle, J., Mur, P., Hansen, T.V.O., Shi, X., et al. (2024). Gene-specific ACMG/AMP classification criteria for germline APC variants: Recommendations from the ClinGen InSiGHT Hereditary Colorectal Cancer/Polyposis Variant Curation Expert Panel. Genet. Med. *26*, 100992.

25. den Dunnen, J.T., Dalgleish, R., Maglott, D.R., Hart, R.K., Greenblatt, M.S., McGowan-Jordan, J., Roux, A.F., Smith, T., Antonarakis, S.E., and Taschner, P.E.M. (2016). HGVS Recommendations for the Description of Sequence Variants: 2016 Update. Hum. Mutat. *37*, 564–569.
26. McLaren, W., Gil, L., Hunt, S.E., Riat, H.S., Ritchie, G.R.S., Thormann, A., Flicek, P., and Cunningham, F. (2016). The Ensembl Variant Effect Predictor. Genome Biol. *17*, 122.
27. Lefter, M., Vis, J.K., Vermaat, M., den Dunnen, J.T., Taschner, P.E.M., and Laros, J.F.J. (2021). Mutalyzer 2: next generation HGVS nomenclature checker. Bioinformatics *37*, 2811–2817.
28. Richards, S., Aziz, N., Bale, S., Bick, D., Das, S., Gastier-Foster, J., Grody, W.W., Hegde, M., Lyon, E., Spector, E., et al. (2015). Standards and guidelines for the interpretation of sequence variants: a joint consensus recommendation of the American College of Medical Genetics and Genomics and the Association for Molecular Pathology. Genet. Med. *17*, 405–424.
29. Karczewski, K.J., Francioli, L.C., Tiao, G., Cummings, B.B., Alföldi, J., Wang, Q., Collins, R.L., Laricchia, K.M., Ganna, A., Birnbaum, D.P., et al. (2020). The mutational constraint spectrum quantified from variation in 141,456 humans. Nature *581*, 434–443.
30. Sudlow, C., Gallacher, J., Allen, N., Beral, V., Burton, P., Danesh, J., Downey, P., Elliott, P., Green, J., Landray, M., et al. (2015). UK biobank: an open access resource for identifying the causes of a wide range of complex diseases of middle and old age. PLoS Med. *12*, e1001779.
31. MacDonald, J.R., Ziman, R., Yuen, R.K.C., Feuk, L., and Scherer, S.W. (2014). The Database of Genomic Variants: a curated collection of structural variation in the human genome. Nucleic Acids Res. *42*, D986–D992.
32. Jaganathan, K., Kyriazopoulou Panagiotopoulou, S., McRae, J.F., Darbandi, S.F., Knowles, D., Li, Y.I., Kosmicki, J.A., Arbelaez, J., Cui, W., Schwartz, G.B., et al. (2019). Predicting Splicing from Primary Sequence with Deep Learning. Cell *176*, 535–548.e24.
33. Yeo, G., and Burge, C.B. (2004). Maximum entropy modeling of short sequence motifs with applications to RNA splicing signals. J. Comput. Biol. *11*, 377–394.
34. Karabachev, A.D., Martini, D.J., Hermel, D.J., Solcz, D., Richardson, M.E., Pesaran, T., Sarkar, I.N., and Greenblatt, M.S. (2020). Curated multiple sequence alignment for the Adenomatous Polyposis Coli (APC) gene and accuracy of in silico pathogenicity predictions. PLoS One *15*, e0233673.
35. Stenson, P.D., Ball, E.V., Mort, M., Phillips, A.D., Shiel, J.A., Thomas, N.S.T., Abeysinghe, S., Krawczak, M., and Cooper, D.N. (2003). Human Gene Mutation Database (HGMD): 2003 update. Hum. Mutat. *21*, 577–581.
36. Béroud, C., Collod-Béroud, G., Boileau, C., Soussi, T., and Junien, C. (2000). UMD (Universal mutation database): a generic software to build and analyze locus-specific databases. Hum. Mutat. *15*, 86–94.
37. Moser, A.R., Luongo, C., Gould, K.A., McNeley, M.K., Shoemaker, A.R., and Dove, W.F. (1995). ApcMin: a mouse model for intestinal and mammary tumorigenesis. Eur. J. Cancer *31a*, 1061–1064.
38. Crobach, S., van Wezel, T., Vasen, H.F., and Morreau, H. (2012). Ovarian metastases of colorectal and duodenal cancer in familial adenomatous polyposis. Fam. Cancer *11*, 671–673.
39. Ruys, A.T., Alderlieste, Y.A., Gouma, D.J., Dekker, E., and Mathus-Vliegen, E.M.H. (2010). Jejunal cancer in patients with familial adenomatous polyposis. Clin. Gastroenterol. Hepatol. *8*, 731–733.
40. Spirio, L., Olschwang, S., Groden, J., Robertson, M., Samowitz, W., Joslyn, G., Gelbert, L., Thliveris, A., Carlson, M., and Otterud, B. (1993). Alleles of the APC gene: an attenuated form of familial polyposis. Cell *75*, 951–957.
41. Nasioulas, S., Jones, I.T., St John, D.J., Scott, R.J., Forrest, S.M., and McKinlay Gardner, R.J. (2001). Profuse familial adenomatous polyposis with an adenomatous polyposis coli exon 3 mutation. Fam. Cancer *1*, 3–7.
42. Wanitsuwan, W., Vijasika, S., Jirarattanasopa, P., and Horpaopan, S. (2021). A distinct APC pathogenic germline variant identified in a southern Thai family with familial adenomatous polyposis. BMC Med. Genomics *14*, 87.
43. Ercoskun, P., Yuce Kahraman, C., Ozkan, G., and Tatar, A. (2022). Genetic Characterization of Hereditary Cancer Syndromes Based on Targeted Next-Generation Sequencing. Mol. Syndromol. *13*, 123–131.
44. Filipe, B., Baltazar, C., Albuquerque, C., Fragoso, S., Lage, P., Vitoriano, I., Mão de Ferro, S., Claro, I., Rodrigues, P., Fidalgo, P., et al. (2009). APC or MUTYH mutations account for the majority of clinically well-characterized families with FAP and AFAP phenotype and patients with more than 30 adenomas. Clin. Genet. *76*, 242–255.
45. De Rosa, M., Dourisboure, R.J., Morelli, G., Graziano, A., Gutiérrez, A., Thibodeau, S., Halling, K., Avila, K.C., Duraturo, F., Podesta, E.J., et al. (2004). First genotype characterization of Argentinean FAP patients: identification of 14 novel APC mutations. Hum. Mutat. *23*, 523–524.
46. Latchford, A., Volikos, E., Johnson, V., Rogers, P., Suraweera, N., Tomlinson, I., Phillips, R., and Silver, A. (2007). APC mutations in FAP-associated desmoid tumours are non-random but not 'just right'. Hum. Mol. Genet. *16*, 78–82.
47. Yurgelun, M.B., Kulke, M.H., Fuchs, C.S., Allen, B.A., Uno, H., Hornick, J.L., Ukaegbu, C.I., Brais, L.K., McNamara, P.G., Mayer, R.J., et al. (2017). Cancer Susceptibility Gene Mutations in Individuals With Colorectal Cancer. J. Clin. Oncol. *35*, 1086–1095.
48. Kanter-Smoler, G., Fritzell, K., Rohlin, A., Engwall, Y., Hallberg, B., Bergman, A., Meuller, J., Grönberg, H., Karlsson, P., Björk, J., and Nordling, M. (2008). Clinical characterization and the mutation spectrum in Swedish adenomatous polyposis families. BMC Med. *6*, 10.
49. Aretz, S., Uhlhaas, S., Sun, Y., Pagenstecher, C., Mangold, E., Caspari, R., Möslein, G., Schulmann, K., Propping, P., and Friedl, W. (2004). Familial adenomatous polyposis: aberrant splicing due to missense or silent mutations in the APC gene. Hum. Mutat. *24*, 370–380.
50. Friedl, W., and Aretz, S. (2005). Familial adenomatous polyposis: experience from a study of 1164 unrelated german polyposis patients. Hered. Cancer Clin. Pract. *3*, 95–114.
51. Li, M., Gerber, D.A., Koruda, M., and O'Neil, B.H. (2012). Hepatocelluar carcinoma associated with attenuated familial adenomatous polyposis: a case report and review of the literature. Clin. Colorectal Cancer *11*, 77–81.
52. Yurgelun, M.B., Allen, B., Kaldate, R.R., Bowles, K.R., Judkins, T., Kaushik, P., Roa, B.B., Wenstrup, R.J., Hartman, A.R., and Syngal, S. (2015). Identification of a Variety of Mutations in Cancer Predisposition Genes in Patients With Suspected Lynch Syndrome. Gastroenterology *149*, 604–613.e20.
53. Susswein, L.R., Marshall, M.L., Nusbaum, R., Vogel Postula, K.J., Weissman, S.M., Yackowski, L., Vaccari, E.M.,

Bissonnette, J., Booker, J.K., Cremona, M.L., et al. (2016). Pathogenic and likely pathogenic variant prevalence among the first 10,000 patients referred for next-generation cancer panel testing. Genet. Med. *18*, 823–832.
54. Ficari, F., Cama, A., Valanzano, R., Curia, M.C., Palmirotta, R., Aceto, G., Esposito, D.L., Crognale, S., Lombardi, A., Messerini, L., et al. (2000). APC gene mutations and colorectal adenomatosis in familial adenomatous polyposis. Br. J. Cancer *82*, 348–353.
55. Lagarde, A., Rouleau, E., Ferrari, A., Noguchi, T., Qiu, J., Briaux, A., Bourdon, V., Rémy, V., Gaildrat, P., Adélaïde, J., et al. (2010). Germline APC mutation spectrum derived from 863 genomic variations identified through a 15-year medical genetics service to French patients with FAP. J. Med. Genet. *47*, 721–722.
56. Gavert, N., Yaron, Y., Naiman, T., Bercovich, D., Rozen, P., Shomrat, R., Legum, C., and Orr-Urtreger, A. (2002). Molecular analysis of the APC gene in 71 Israeli families: 17 novel mutations. Hum. Mutat. *19*, 664.
57. Davidson, S., Leshanski, L., Rennert, G., Eidelman, S., and Amikam, D. (2002). Maternal mosaicism for a second mutational event–a novel deletion–in a familial adenomatous polyposis family harboring a new germ-line mutation in the alternatively spliced-exon 9 region of APC. Hum. Mutat. *19*, 83–84.
58. Tsukanov, A.S., Pospekhova, N.I., Shubin, V.P., Kuzminov, A.M., Kashnikov, V.N., Frolov, S.A., and Shelygin, Y.A. (2017). Mutations in the APC gene in Russian patients with classic form of familial adenomatous polyposis. Russ. J. Genet. *53*, 369–375.
59. Spier, I., Horpaopan, S., Vogt, S., Uhlhaas, S., Morak, M., Stienen, D., Draaken, M., Ludwig, M., Holinski-Feder, E., Nöthen, M.M., et al. (2012). Deep intronic APC mutations explain a substantial proportion of patients with familial or early-onset adenomatous polyposis. Hum. Mutat. *33*, 1045–1050.
60. Welsh, J.L., Hoskin, T.L., Day, C.N., Thomas, A.S., Cogswell, J.A., Couch, F.J., and Boughey, J.C. (2017). Clinical Decision-Making in Patients with Variant of Uncertain Significance in BRCA1 or BRCA2 Genes. Ann. Surg Oncol. *24*, 3067–3072.
61. Kaufmann, A., Vogt, S., Uhlhaas, S., Stienen, D., Kurth, I., Hameister, H., Mangold, E., Kötting, J., Kaminsky, E., Propping, P., et al. (2009). Analysis of rare APC variants at the mRNA level: six pathogenic mutations and literature review. J. Mol. Diagn. *11*, 131–139.
62. McCart, A., Latchford, A., Volikos, E., Rowan, A., Tomlinson, I., and Silver, A. (2006). A novel exon duplication event leading to a truncating germ-line mutation of the APC gene in a familial adenomatous polyposis family. Fam. Cancer *5*, 205–208.
63. Plon, S.E., Eccles, D.M., Easton, D., Foulkes, W.D., Genuardi, M., Greenblatt, M.S., Hogervorst, F.B.L., Hoogerbrugge, N., Spurdle, A.B., Tavtigian, S.V.; and IARC Unclassified Genetic Variants Working Group (2008). Sequence variant classification and reporting: recommendations for improving the interpretation of cancer susceptibility genetic test results. Hum. Mutat. *29*, 1282–1291.
64. Tavtigian, S.V., Greenblatt, M.S., Harrison, S.M., Nussbaum, R.L., Prabhu, S.A., Boucher, K.M., Biesecker, L.G.; and ClinGen Sequence Variant Interpretation Working Group ClinGen SVI (2018). Modeling the ACMG/AMP variant classification guidelines as a Bayesian classification framework. Genet. Med. *20*, 1054–1060.
65. Minde, D.P., Anvarian, Z., Rüdiger, S.G., and Maurice, M.M. (2011). Messing up disorder: how do missense mutations in the tumor suppressor protein APC lead to cancer? Mol. Cancer *10*, 101.
66. Valle, L., Katz, L.H., Latchford, A., Mur, P., Moreno, V., Frayling, I.M., Heald, B., Capellá, G.; and InSiGHT Council (2023). Position statement of the International Society for Gastrointestinal Hereditary Tumours (InSiGHT) on APC I1307K and cancer risk. J. Med. Genet. *60*, 1035–1043.
67. Schmidt, R.J., Steeves, M., Bayrak-Toydemir, P., Benson, K.A., Coe, B.P., Conlin, L.K., Ganapathi, M., Garcia, J., Gollob, M.H., Jobanputra, V., et al. (2024). Recommendations for risk allele evidence curation, classification, and reporting from the ClinGen Low Penetrance/Risk Allele Working Group. Genet. Med. *26*, 101036.
68. Denham, N.C., Pearman, C.M., Ding, W.Y., Waktare, J., Gupta, D., Snowdon, R., Hall, M., Cooper, R., Modi, S., Todd, D., and Mahida, S. (2019). Systematic re-evaluation of SCN5A variants associated with Brugada syndrome. J. Cardiovasc. Electrophysiol. *30*, 118–127.
69. Thompson, B.A., Spurdle, A.B., Plazzer, J.P., Greenblatt, M.S., Akagi, K., Al-Mulla, F., Bapat, B., Bernstein, I., Capellá, G., den Dunnen, J.T., et al. (2014). Application of a 5-tiered scheme for standardized classification of 2,360 unique mismatch repair gene variants in the InSiGHT locus-specific database. Nat. Genet. *46*, 107–115.
70. Parsons, M.T., Tudini, E., Li, H., Hahnen, E., Wappenschmidt, B., Feliubadaló, L., Aalfs, C.M., Agata, S., Aittomäki, K., Alducci, E., et al. (2019). Large scale multifactorial likelihood quantitative analysis of BRCA1 and BRCA2 variants: An ENIGMA resource to support clinical variant classification. Hum. Mutat. *40*, 1557–1578.

The American Journal of Human Genetics, Volume *111*

Supplemental information

Large-scale application of ClinGen-InSiGHT *APC*-specific ACMG/AMP variant classification criteria leads to substantial reduction in VUS

Xiaoyu Yin, Marcy Richardson, Andreas Laner, Xuemei Shi, Elisabet Ognedal, Valeria Vasta, Thomas v.O. Hansen, Marta Pineda, Deborah Ritter, Johan de Dunnen, Emadeldin Hassanin, Wencong Lyman Lin, Ester Borras, Karl Krahn, Margareta Nordling, Alexandra Martins, Khalid Mahmood, Emily Nadeau, Victoria Beshay, Carli Tops, Maurizio Genuardi, Tina Pesaran, Ian M. Frayling, Gabriel Capellá, Andrew Latchford, Sean V. Tavtigian, Carlo Maj, Sharon E. Plon, Marc S. Greenblatt, Finlay A. Macrae, Isabel Spier, and Stefan Aretz

Figure S1 (A) Number of *APC* variants (%) in the ClinVar and their original and revised classifications

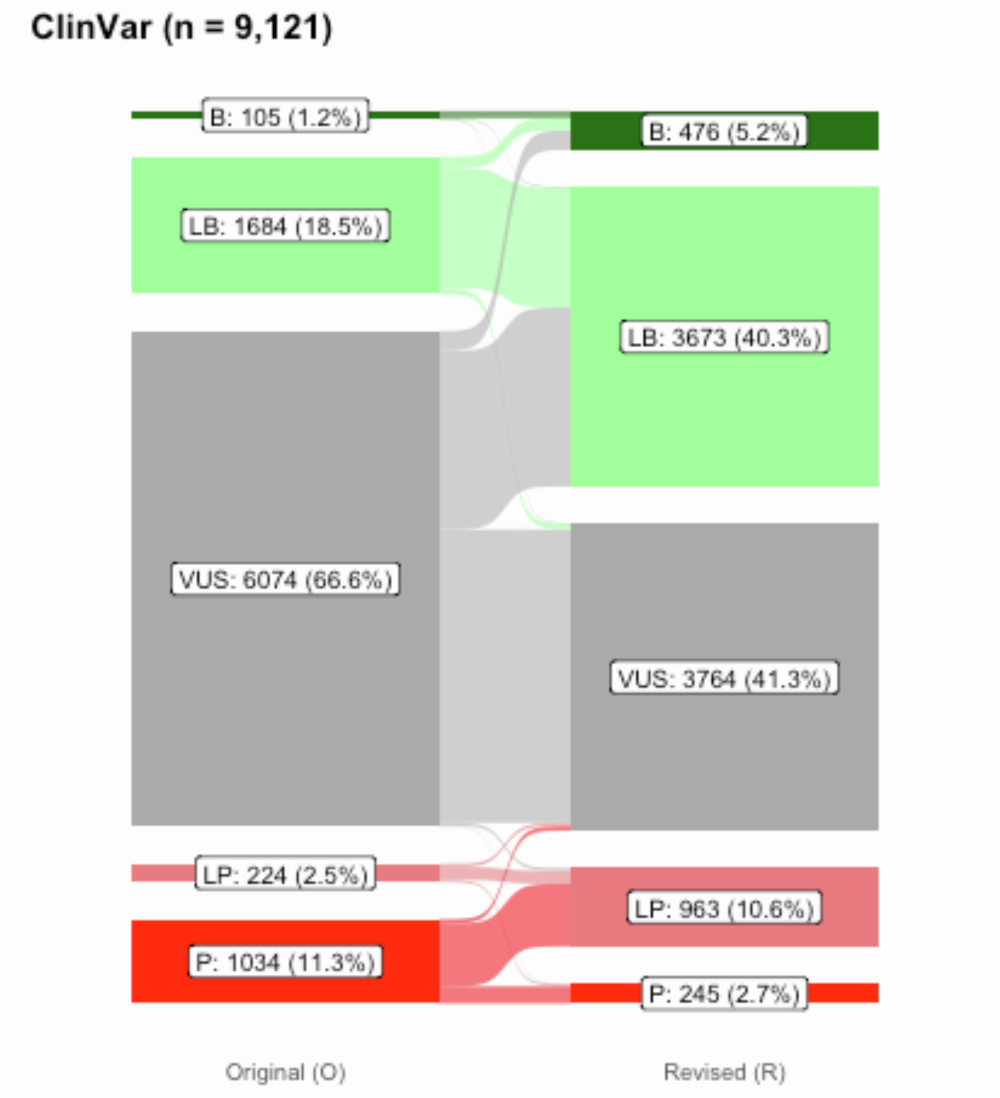

	Revised P	Revised LP	Revised VUS	Revised LB	Revised B
Original P	212 (20.5%)	768 (74.3%)	54 (5.2%)	0 (0%)	0 (0%)
Original LP	24 (10.7%)	159 (71%)	40 (17.9%)	1 (0.4%)	0 (0%)
Original VUS	9 (0.1%)	36 (0.6%)	3597 (59.2%)	2193 (36.1%)	239 (3.9%)
Original LB	0 (0%)	0 (0%)	66 (3.9%)	1471 (87.4%)	147 (8.7%)
Original B	0 (0%)	0 (0%)	7 (6.7%)	8 (7.6%)	90 (85.7%)
Total	245 (2.7%)	963 (10.6%)	3764 (41.3%)	3673 (40.3%)	476 (5.2%)

(B) Number of *APC* variants (%) in the InSiGHT LOVD and their original and revised classifications

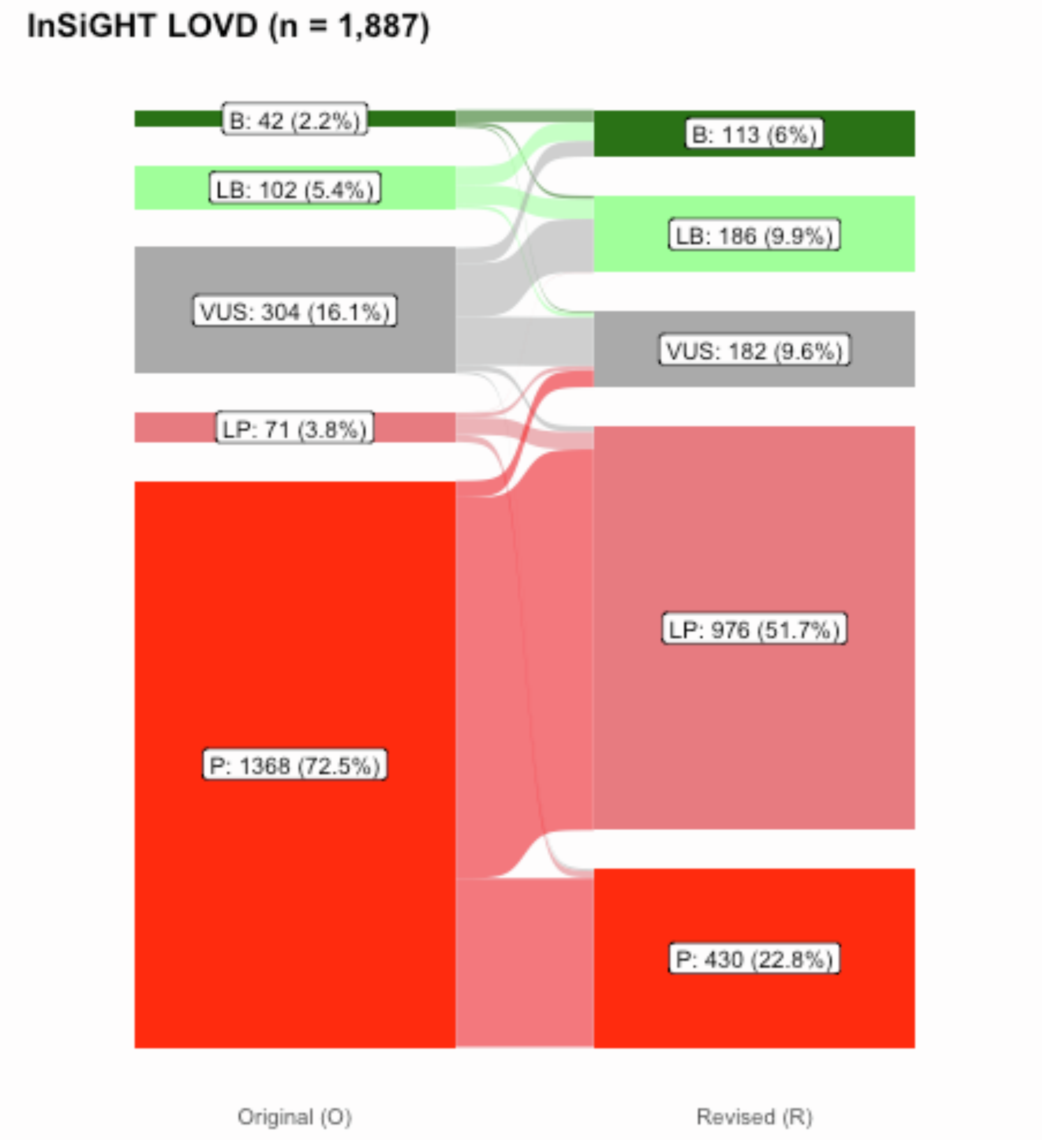

	Revised P	Revised LP	Revised VUS	Revised LB	Revised B
Original P	408 (29.8%)	920 (67.3%)	40 (2.9%)	0 (0%)	0 (0%)
Original LP	17 (23.9%)	42 (59.2%)	11 (15.5%)	1 (1.4%)	0 (0%)
Original VUS	5 (1.6%)	14 (4.6%)	118 (38.8%)	129 (42.4%)	38 (12.5%)
Original LB	0 (0%)	0 (0%)	9 (8.8%)	48 (47.1%)	45 (44.1%)
Original B	0 (0%)	0 (0%)	4 (9.5%)	8 (19%)	30 (71.4%)
Total	430 (22.8%)	976 (51.7%)	182 (9.6%)	186 (9.9%)	113 (6%)

Table S1 Status of ClinVar and all *APC* variant databases

Database name & URL	Curation & submission status	Date of Access	Submissions	Unique variant	Status
ClinVar https://www.ncbi.nlm.nih.gov/clinvar/?term=APC[gene]	Not curated, accepting submissions	21 March 2022	18029	9121	Active
Global Variome shared LOVD https://www.lovd.nl/apc; https://databases.lovd.nl/shared/genes/APC	Curated, accepting submissions	12 May 2022	5663	1877	Active
InSiGHT *APC* LOVD http://www.insight-database.org/genes/APC	Merged with Global Variome shared LOVD	12 May 2022	5663	1877	Active
The UMD *APC* mutations database http://www.umd.be/APC/	Curated, not accepting submissions	21 July 2022	3717	720	Active
Argentina National Institute of Cancer http://www.inc.gob.ar/sither/genes/APC	Curated, accepting submissions	21 July 2022	161	48	Active
Brazilian initiative on Precision Medicine<br>http://bipmed.iqm.unicamp.br/genes/APC<br>http://bipmed.iqm.unicamp.br/snparray/genes/APC<br>http://bipmed.iqm.unicamp.br/snparray_hg19/genes/APC<br>http://bipmed.iqm.unicamp.br/snparray_296/genes/APC<br>http://bipmed.iqm.unicamp.br/wes_hg19/genes/APC	Not curated, not accepting submissions	21 July 2022	54<br>23<br>23<br>23<br>74	54<br>23<br>23<br>23<br>74	Active
The *APC* mutation database http://fap.taenzer.me/	Inactive, URL not found				Inactive
Canadian Open Genetics Repository http://opengenetics.ca/#/brca/gene/APC	Curated, not accepting submissions	21 July 2022		259	Active
CanVas – A Greek Cancer Patient Genetic Variation Resource http://ithaka.rrp.demokritos.gr/CanVaS/genes/APC	Curated, accepting submissions	21 July 2022	593	177	Active
The Cyprus *APC* LOVD http://db.cshg.org.cy/genes/APC	Inactive, URL not found				Inactive
Dian Diagnostics & Zhejiang University Center for Genetic and Genomic Medicine http://www.genomed.org/lovd2/home.php?select_db=APC	Installation lost				Inactive
Iran Variation Database http://genet.ir/variome/genes/APC	Contained hidden *APC* variants only; LOVD installation partially broken				Inactive
Malaysian Node of the Human Variome Project Database http://www.kk.usm.my/LOVDv.3.0/genes/APC	Curated, accepting submissions	21 July 2022	35	29	Active
MexVar https://bipmed.fcm.unicamp.br/mexvar/genes/APC	Inactive, URL not found				Inactive
Nicaragua APC http://databases.lovd.ni/shared/genes/APC	Inactive, URL not found				Inactive
Spain MDB https://lovd3.isciii.es/genes/APC	Did not contain any *APC* variant record				Inactive
Zhejiang University-Adinovo Center APC Database http://databases.lovd.nl/genomed/home.php?select_db=APC	Inactive, URL not found				Inactive
Other LOVD installation – LOVD3 whole genome datasets http://databases.lovd.nl/whole_genome/	Not curated; not accepting submissions; imported from Exome Variant Server	21 July 2022	321	321	Active
Other LOVD installation – by the University of Melbourne http://proteomics.bio21.unimelb.edu.au/lovd/genes/APC	Not curated; inactive, not accepting submissions				Inactive

Table S3 Variants reclassified as VUS from clinically relevant classifications (B/LB/P/LP)

Deletion at the extremities of the gene with unclear molecular consequences on the protein structure

Database_ID	HGVSc; HGVSp	Predicted consequence	Prior classification	Reclassification by APC-specific criteria
ClinVar 495348	NM_000038.5:c.(?_-37541)_(-27791_?)del	deletion: promoter 1B	**Pathogenic**	VUS: PM2_supporting
ClinVar 832064	NC_000005.10:g.(?_112707312)_(112755035_?)del	deletion: promoter 1B and 1A, exons 2	**Pathogenic**	VUS: PM2_supporting
LOVD APC_001246	NM_001127511.3:c.165+17816_166-18738delinsTGCTCTATGACCAATT	deletion: promoter 1B deletion	**Pathogenic**	VUS: PM2_supporting
ClinVar 580918	NC_000005.9:g.112001178_112043328del42151	deletion: promoter 1B partial	Likely pathogenic	VUS: PM2_supporting
ClinVar 433562	NM_000038.5:c.-85-?_-19+?del	deletion: exon 1	**Pathogenic**	VUS: PM2_supporting
LOVD APC_000526	NM_000038.6:c.-85_(135+1_136-1)	deletion: exon 1-2	**Pathogenic**	VUS: PS4_supporting, PM2_supporting
ClinVar 1049778	NM_000038.6:c.-2_135+1824del	deletion: exon 2	**Pathogenic**	VUS: PM2_supporting
ClinVar 1049324	NM_000038.6:c.-2_135+1274del	deletion: exon 2	**Pathogenic**	VUS: PM2_supporting
ClinVar 1049076	NM_000038.6:c.-2_136-2903del	deletion: exon 2	**Pathogenic**	VUS: PM2_supporting
ClinVar 584665	NM_000038.6:c.1895_1958+28del	deletion: exon 15 partial	**Pathogenic**	VUS: PM2_supporting
ClinVar 433572	NM_000038.5:c.3146-?_8532+?del	deletion: exon 16 partial	**Pathogenic**	VUS: PM2_supporting
ClinVar 433571	NM_000038.5:c.2155-?_3960+?del	deletion: exon 16 partial	**Pathogenic**	VUS: PM2_supporting
ClinVar 1071358	NC_000005.9:g.(?_112174702)_112203173del	deletion: exon 16 partial	**Pathogenic**	VUS: PM2_supporting
ClinVar 1071357	NC_000005.9:g.(?_112173930)_112310702del	deletion: exon 16 partial	**Pathogenic**	VUS: PM2_supporting

Large duplication and complex variants with unknown impact on the reading frame

Database_ID	HGVSc; HGVSp	Predicted consequence	Prior classification	Reclassification by APC-specific criteria
ClinVar 1067201	NC_000005.9:g.(?_112072721)_(112090732_?)dup	duplication: promoter 1A, exon 2	**Likely pathogenic**	VUS: PM2_supporting
ClinVar 584349	NC_000005.9:g.(?_112072721)_(112090728_?)dup	duplication: promoter 1A, exon 2	**Likely pathogenic**	VUS: PM2_supporting
ClinVar 469686	NC_000005.9:g.(?_112072721)_(112111440_?)dup	duplication: promoter 1A, exons 2-5	**Likely pathogenic**	VUS: PM2_supporting
ClinVar 1067204	NC_000005.9:g.(?_112071797)_112137006dup	duplication: promoter 1A, exons 2-7, exon 8 partial	**Likely pathogenic**	VUS: PM2_supporting
ClinVar 1067082	NC_000005.9:g.(?_112090582)_(112157694_?)dup	duplication: exon 1-11	**Likely pathogenic**	VUS: PM2_supporting
ClinVar 58098	GRCh38/hg38 5q15-22.3(chr5:96454445-114050905)x3	duplication: exon 1-16	**Pathogenic**	VUS: PM2_supporting
ClinVar 394550	GRCh37/hg19 5q21.3-35.3(chr5:106716357-180687338)x3	duplication: exon 1-16	**Pathogenic**	VUS: PM2_supporting
ClinVar 425542	GRCh37/hg19 5q15-35.3(chr5:94844077-178830410)x3	duplication: exon 1-16	Likely benign	VUS: PM2_supporting
ClinVar 688598	GRCh37/hg19 5q14.3-23.3(chr5:89949118-129317455)x3	duplication: exon 1-16	**Pathogenic**	VUS: PM2_supporting
ClinVar 607687	GRCh37/hg19 5p15.33-q35.3(chr5:25328-180693344)x3	duplication: exon 1-16	**Pathogenic**	VUS: PM2_supporting
ClinVar 607681	GRCh37/hg19 5p15.33-q35.3(chr5:13648-180905029)x3	duplication: exon 1-16	**Pathogenic**	VUS: PM2_supporting
ClinVar 441919	GRCh37/hg19 5p15.33-q35.3(chr5:113577-180719789)x3	duplication: exon 1-16	**Pathogenic**	VUS: PM2_supporting
ClinVar 441920	GRCh37/hg19 5p15.33-q35.3(chr5:113577-180719789)	duplication: exon 1-16	**Pathogenic**	VUS: PM2_supporting
ClinVar 417556	NC_000005.9:g.(?_112090570)_(112157688_?)dup	duplication: exon 2-11	**Likely pathogenic**	VUS: PVS1_strong, PM2_supporting
ClinVar 469688	Single allele	duplication: exon 2-4	**Likely pathogenic**	VUS: PVS1_strong, PM2_supporting
ClinVar 830510	NC_000005.10:g.(?_112754891)_(112767400_?)dup	duplication: exons 2-4	**Likely pathogenic**	VUS: PVS1_strong, PM2_supporting
ClinVar 1067200	NC_000005.9:g.(?_112090582)_(112137086_?)dup	duplication: exon 2-8	**Likely pathogenic**	VUS: PVS1_strong, PM2_supporting
LOVD APC_001753	NM_000038.6:c.(135+1_136-1)_(422+1_423-1)dup	duplication: exon 3-4	**Pathogenic**	VUS: PVS1_strong, PM2_supporting
LOVD APC_000750	NM_000038.6:c.(422+1_423-1)_(531+1_532-1)dup	duplication: exon 4-5	**Pathogenic**	VUS: PVS1_strong, PM2_supporting
LOVD APC_001782	NM_000038.6:c.(834+1_835-1)_(1408+1_1409-1)dup	duplication: exon9-11	**Likely pathogenic**	VUS: PVS1_strong, PM2_supporting
ClinVar 1067081	NC_000005.9:g.(?_112170638)_(112170872_?)dup	duplication: exon 15	**Likely pathogenic**	VUS: PVS1_strong, PM2_supporting
LOVD APC_001415	NM_000038.6:c.1806_1817delinsN[300]	other	**Pathogenic**	VUS: PM2_supporting
LOVD APC_001953	complex 3.9mb rearrangement	other	**Likely pathogenic**	VUS: PS4_supporting, PM2_supporting
ClinVar 243004	NM_001127511.2:c.[-125delA;-195A>C]	other	**Pathogenic**	VUS: PM2_supporting

Variants at the 5' end of the gene and therefore excluded from the application of PVS1

Database_ID	HGVSc; HGVSp	Predicted consequences	Prior classification	Reclassification by APC-specific criteria
ClinVar 243007	NM_001127511.3:c.-192A>T	UTR	**Pathogenic**	VUS: BP4, PM2_supporting
ClinVar 243006	NM_001127511.3:c.-192A>G	UTR	**Likely pathogenic**	VUS: BS1
ClinVar 652807	NM_001127511.3:c.-192_-191delinsTAGCAAGGG	UTR	**Likely pathogenic**	VUS: PM2_supporting
ClinVar 243005	NM_001127511.3:c.-191T>C	UTR	**Pathogenic**	VUS: BP4, PM2_supporting
LOVD APC_001802	NM_000038.6:c.-190G>A	UTR	**pathogenic**	VUS: PM2_supporting
ClinVar 1050412	NM_001127511.3:c.166-28469_166-27547del	UTR	**Pathogenic**	VUS: PM2_supporting
ClinVar 1050584	NM_001127511.3:c.166-28467del	UTR	**Pathogenic**	VUS: BP4, PM2_supporting
ClinVar 537477	NM_000038.6:c.14del; NP_000029.2:p.Ser5TyrfsTer6	frameshift	**Pathogenic**	VUS: PM2_supporting
ClinVar 630969	NM_000038.6:c.26_27insTTTA; NP_000029.2:p.Leu9PhefsTer7	frameshift	**Pathogenic**	VUS: PM2_supporting
ClinVar 628229	NM_000038.6:c.32_33insA; NP_000029.2:p.Gln12AlafsTer3	frameshift	**Pathogenic**	VUS : PM2_supporting
ClinVar 970282	NM_000038.6:c.55G>T; NP_000029.2:p.Glu19Ter	nonsense	**Pathogenic**	VUS: PM2_supporting
ClinVar 470090	NM_000038.6:c.74_75del; NP_000029.2:p.Gln25ArgfsTer5	frameshift	**Pathogenic**	VUS: PM2_supporting
ClinVar 428113	NM_000038.6:c.93del; NP_000029.2:p.Asn32IlefsTer13	frameshift	**Pathogenic**	VUS: PM2_supporting
ClinVar, LOVD 428154	NM_000038.6:c.104del; NP_000029.2:p.Thr35LysfsTer10	frameshift	**Pathogenic**	VUS: PM2_supporting
ClinVar 537502	NM_000038.6:c.108del; NP_000029.2:p.Lys36AsnfsTer9	frameshift	**Pathogenic**	VUS: PM2_supporting
ClinVar 579756	NM_000038.6:c.132dup; NP_000029.2:p.Lys45GlufsTer5	frameshift	**Pathogenic**	VUS: PM2_supporting

Variants that satisfied PVS1, however were present at very low frequencies in population reference database (PM2_supporting not met)

Database_ID	HGVSc; HGVSp	Predicted consequences	Prior classification	Reclassification by APC-specific criteria
ClinVar 654864	NM_000038.6:c.156del; NP_000029.2:p.Gly53GlufsTer17	frameshift	**Pathogenic**	VUS: PVS1
ClinVar, LOVD 934724	NM_000038.6:c.203del; NP_000029.2:p.Leu68TyrfsTer2	frameshift	**Pathogenic**	VUS: PVS1
ClinVar, LOVD 411479	NM_000038.6:c.471G>A; NP_000029.2:p.Trp157Ter	nonsense	**Pathogenic**	VUS: PVS1
ClinVar, LOVD 955439	NM_000038.6:c.1042C>T; NP_000029.2:p.Arg348Ter	nonsense	**Pathogenic**	VUS: PVS1
ClinVar 438865	NM_000038.6:c.1333C>T; NP_000029.2:p.Gln445Ter	nonsense	**Pathogenic**	VUS: PVS1, BS1
ClinVar, LOVD 183857	NM_000038.6:c.4669_4670del; NP_000029.2:p.Ile1557Ter	frameshift	**Pathogenic**	VUS: PVS1
ClinVar, LOVD 230520	NM_000038.6:c.5038C>T; NP_000029.2:p.Gln1680Ter	nonsense	**Likely pathogenic**	VUS: PVS1, BS1
ClinVar 428166	NM_000038.6:c.6905C>G; NP_000029.2:p.Ser2302Ter	nonsense	**Pathogenic**	VUS: PVS1
ClinVar 653103	NM_000038.6:c.7489_7490insT; NP_000029.2:p.Ser2497PhefsTer14	frameshift	**Pathogenic**	VUS: PVS1, BS1
ClinVar 827255	NM_000038.6:c.7798_7801del; NP_000029.2:p.Gln2600ValfsTer15	frameshift	**Pathogenic**	VUS: PVS1, BS1
ClinVar 648862	NM_000038.6:c.7803_7807del; NP_000029.2:p.Ser2601ArgfsTer17	frameshift	**Likely pathogenic**	VUS: PVS1

Missense variants that are unable to be classified, mainly because minor allele frequency thresholds for BA1/BS1 are not met and lack of additional information

Database ID	HGVSc; HGVSp	Predicted consequences	Prior classification	Reclassification by APC-specific criteria
LOVD APC_001042	NM_000038.6:c.446A>T; NP_000029.2:p.Asp149Val	missense	**pathogenic**	VUS: BP1, PM2_supporting, PS4_supporting
LOVD APC_000758	NM_000038.6:c.623A>G; NP_000029.2:p.Gln208Arg	missense	**pathogenic**	VUS: BP1, PM2_supporting, PP1
LOVD APC_000627	NM_000038.6:c.1060C>T; NP_000029.2:p.Pro354Ser	missense	**pathogenic**	VUS: BP1, PM2_supporting
ClinVar 231954	NM_000038.6:c.1902T>G; NP_000029.2:p.Ser634Arg	missense	**Likely pathogenic**	VUS: BP1, PS3_moderate
ClinVar 428167	NM_000038.6:c.3077A>C; NP_000029.2:p.Asn1026Thr	missense	**Likely pathogenic**	VUS : PM2_supporting, PM5_supporting
ClinVar 802	NM_000038.6:c.3359G>A; NP_000029.2:p.Gly1120Glu	missense	**Pathogenic**	VUS: BP1, PM2_supporting
ClinVar 817	NM_000038.6:c.4183A>T; NP_000029.2:p.Ser1395Cys	missense	**Pathogenic**	VUS: BP1, PM2_supporting
LOVD APC_000208	NM_000038.6:c.4549C>G; NP_000029.2:p.Gln1517Glu	missense	**pathogenic**	VUS: BP1, PM2_supporting, PS4_supporting
LOVD APC_001630	NM_000038.6:c.6257C>A; NP_000029.2:p.Pro2086Gln	missense	**Likely pathogenic**	VUS: BP1, PM2_supporting
ClinVar 486771	NM_000038.6:c.743A>G; NP_000029.2:p.Asn248Ser	missense	Likely benign	VUS: BP1
ClinVar 827113	NM_000038.6:c.754A>G; NP_000029.2:p.Thr252Ala	missense	Likely benign	VUS : BP1, PM2_supporting
ClinVar 487015	NM_000038.6:c.2581G>A; NP_000029.2:p.Gly861Ser	missense	Likely benign	VUS: BP1, PM2_supporting
LOVD APC_001944	NM_000038.6:c.2605A>G; NP_000029.2:p.Asn869Asp	missense	Likely benign	VUS: BP1, PM2_supporting
ClinVar 617980	NM_000038.6:c.2651C>T; NP_000029.2:p.Ala884Val	missense	Likely benign	VUS: BP1, PM2_supporting
ClinVar 428175	NM_000038.6:c.2909G>C; NP_000029.2:p.Ser970Thr	missense	Likely benign	VUS: BP1, PM2_supporting
LOVD APC_001667	NM_000038.6:c.3290A>G; NP_000029.2:p.Glu1097Gly	missense	Likely benign	VUS: BP1, PM2_supporting
ClinVar 482305	NM_000038.6:c.3608G>T; NP_000029.2:p.Gly1203Val	missense	Likely benign	VUS: BP1, PM2_supporting
LOVD APC_001945	NM_000038.6:c.3930G>T; NP_000029.2:p.Lys1310Asn	missense	Likely benign	VUS: BP1, PM2_supporting
LOVD APC_001676	NM_000038.6:c.4316C>G; NP_000029.2:p.Pro1439Arg	missense	Likely benign	VUS: BP1, PM2_supporting
ClinVar 824838	NM_000038.6:c.4370C>T; NP_000029.2:p.Ala1457Val	missense	Likely benign	VUS: BP1, PM2_supporting
LOVD APC_001865	NM_000038.6:c.5249T>C; NP_000029.2:p.Val1750Ala	missense	Likely benign	VUS: BP1, PM2_supporting
ClinVar 482255	NM_000038.6:c.5651C>G; NP_000029.2:p.Ala1884Gly	missense	Likely benign	VUS: BP1
LOVD APC_001947	NM_000038.6:c.5731C>A; NP_000029.2:p.Gln1911Lys	missense	Likely benign	VUS: BP1, PM2_supporting
ClinVar 617981	NM_000038.6:c.5839A>C; NP_000029.2:p.Thr1947Pro	missense	Likely benign	VUS: BP1, PM2_supporting
ClinVar 236654	NM_000038.6:c.8255A>C; NP_000029.2:p.Asn2752Thr	missense	Benign	VUS: BP1

Variants in the flanking intronic region with unknown consequences

Database ID	HGVSc; HGVSp	Predicted consequences	Prior classification	Reclassification by APC-specific criteria
ClinVar 482476	NM_000038.6:c.135+1G>T	splice	**Likely pathogenic**	VUS: PP3, PM2_supporting
ClinVar 490194	NM_000038.6:c.135+2T>C	splice	**Likely pathogenic**	VUS: PP3, PM2_supporting
ClinVar, LOVD 469955	NM_000038.6:c.423-9A>G	splice	**Pathogenic**	VUS: PP3, PM2_supporting, PS3_moderate
LOVD APC_000602	NM_000038.6:c.423-6_424delinsGAAGCAAGATCAG	splice	**pathogenic**	VUS: PM2_supporting, PS4_supporting
LOVD APC_000624	NM_000038.6:c.531+1del	splice	**pathogenic**	VUS: PP3, PM2_supporting, PM6, PS4_supporting
ClinVar, LOVD 537529	NM_000038.6:c.531+5_531+8del	splice	**Likely pathogenic**	VUS: PP3, PM2_supporting, PS3_moderate, PS4_supporting
ClinVar, LOVD 428099	NM_000038.6:c.531+3A>C	splice	**Likely pathogenic**	VUS: PP3, PM2_supporting
ClinVar 127305	NM_000038.6:c.531+5G>A	splice	**Pathogenic**	VUS: PP3, PM2_supporting, PS4_supporting, PS1_moderate
LOVD APC_001369	NM_000038.6:c.532-2_532-1insAAAC	splice	**pathogenic**	VUS: BP4, PM2_supporting
LOVD APC_000429	NM_000038.6:c.645+1G>C	splice	**pathogenic**	VUS: PVS1_moderate, PM2_supporting, PS1_moderate
LOVD APC_001803	NM_000038.6:c.645+2T>C	splice	**pathogenic**	VUS: PVS1_moderate, PM2_supporting, PS1_moderate
ClinVar 185659	NM_000038.6:c.645+2T>G	splice	**Likely pathogenic**	VUS: PVS1_moderate, PM2_supporting, PS1_moderate
ClinVar 822326	NM_000038.6:c.835-17A>G	splice	**Likely pathogenic**	VUS: PM2_supporting, PS3_moderate
ClinVar, LOVD 433614	NM_000038.6:c.835-7T>G	splice	**Likely pathogenic**	VUS: PP3, PM2_supporting, PS3_moderate
LOVD APC_000327	NM_000038.6:c.933+1del	splice	**pathogenic**	VUS: PP3, PM2_supporting, PS4_supporting
ClinVar 181775	NM_000038.6:c.1312+3_1312+4del	splice	**Pathogenic**	VUS: PP3, PM2_supporting
ClinVar 486792	NM_000038.6:c.1312+3A>C	splice	**Likely pathogenic**	VUS: PP3, PM2_supporting, PS1_moderate
LOVD APC_001939	NM_000038.6:c.1312+4_1312+19del	splice	**pathogenic**	VUS: BP4, PM2_supporting
ClinVar, LOVD 265372	NM_000038.6:c.1312+5G>C	splice	**Likely pathogenic**	VUS: PM2_supporting, PS4_supporting, PS1_moderate
LOVD APC_001275	NM_000038.6:c.1312+8C>T	splice	**pathogenic**	VUS: BP4, PM2_supporting
ClinVar 1066995	NM_000038.6:c.1313-2A>C	splice	**Likely pathogenic**	VUS: PP3, PM2_supporting
ClinVar 578480	NM_000038.6:c.1313-2A>G	splice	**Likely pathogenic**	VUS: PP3, PM2_supporting, PS3_moderate
ClinVar 1339656	NM_000038.6:c.1313-1G>T	splice	**Likely pathogenic**	VUS: PP3, PM2_supporting

ClinVar 1323307	NM_000038.6:c.1313-1G>C	splice	**Pathogenic**	VUS: PP3, PM2_supporting
LOVD APC_000792	NM_000038.6:c.1408+7C>G	splice	**pathogenic**	VUS: BP4, PM2_supporting, PS4_supporting
ClinVar, LOVD 411406	NM_000038.6:c.1409-5A>G	splice	**Pathogenic**	VUS: PP3, PM2_supporting, PS3_moderate, PS4_supporting
ClinVar, LOVD 485146	NM_000038.6:c.1409-3T>G	splice	**Likely pathogenic**	VUS: PP3, PM2_supporting, PS3_moderate
LOVD APC_001291	NM_000038.6:c.1548_1548+1delinsTT	splice	**pathogenic**	VUS: BP4, PM2_supporting
LOVD APC_001083	NM_000038.6:c.1548+1del	splice	**pathogenic**	VUS: PP3, PM2_supporting
LOVD APC_001292	NM_000038.6:c.1548+1_1548+9del	splice	**pathogenic**	VUS: PP3, PM2_supporting
ClinVar 265375	NM_000038.6:c.1548+3_1548+4del	splice	**Likely pathogenic**	VUS: PP3, PM2_supporting
LOVD APC_001727	NM_000038.6:c.1549-3C>G	splice	**Likely pathogenic**	VUS: PP3, PM2_supporting
LOVD APC_000214	NM_000038.6:c.1627-8A>G	splice	**pathogenic**	VUS: PP3, PM2_supporting
LOVD APC_000321	NM_000038.6:c.1742A>G; NP_000029.2:p.Lys581Arg	splice	**pathogenic**	VUS: PM2_supporting, PS3_moderate
ClinVar 428153	NM_000038.6:c.1743G>C; NP_000029.2:p.Lys581Asn	splice	**Likely pathogenic**	VUS: PVS1_strong, PM2_supporting
LOVD APC_001411	NM_000038.6:c.1743+1del	splice	**pathogenic**	VUS: PP3, PM2_supporting
LOVD APC_000296	NM_000038.6:c.1744-17_1744-5delinsTC	splice	**pathogenic**	VUS: BP4, PM2_supporting
ClinVar 664704	NM_000038.6:c.1744-6_1744-4delinsAG	splice	**Pathogenic**	VUS: PP3, PM2_supporting
ClinVar 433625	NM_000038.6:c.1744-4C>G	splice	**Likely pathogenic**	VUS: PM2_supporting
ClinVar 819988	NM_000038.6:c.1744-3T>G	splice	**Pathogenic**	VUS: PP3, PM2_supporting
LOVD APC_001467	NM_000038.6:c.1954_1958+15del	splice	**pathogenic**	VUS: BP4, PM2_supporting
LOVD APC_001445	NM_000038.6:c.1958+1del	splice	**pathogenic**	VUS: PP3, PM2_supporting
ClinVar 265560	NM_000038.6:c.1958+3A>T	splice	**Likely pathogenic**	VUS: PP3, PM2_supporting, PS1_moderate
ClinVar 439406	NM_000038.6:c.-18-13T>G	splice	Benign	VUS: PM2_supporting
ClinVar 672308	NM_000038.6:c.135+6A>G	splice	Likely benign	VUS: BP4, PM2_supporting
ClinVar 1130754	NM_000038.6:c.135+7A>G	splice	Likely benign	VUS: BP4, PM2_supporting
ClinVar 918312	NM_000038.6:c.136-13T>G	splice	Likely benign	VUS: BP4, PM2_supporting
ClinVar 490196	NM_000038.6:c.136-12T>C	splice	Likely benign	VUS: BP4, PM2_supporting
ClinVar 490239	NM_000038.6:c.221-11A>T	splice	Likely benign	VUS: BP4, PM2_supporting
LOVD APC_001759	NM_000038.6:c.423-18_423-17insA	splice	benign	VUS: BP4, PM2_supporting
ClinVar 181780	NM_000038.6:c.423-17_423-16insT	splice	Benign	VUS: BP4, PM2_supporting
ClinVar 1143398	NM_000038.6:c.423-9A>T	splice	Likely benign	VUS: BP4, PM2_supporting
ClinVar 1116379	NM_000038.6:c.423-8A>T	splice	Likely benign	VUS: BP4, PM2_supporting
ClinVar 1149314	NM_000038.6:c.423-4A>T	splice	Likely benign	VUS: BP4, PM2_supporting
ClinVar 537617	NM_000038.6:c.532-7G>T	splice	Likely benign	VUS: BP4
ClinVar 380375	NM_000038.6:c.532-7G>C	splice	Likely benign	VUS: BP4
ClinVar 1110067	NM_000038.6:c.645+7T>C	splice	Likely benign	VUS: BP4, PM2_supporting
ClinVar 1137468	NM_000038.6:c.645+8A>C	splice	Likely benign	VUS: BP4, PM2_supporting
ClinVar 926761	NM_000038.6:c.645+8A>T	splice	Likely benign	VUS: BP4, PM2_supporting
ClinVar 793315	NM_000038.6:c.646-8T>C	splice	Likely benign	VUS: BP4, PM2_supporting
ClinVar 490358	NM_000038.6:c.729+3T>A	splice	Likely benign	VUS: BP4, PM2_supporting
ClinVar 1103461	NM_000038.6:c.729+8A>G	splice	Likely benign	VUS: BP4, PM2_supporting

Deep intronic, synonymous, in-frame variants and variants in the UTR that are unable to be classified due to missing additional information

**Database_ID**	**HGVSc; HGVSp**	**Predicted consequences**	**Prior classification**	**Reclassification by APC-specific criteria**
LOVD APC_001244	NM_000038.6:c.1408+735A>T	intron	**pathogenic**	VUS: PM2_supporting, PS3_moderate, PS4_supporting
ClinVar 823173	NM_000038.6:c.933+829A>G	intron	**Likely pathogenic**	VUS: PM2_supporting
ClinVar 1111220	NM_001127511.3:c.-199C>T	UTR	Likely benign	VUS: BP4, PM2_supporting
ClinVar 1169564	NM_001127511.3:c.-167_-166insG	UTR	Benign	VUS: BP4, PM2_supporting
ClinVar 641353	NM_001127511.3:c.-134_-133insGGG	UTR	Likely benign	VUS: BP4, PM2_supporting
ClinVar 1164593	NM_001127511.3:c.-133_-132delinsGT	UTR	Benign	VUS: PM2_supporting
LOVD APC_001812	NM_001127511.3:c.-126dup	UTR	Likely benign	VUS: PM2_supporting
ClinVar 1316326	NM_001127511.3:c.-126_-125insGA	UTR	Likely benign	VUS: BP4, PM2_supporting
ClinVar 469825	NM_001127511.3:c.-124C>G	UTR	Benign	VUS: BP4, PM2_supporting
LOVD APC_001815	NM_001127511.3:c.15G>C; NP_001120983.2:p.Gly6=	UTR	Likely benign	VUS: PM2_supporting
ClinVar 918662	NM_000038.6:c.730-19G>T	intron	Likely benign	VUS: BP4, PM2_supporting
ClinVar 796802	NM_000038.6:c.834+7A>G	splice	Likely benign	VUS: BP4, PM2_supporting
ClinVar 628015	NM_000038.6:c.835-20A>C	intron	Likely benign	VUS: BP4, PM2_supporting
ClinVar 381344	NM_000038.6:c.835-15G>A	splice	Likely benign	VUS: BP4, PM2_supporting
ClinVar 1332031	NM_000038.6:c.835-13G>A	splice	Likely benign	VUS: BP4, PM2_supporting
ClinVar 1115430	NM_000038.6:c.835-10T>C	splice	Likely benign	VUS: PM2_supporting
LOVD APC_000439	NM_000038.6:c.933G>A; NP_000029.2:p.Lys311=	splice	benign	VUS: PVS1_supporting, PM2_supporting
ClinVar 927827	NM_000038.6:c.934-7C>T	splice	Likely benign	VUS: BP4, PM2_supporting
ClinVar 823565	NM_000038.6:c.993G>T; NP_000029.2:p.Ser331=	synonymous	Likely benign	VUS
ClinVar 734263	NM_000038.6:c.1071C>A; NP_000029.2:p.Ile357=	synonymous	Likely benign	VUS: PM2_supporting
ClinVar 1119186	NM_000038.6:c.1312+8C>G	splice	Likely benign	VUS: BP4, PM2_supporting
ClinVar 928340	NM_000038.6:c.1313-15G>T	splice	Likely benign	VUS: BP4, PM2_supporting
ClinVar 921987	NM_000038.6:c.1313-14del	splice	Likely benign	VUS: BP4, PM2_supporting

ClinVar 918459	NM_000038.6:c.1313-13T>C	splice	Likely benign	VUS: BP4
ClinVar 416761	NM_000038.6:c.1313-8T>A	splice	Likely benign	VUS: BP4, PM2_supporting
ClinVar 819130	NM_000038.6:c.1407A>G; NP_000029.2:p.Leu469=	splice	Likely benign	VUS: BP4
ClinVar 919829	NM_000038.6:c.1408+7C>T	splice	Likely benign	VUS: BP4, PM2_supporting
ClinVar 1128391	NM_000038.6:c.1408+8A>T	splice	Likely benign	VUS: BP4, PM2_supporting
ClinVar 388511	NM_000038.6:c.1408+8A>G	splice	Likely benign	VUS: BP4, PM2_supporting
ClinVar 371848	NM_000038.6:c.1409-17T>G	splice	Likely benign	VUS: BP4
ClinVar 923782	NM_000038.6:c.1409-16G>A	splice	Likely benign	VUS: BP4
ClinVar 922204	NM_000038.6:c.1409-16G>C	splice	Likely benign	VUS: BP4, PM2_supporting
ClinVar 508018	NM_000038.6:c.1409-13C>G	splice	Likely benign	VUS: BP4, PM2_supporting
ClinVar 922965	NM_000038.6:c.1410G>T; NP_000029.2:p.Gly470=	splice	Likely benign	VUS: BP4, PM2_supporting
ClinVar 630114	NM_000038.6:c.1626+8T>G	splice	Likely benign	VUS: BP4, PM2_supporting
ClinVar 490219	NM_000038.6:c.1626+8T>C	splice	Likely benign	VUS: BP4, PM2_supporting
ClinVar 490220	NM_000038.6:c.1627-17A>G	splice	Likely benign	VUS: PM2_supporting
ClinVar 627826	NM_000038.6:c.1627-16A>C	splice	Likely benign	VUS: BP4, PM2_supporting
ClinVar 490223	NM_000038.6:c.1744-20C>T	intron	Likely benign	VUS: BP4, PM2_supporting
ClinVar 548881	NM_000038.6:c.1744-14_1744-13del	splice	Likely benign	VUS: BP4, PM2_supporting
LOVD APC_001729	NM_000038.6:c.1744-11T>G	splice	Likely benign	VUS: PM2_supporting
ClinVar 516255	NM_000038.6:c.1744-10T>C	splice	Likely benign	VUS: BP4, PM2_supporting
LOVD APC_000607	NM_000038.6:c.1869G>T; NP_000029.2:p.Arg623=	synonymous	benign	VUS: BP4, BP7, PM2_supporting, PS3_moderate, PS4
ClinVar 627857	NM_000038.6:c.1959-18C>G	intron	Likely benign	VUS: BP4, PM2_supporting
ClinVar 630908	NM_000038.6:c.1959-17T>C	splice	Likely benign	VUS: BP4, PM2_supporting
LOVD APC_000458	NM_000038.6:c.1959G>C; NP_000029.2:p.Arg653Ser	splice	benign	VUS: PM2_supporting
ClinVar 389718	NM_000038.6:c.1962A>G; NP_000029.2:p.Gln654=	synonymous	Likely benign	VUS: PM2_supporting
ClinVar 733258	NM_000038.6:c.2031C>G; NP_000029.2:p.Val677=	synonymous	Likely benign	VUS
ClinVar 918747	NM_000038.6:c.4966_4967delinsAG; NP_000029.2:p.Ser1656=	synonymous	Likely benign	VUS: PM2_supporting
ClinVar 439411	NM_000038.6:c.5265_5268delinsATCG; NP_000029.2:p.AlaSer1755=	synonymous	Benign	VUS: PM2_supporting
ClinVar 757607	NM_000038.6:c.7404_7406del; NP_000029.2:p.Ser2469del	inframe	Likely benign	VUS: BP4, PM2_supporting
ClinVar 802150	NM_000038.6:c.*415_*414insAAAAAA	UTR	Likely benign	VUS: PM2_supporting
ClinVar 217930	NM_000038.6:c.1525_1527del; NP_000029.2:p.Thr509del	inframe	**Likely pathogenic**	VUS: BP4, PM2_supporting
LOVD APC_000075	NM_000038.6:c.2546_2551del; NP_000029.2:p.Asp849_Ser851delinsGly	inframe	**pathogenic**	VUS: BP4, PM2_supporting, PS4_moderate
LOVD APC_000883	NM_000038.6:c.3542_3568del; NP_000029.2:p.Leu1181_Ser1189del	inframe	**pathogenic**	VUS: BP4, PM2_supporting
Truncating variants at the 3' end of the gene and therefore excluded from the application of PVS1				
**Database_ID**	**HGVSc; HGVSp**	**Predicted**	**Prior_classification**	**Reclassification by APC-specific criteria**
ClinVar 545875	NM_000038.6:c.7946_7955del; NP_000029.2:p.Pro2649LeufsTer8	frameshift	**Pathogenic**	VUS: PM2_supporting
ClinVar 545737	NM_000038.6:c.7959_7962del; NP_000029.2:p.Thr2654ArgfsTer5	frameshift	**Pathogenic**	VUS: PM2_supporting
ClinVar 428168	NM_000038.6:c.8047del; NP_000029.2:p.Ile2683LeufsTer40	frameshift	**Pathogenic**	VUS: PM2_supporting
ClinVar 827446	NM_000038.6:c.8099_8102del; NP_000029.2:p.Asn2700ArgfsTer22	frameshift	**Likely pathogenic**	VUS: PM2_supporting
LOVD APC_001492	NM_000038.6:c.8344del; NP_000029.2:p.Thr2782LeufsTer28	frameshift	**pathogenic**	VUS: PM2_supporting
ClinVar, LOVD 486740	NM_000038.6:c.8514C>A; NP_000029.2:p.Tyr2838Ter	nonsense	**Likely pathogenic**	VUS: PM2_supporting
ClinVar 233392	NM_000038.6:c.8514C>G; NP_000029.2:p.Tyr2838Ter	nonsense	**Likely pathogenic**	VUS: PM2_supporting

Note: Classification based on the ClinGen InSiGHT Hereditary Colorectal Cancer/Polyposis Expert Panel Specifications to the ACMG/AMP Variant Interpretation Guidelines for APC Version 1.0.0. Criteria applied in a stepwise fashion using a classification algorithm, not all available evidence may have been considered to arrive at current classification. All classifications are preliminary.

Table S4 (A) Frequency of application of the APC-specific variant classification criteria

Criteria	Variant count for each criteria applied						
Code_weight	supporting	moderate	strong	very strong	stand alone	Total count	Total %
PM2_supporting	7083					7083	69.3%
BP1	4220					4220	41.3%
BP4	2789					2789	27.3%
BP7	2236					2236	21.9%
PVS1_variable	1	5	22	2164		2192	21.4%
BS1			2089			2089	20.4%
PS4_variable	281	93	68	12		454	4.4%
BA1					427	427	4.2%
BS2_variable	109		46			155	1.5%
PP3	60					60	0.6%
PS3_variable	7	32	5	4		48	0.5%
PS1_variable		38	3			41	0.4%
PS2_variable		15	1			16	0.2%
PM6_variable	5	9	0	1		15	0.1%
No code applied						11	0.1%
BS3_variable	10		0			10	0.1%
PP1_variable	5	1	2			8	0.1%
BP5	8					8	0.1%
PM5_variable	5	0				5	0.0%
BS4_variable	2		0			2	0.0%
BP2	1					1	0.0%

Table S4(B) Most frequent APC-specific variant classification codes and code combinations resulting in non-VUS classifications

Codes	Classification outcome	Number of variants	% of all variants (n=10,228)	Number of previous VUS	% of all previous VUS (n=6,142) [d]	McNemar's χ2 (p value)
**PVS1, PM2_supporting** [a]	Likely pathogenic	2138	20.9%	46	0.7%	40.02 ($p < 0.05$)
**BS1** [b]	Likely benign	2071	20.2%	1542	25.1%	1540 ($p < 0.05$)
**BP4, BP7** [c]	Likely benign	1669	16.3%	687	11.2%	685 ($p < 0.05$)
**BA1**	Benign	427	4.2%	208	3.4%	206 ($p < 0.05$)

[a] for 431 of these variants one additional code with at least supporting evidence could be applied (e. g. PS4_supporting), resulting in a final classification as pathogenic

[b] for 1881 of these variants additional codes could be applied (BP1, BP4, BP7 and/or BS2_supporting) without influence on the classification outcome; for 53 variants at least on additional strong code (BS2) could be applied, resulting in a classification as benign

[c] for 1505 of these variants, PM2_supporting was applicable, which was not considered as a conflict based on the APC-specific criteria

[d] % of all VUS that could be reclassified into the respective meaningful class based on this code (combination)
